# Effects of Spermidine Supplementation on Cognition and Biomarkers in Older Adults With Subjective Cognitive Decline: A Randomized Clinical Trial

**DOI:** 10.1001/jamanetworkopen.2022.13875

**Published:** 2022-05-26

**Authors:** Claudia Schwarz, Gloria S. Benson, Nora Horn, Katharina Wurdack, Ulrike Grittner, Ralph Schilling, Stefanie Märschenz, Theresa Köbe, Sebastian J. Hofer, Christoph Magnes, Slaven Stekovic, Tobias Eisenberg, Stephan J. Sigrist, Dietmar Schmitz, Miranka Wirth, Frank Madeo, Agnes Flöel

**Affiliations:** 1 Department of Neurology, Charité–Universitätsmedizin Berlin, corporate member of Freie Universität Berlin and Humboldt-Universität zu Berlin, Berlin, Germany; 2 Institute for Molecular Medicine Finland, HiLIFE, University of Helsinki, Helsinki, Finland; 3 NeuroCure Clinical Research Center, Charité–Universitätsmedizin Berlin, corporate member of Freie Universität Berlin and Humboldt-Universität zu Berlin, Berlin, Germany; 4 Department of Geriatric Psychiatry, Central Institute of Mental Health, Medical Faculty Mannheim, University of Heidelberg, Mannheim, Germany; 5 Institute of Biometry and Clinical Epidemiology, Charité–Universitätsmedizin Berlin, Berlin, Germany; 6 Berlin Institute of Health, Berlin, Germany; 7 Institute of Social Medicine, Epidemiology and Health Economics, Charité–Universitätsmedizin Berlin, Berlin, Germany; 8 German Center for Neurodegenerative Diseases, Dresden, Germany; 9 Institute of Molecular Biosciences, NAWI Graz, University of Graz, Graz, Austria; 10 BioTechMed Graz, Graz, Austria; 11 Field of Excellence BioHealth, University of Graz, Graz, Austria; 12 HEALTH–Institute for Biomedicine and Health Sciences, Joanneum Research Forschungsgesellschaft mbH, Graz, Austria; 13 Institute for Biology/Genetics, Freie Universität Berlin, Berlin, Germany; 14 German Center for Neurodegenerative Diseases, Greifswald, Germany; 15 Department of Neurology, University Medicine Greifswald, Greifswald, Germany

## Abstract

**Importance:**

Developing interventions against age-related memory decline and for older adults experiencing neurodegenerative disease is one of the greatest challenges of our generation. Spermidine supplementation has shown beneficial effects on brain and cognitive health in animal models, and there has been preliminary evidence of memory improvement in individuals with subjective cognitive decline.

**Objective:**

To determine the effect of longer-term spermidine supplementation on memory performance and biomarkers in this at-risk group.

**Design, Setting, and Participants:**

This 12-month randomized, double-masked, placebo-controlled phase 2b trial (the SmartAge trial) was conducted between January 2017 and May 2020. The study was a monocenter trial carried out at an academic clinical research center in Germany. Eligible individuals were aged 60 to 90 years with subjective cognitive decline who were recruited from health care facilities as well as through advertisements in the general population. Data analysis was conducted between January and March 2021.

**Interventions:**

One hundred participants were randomly assigned (1:1 ratio) to 12 months of dietary supplementation with either a spermidine-rich dietary supplement extracted from wheat germ (0.9 mg spermidine/d) or placebo (microcrystalline cellulose). Eighty-nine participants (89%) successfully completed the trial intervention.

**Main Outcomes and Measures:**

Primary outcome was change in memory performance from baseline to 12-month postintervention assessment (intention-to-treat analysis), operationalized by mnemonic discrimination performance assessed by the Mnemonic Similarity Task. Secondary outcomes included additional neuropsychological, behavioral, and physiological parameters. Safety was assessed in all participants and exploratory per-protocol, as well as subgroup, analyses were performed.

**Results:**

A total of 100 participants (51 in the spermidine group and 49 in the placebo group) were included in the analysis (mean [SD] age, 69 [5] years; 49 female participants [49%]). Over 12 months, no significant changes were observed in mnemonic discrimination performance (between-group difference, −0.03; 95% CI, −0.11 to 0.05; *P* = .47) and secondary outcomes. Exploratory analyses indicated possible beneficial effects of the intervention on inflammation and verbal memory. Adverse events were balanced between groups.

**Conclusions and Relevance:**

In this randomized clinical trial, longer-term spermidine supplementation in participants with subjective cognitive decline did not modify memory and biomarkers compared with placebo. Exploratory analyses indicated possible beneficial effects on verbal memory and inflammation that need to be validated in future studies at higher dosage.

**Trial Registration:**

ClinicalTrials.gov Identifier: NCT03094546

## Key Points

**Question:**

Does 12-month spermidine supplementation have a beneficial impact on memory performance, as well as on other neuropsychological, behavioral, and physiological parameters, in older individuals with subjective cognitive decline when compared with placebo?

**Findings:**

In this randomized clinical trial that included 100 older adults, spermidine supplementation over 12 months did not result in a significant beneficial effect on mnemonic discrimination performance as compared with placebo.

**Meaning:**

Longer-term spermidine supplementation with an increased daily supply of spermidine by about 10% did not modify memory and other biomarkers in a group of older adults at risk for Alzheimer disease.

## Teaser

This randomized clinical trial of older adults in a German clinical research center examines the effectiveness of spermidine as a dietary supplement over 12 months for improving memory and other key measures of cognitive performance compared with placebo.

## Introduction

Maintenance of cognitive function and brain health are of utmost importance in an aging society with an increase in age-related diseases, such as Alzheimer disease (AD). Cognitively healthy older individuals expressing a feeling of persistent cognitive deterioration (ie, subjective cognitive decline [SCD]) are believed to reflect the late preclinical stage of AD, accompanied by aberrant pathological brain changes as well as a higher risk of objective cognitive decline and clinical progression to symptomatic disease stages.^1,2,3,4,5^

Naturally occurring polyamines, particularly spermidine, are essential for multiple cellular processes, including autophagy and the maintenance of cellular homeostasis.^6,7,8,9^ A decrease of polyamine levels is associated with a decline of memory function in aged flies.^9,10,11^ A higher external supply of dietary polyamines restores endogenous spermidine levels in the brain of aging fruit flies, prevents age-related memory impairment,^10,11^ and has been shown to be able to pass the blood-brain barrier in middle-aged animal models.^12,13^ However, corresponding data in humans are still rare, including a pioneer study in healthy middle-aged male adults demonstrating an increase in blood polyamine levels after 2 months of enhanced dietary intake of polyamines.^14^ On that basis, we previously conducted a 3-month randomized, placebo-controlled, double-masked phase 2a study investigating spermidine supplementation in 30 older individuals with SCD. Excellent safety and tolerability as well as preliminary efficacy for improved memory performance in the spermidine-treated group were shown.^15,16^

To further validate the therapeutic potential of dietary spermidine against memory loss in older individuals at risk for AD and to identify possible neurophysiological mechanisms of action, we conducted a 12-month intervention trial in individuals with SCD. We hypothesized that spermidine supplementation (0.9 mg/d) would have a beneficial impact on memory performance, as well as on other neuropsychological, behavioral, and physiological parameters when compared with placebo.

## Methods

### Study Design and Participants

The SmartAge study was a monocenter, randomized, double-masked, placebo-controlled phase 2b trial investigating the effect of 12-month dietary spermidine supplementation (through a spermidine-rich wheat germ extract) on memory performance and biomarkers in older individuals with SCD.^17^ The study was carried out at the NeuroCure Clinical Research Center, Charité–Universitätsmedizin Berlin, and the trial protocol was approved by the Ethics Committee of the Charité–Universitätsmedizin Berlin, Germany (Supplement 1). The study was in accordance with the Declaration of Helsinki and written informed consent was provided by all participants before starting the on-site screening. This study followed the Consolidated Standards of Reporting Trials (CONSORT) reporting guideline for randomized clinical trials.

Older individuals in the age range of 60 to 90 years were recruited from health care facilities and through advertisements in the general population. The main inclusion criterion was a diagnosis of SCD based on established guidelines.^1^ Evaluation of study eligibility was performed during study enrollment, including a telephone screening and an on-site screening. Baseline assessment encompassed neuropsychological testing, questionnaires, and a standardized medical examination. Following baseline assessment, participants were randomly assigned to spermidine-based supplementation (target intervention) or placebo supplementation (control intervention). A computer-based algorithm was used to generate a block-wise randomization sequence stratified by age and sex. Race and ethnicity data were not tracked in this study as there is no evidence that they might affect our outcomes. Participant allocation was carried out on a 1:1 basis by a study collaborator with no involvement in assessing outcomes. Participants in the verum group received a spermidine-rich dietary supplement extracted from wheat germ (The Longevity Labs). The daily dose of administered plant extract was 750 mg (corresponding to approximately 0.9 mg spermidine, 0.5 mg spermine, 0.2 mg putrescine, <0.004 mg cadaverine, and 0.12 mg L-ornithine) administered by 6 capsules of 125 mg each. As a comparator condition, the placebo group received 750 mg of microcrystalline cellulose. Baseline assessments were repeated after the 12-month intervention at a postintervention visit (Supplement 1; eMethods 1 in Supplement 2).

### Outcomes

The primary outcome of this trial was the change in memory performance between baseline and the postintervention visit, operationalized by mnemonic discrimination performance assessed in the Mnemonic Similarity Task (MST).^18^ The mnemonic discrimination index was calculated from the responses during the recognition phase, similar to previous studies.^16,19^ Secondary outcomes included changes in neuropsychological parameters of different cognitive domains (verbal and visual-spatial memory, attention, executive functions, and sensorimotor speed), behavioral parameters of lifestyle, psychoaffective characterization as well as perceived quality of life, and physiological parameters comprising peripheral blood parameters (hematological parameters of safety, inflammation markers/cytokines, markers of vascular injury) and cardiovascular risk factors (vital signs, weight) between baseline and postintervention visit. Safety outcomes included the documentation of adverse events (AEs) and serious adverse events (SAEs) throughout the intervention period (eMethods 2 in Supplement 2).

### Statistical Analyses

Analyses were performed according to the intention-to-treat principle. Missing data were imputed using multivariate imputation by chained equations based on 30 imputed data sets and predictive mean matching generated by the mice package in R version 4.0.3 (R Project for Statistical Computing).^20^ Primary and secondary outcomes were analyzed through covariance models with change in the outcome measure from baseline to 12-month postintervention visit as the dependent variable, intervention group as the factor, and the particular baseline measure (as well as age and sex) as covariates. In a second step, we conducted the same analyses in the prespecified per-protocol set, which included only those participants who successfully completed the 12-month intervention period (total, 89 participants; spermidine group, 42 participants; placebo group, 47 participants). In a third step, we repeated the same analyses (as the sensitivity analyses) for those participants who had successfully completed the 12-month intervention period with compliance rates above 90% (per-protocol plus set: total, 74 participants; spermidine group, 36 participants; placebo group, 38 participants). Safety outcomes (AEs and SAEs) were reported descriptively and analyzed using Poisson regression models, accounting for the different observation periods for each participant and allowing incidence rate and incidence rate ratios to be calculated with confidence intervals (in 100 person-years) in total and by intervention group. A 2-sided significance level of α = .05 was used. There was no correction for multiple testing, and no formal adjustment was made to the *P* values or CIs. The analyses were performed as described in the statistical analysis plan of the trial (eMethods 3 in Supplement 2).

## Results

### Study Participants

**Figure 1.**
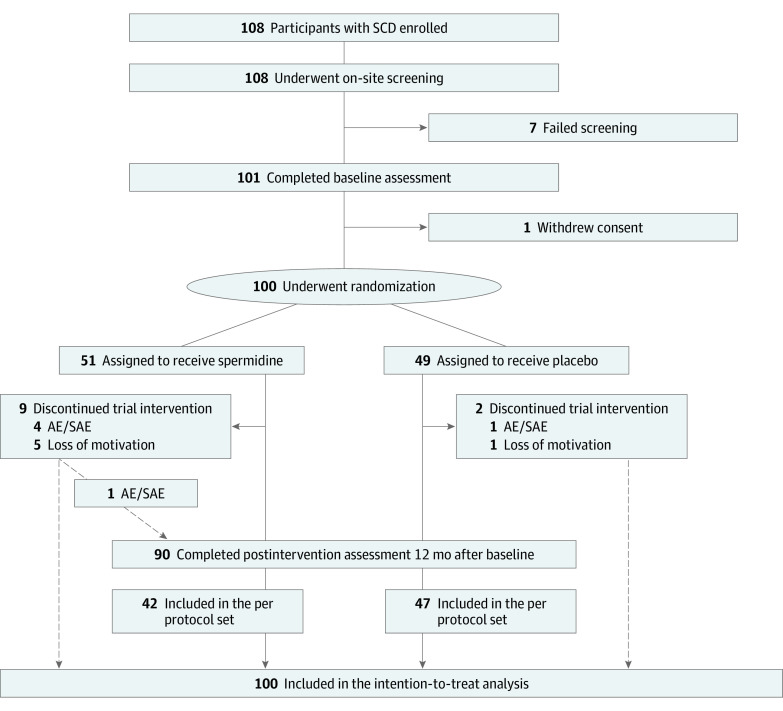
Study Flowchart AE indicates adverse event; SAE, serious adverse event; SCD, subjective cognitive decline. Per-protocol set included all participants completing the 12-month intervention (89 participants). Multiple imputation was performed for intention-to-treat analyses of full analysis set and per-protocol set.

From January 31, 2017, through April 15, 2019, a total of 108 healthy older adults with SCD were enrolled in the SmartAge trial, of whom 100 participants (mean [SD] age, 69 [5] years; 49 women [49%] and 51 men [51%]) were randomly assigned to receive spermidine (51 participants) or placebo (49 participants) and consequently included in the intention-to-treat analysis (Figure 1). At baseline, both groups demonstrated comparable characteristics (Table 1). Follow-up data for 90 participants were available through May 2020. The median (IQR) follow-up period was 369 days (365.5-376.5 days) when considering only the 89 participants who successfully completed the trial intervention. Both groups showed high compliance rates, measured by the number of remaining capsules at postintervention assessment (mean [SD] compliance rate, 95.3% [7.7%]) (eAppendix 1 in Supplement 2).

**Table 1.** Baseline Characteristics of the Participants Included in Intention-to-Treat Analysis

Characteristic	Participants, No. (%)		
	Total (N = 100)	Spermidine (n = 51)	Placebo (n = 49)
Demographics			
Sex			
Women	49 (49.0)	24 (47.1)	25 (51.0)
Men	51 (51.0)	27 (52.9)	24 (49.0)
Age, mean (SD), y	69 (5)	69 (6)	69 (5)
Education, mean (SD), y	17 (3)	17 (4)	16 (3)
Family history			
Dementia	29 (29.0)	13 (25.5)	16 (32.7)
AD	18 (18.0)	9 (17.6)	9 (18.4)
Oldfield hand preference, median (IQR), %	95 (85-100)	95 (90-100)	95 (80-100)
Neuropsychological, mean (SD)			
MMSE, score	29.1 (0.9)	29.1 (1.0)	29.0 (0.9)
LMS delayed recall, score	24.3 (6.3)	24.2 (6.0)	24.5 (6.7)
TMT A, s	39.7 (12.4)	40.4 (12.0)	39.0 (13.0)
MWT, score	32.3 (2.6)	31.9 (3.0)	32.7 (2.0)
Behavioral, mean (SD)			
Psychoaffective characterization			
ECog-39, score	1.8 (0.4)	1.8 (0.5)	1.7 (0.3)
GDS, score	1.9 (1.6)	1.9 (1.7)	1.8 (1.5)
BFI-10, score			
Extraversion	3.2 (1.0)	3.3 (1.0)	3.2 (0.9)
Agreement	3.3 (0.7)	3.3 (0.7)	3.3 (0.7)
Conscientiousness	3.9 (0.8)	4.0 (0.9)	3.9 (0.8)
Neuroticism	3.0 (1.0)	3.1 (1.0)	2.9 (0.9)
Openness	3.7 (1.0)	3.7 (1.0)	3.8 (0.9)
SVF-78, score			
Positive	12.7 (2.6)	12.2 (2.7)	13.3 (2.5)
Negative	10.0 (4.1)	9.4 (4.3)	10.6 (3.9)
Lifestyle			
LEQ, score	99.7 (21.6)	100.0 (23.0)	99.4 (20.4)
CAI, score	3.1 (0.5)	3.0 (0.5)	3.1 (0.4)
Energy intake, kcal/d	2375.4 (747.6)	2471.1 (709.7)	2275.7 (779.8)
MEDAS, score	6.4 (2.3)	6.6 (2.2)	6.3 (2.5)
Alcohol intake, g/d	11.5 (12.04)	10.6 (12.8)	12.3 (11.3)
Physiological			
APOE ɛ4, positive			
Heterozygous	22 (22.0)	7 (13.7)	15 (30.6)
Homozygous	2 (2.0)	2 (3.9)	0 (0.0)
Aβ status^a^			
Positive	7 (7.0)	3 (5.9)	4 (8.2)
Negative	23 (23.0)	12 (23.5)	11 (22.4)
Cardiovascular risk, mean (SD)			
Blood pressure, mm Hg			
Systolic	128.6 (16.2)	126.1 (17.0)	131.2 (15)
Diastolic	80.3 (9.3)	79.8 (10.9)	80.8 (7.4)
Heart rate, bpm	64.7 (9.7)	65.1 (11.3)	64.2 (7.6)
Weight, kg	75.4 (15.3)	76.3 (16.5)	74.4 (14.1)
BMI	26.0 (4.1)	26.3 (4.7)	25.7 (3.4)
Waist circumference, cm	95.5 (11.2)	96.0 (11.4)	94.9 (11.1)
Diabetes	8 (8.0)	5 (9.8)	3 (6.1)
Hypertension	42 (42.0)	23 (45.1)	19 (38.8)
Smoker			
Current	5 (5.0)	4 (7.8)	1 (2.0)
Former	43 (43.0)	19 (37.3)	24 (49.0)

Abbreviations: AD, Alzheimer disease; APOE, apolipoprotein E; Aβ, β-amyloid; BFI-10, Big Five Inventory-10; BMI, body mass index (calculated as weight in kilograms divided by height in meters squared); bpm, beats per minute; CAI, Cognitive Activity Interview; ECog-39, Everyday Cognition Scales; GDS, Geriatric Depression Scale; LEQ, Lifetime Experience Questionnaire; LMS, Logical Memory Scale; MEDAS, Mediterranean Diet Adherence Screener; MMSE, Mini-Mental State Examination; MWT, Multiple-Choice Vocabulary Intelligence Test; TMT, Trail Making Test; SVF-78, Stress Coping Style Questionnaire.

^a^ Cerebral Aβ status was assessed by positron-emission-tomography in only 30 participants.

### Efficacy Outcomes

**Figure 2.**
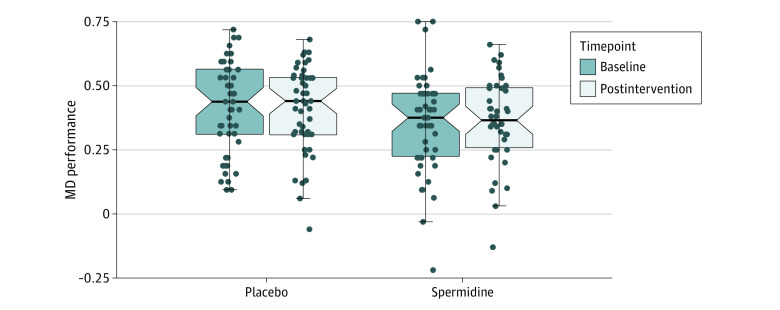
Effect of Spermidine Supplementation on Mnemonic Discrimination (MD) Performance Notched box plots display within-group effects in MD performance in the spermidine group and the placebo group at baseline and postintervention assessments. Box plots include data of all participants who completed postintervention assessment. Notches in the shaded regions indicate 95% CI of the median, and shaded boxes the IQRs with lower (25th) and upper (75th) percentiles. Dots represent individual data points.

**Figure 3.**
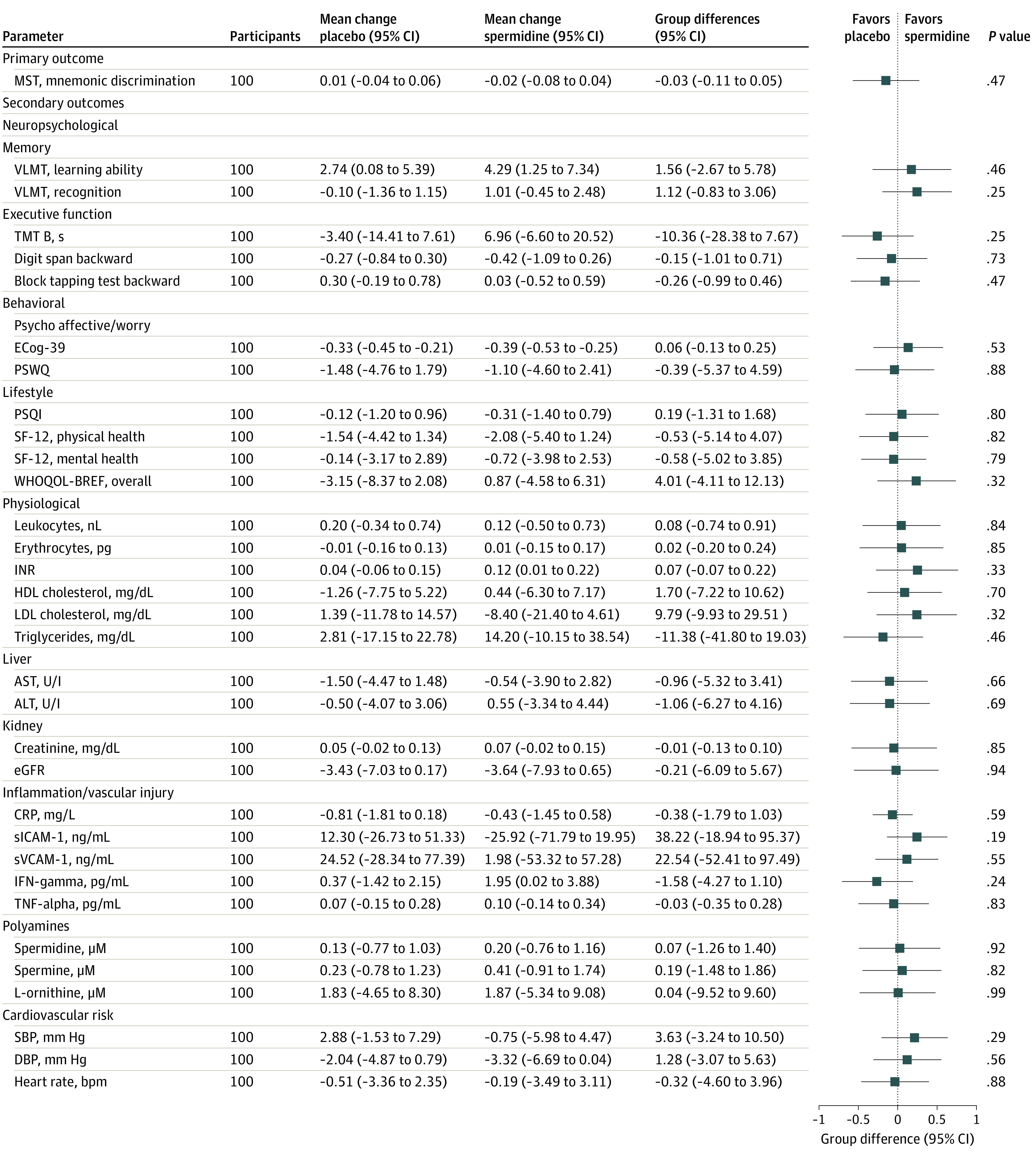
Effect of Spermidine Supplementation on Primary and Selected Secondary Outcomes in Intention-to-Treat Analysis ALT indicates alanine aminotransferase; AST, aspartate aminotransferase; CRP, C-reactive protein; DBP, diastolic blood pressure; ECog-39, Everyday Cognition Scales (39 items); eGFR, estimated glomerular filtration rate; HDL, high-density lipoprotein; IFN-gamma, interferon gamma; INR, international normalized ratio of blood clotting; LDL, low-density lipoprotein; MST, Mnemonic Similarity Task; PSQI, Pittsburgh Sleep Quality Index; PSWQ, Penn State Worry Questionnaire; SBP, systolic blood pressure; SF-12, Short Form Health Survey; sICAM-1, soluble intercellular adhesion molecule-1; sVCAM-1, soluble vascular cell adhesion molecule-1; TMT, Trail Making Test; TNF-alpha, tumor necrosis factor alpha; VLMT, Verbaler Lern-und Merkfähigkeitstest, German version of the Rey Auditory Verbal Learning Test (AVLT); WHOQOL-BREF, World Health Organization Quality of Life. Missing data were imputed using multivariate imputation by chained equations (mice) based on 30 imputed data sets and predictive mean matching. Mean changes of each parameter from baseline to postintervention assessment with 95% CIs are presented for both intervention groups separately. Group differences and *P* values result from analysis of covariance models for change in outcome from baseline to postintervention visit, with the intervention group as the examined factor and adjusted for age, sex, and the particular baseline measure. Mean group differences in the forest plots were standardized by being converted into *z* scores. Forest plots and group differences were transformed, if necessary, to yield the same direction of effect.

Mnemonic discrimination performance was similar in both intervention groups at baseline (eTable 1 in Supplement 2). The adjusted mean change of mnemonic discrimination performance from baseline to postintervention assessment was −0.02 (95% CI, −0.08 to 0.04) in the spermidine group and 0.01 (95% CI, −0.04 to 0.06) in the placebo group, resulting in an adjusted treatment effect of −0.03 (95% CI, −0.11 to 0.05; *P* for primary efficacy outcome = .47) (Figures 2 and 3).

Baseline data of secondary outcomes, including neuropsychological, behavioral, and physiological parameters, were similar for both intervention groups (Table 1; eTable 1 in Supplement 2). Statistical analyses of the full analysis set revealed no substantial treatment effect on any tested secondary parameter (Figure 3; eFigures 1 through 3 in Supplement 2).

Per-protocol analyses revealed no statistically significant intervention effects on mnemonic discrimination performance (−0.04; 95% CI, −0.10 to 0.03; *P* = .25) as well as on all secondary end points (eFigures 4-6 in Supplement 2). This pattern was also found in the per-protocol plus set for the analysis of an intervention effect on mnemonic discrimination performance (−0.04; 95% CI, −0.11 to 0.03; *P* = .28) (eFigure 7 in Supplement 2). However, between-group differences after the 12-month intervention were observed for soluble intercellular adhesion molecule-1 (sICAM-1) and Trail Making Test B (TMT B) (eFigures 7-9 in Supplement 2) in the per-protocol plus set. The adjusted mean change of sICAM-1 concentration in peripheral blood from baseline to 12-month postintervention assessment was −30.5 ng/mL (95% CI, −67.8 to 6.9 ng/mL) in the spermidine group and 25.7 ng/mL (95% CI, −11.2 to 62.7 ng/mL) in the placebo group, resulting in an adjusted intervention effect of −56.2 ng/mL (95% CI, −106.8 to −5.6 ng/mL; *P* = .03), demonstrating a possible beneficial effect of the intervention. The adjusted mean change of TMT B response time was 6.6 seconds (95% CI, −2.2 to 15.4 seconds) in the spermidine group and −7.3 seconds (95% CI, −15.9 to 1.3 seconds) in the placebo group, resulting in an adjusted intervention effect of 13.9 seconds (95% CI, 1.5 to 26.2 seconds; *P* = .03), demonstrating a negative effect of the intervention. No significant intervention effects were observed for any of the other parameters tested.

Exploratory prespecified subgroup analyses in the full analysis set showed no statistically significant differential intervention effects in subgroups. In the per-protocol set and per-protocol plus set, subgroup analyses showed effects of the intervention on memory performance and executive function/processing speed in individuals aged 70 years and older, in men, in individuals with higher baseline dietary spermidine intake, and in individuals with more severe subjective cognitive complaints, respectively (eAppendix 2 in Supplement 2).

### Safety Outcomes

During the 12-month intervention time, 19 SAEs were documented among all participants, 7 in the spermidine group and 12 in the placebo group. All SAEs were rated as not related to the intervention and mild to severe in intensity. The incidence of SAEs regarding fatal or life-threatening events, hospitalization, or malignant and/or neoplastic processes was similar between groups (Table 2).

**Table 2.** Serious Adverse Events During Spermidine or Placebo Supplementation

Characteristic	Participants by group			IRR (95% CI)	*P* value
	Total (n = 100)	Spermidine (n = 51)	Placebo (n = 49)		
Observation time, median (IQR), d	368 (365-376)	367 (364-372)	369 (365-380)	NA	NA
Total SAE					
Participants, No.	19	7	12	NA	.30
IR per 100 PY (95% CI)	19.8 (12.2-30.0)	14.9 (6.4-28.8)	24.4 (13.1-40.9)	0.61 (0.23-1.52)	
Fatal or life-threatening events					
Participants, No.	1	1	0	NA	>.99
IR per 100 PY (95% CI)	1.0 (0.1-4.6)	2.1 (0.1-9.4)	NA	0	
Acute hospital (inpatient hospital treatment or its extension)					
Participants, No.	16	5	11	NA	.17
IR per 100 PY (95% CI)	16.6 (9.8-26.2)	10.7 (3.8-22.9)	22.4 (11.6-38.3)	0.48 (0.15-1.31)	
Malignant/neoplastic processes					
Participants, No.	2	1	1	NA	.97
IR per 100 PY (95% CI)	2.1 (0.3-6.4)	2.1 (0.1-9.4)	2.0 (0.1-8.9)	1.05 (0.04-26.49)	

Abbreviations: IR, incidence rate; IRR, incidence rate ratio; NA, not applicable; PY, person-years; SAE, serious adverse event.

Overall, 129 AEs were recorded (spermidine group, 58 AEs; placebo group, 71 AEs) and the incidence in all system organ classes present in our cohort did not differ substantially between intervention groups (eTable 2 in Supplement 2). Noticeably, musculoskeletal and connective tissue was the only system organ class with substantially more AEs in the spermidine group (11 participants) compared with the placebo group (4 participants). The majority of AEs reported to this system organ class were age-related diseases and symptoms, such as osteoporosis, arthritis, gout attacks, and joint pain. Additionally, 1 participant in the spermidine group reported 2 AEs in that category (eAppendix 3 in Supplement 2).

## Discussion

This phase 2b trial showed no effect following 12 months of spermidine supplementation on memory performance as assessed by mnemonic discrimination performance, as well as on any other neuropsychological, behavioral, or physiological parameter in intention-to-treat analyses compared with placebo. The amount and incidence rate ratios of AEs and SAEs were balanced between groups, and hematologic safety parameters showed no significant changes in either group, indicating excellent safety and tolerability of the spermidine intervention, a result reinforced by high compliance rates.

Consistent with the full analysis set, analyses of the per-protocol and the per-protocol plus set also revealed no effect of intervention on the primary outcome. This finding is not in line with our previous phase 2a study,^16^ where we found an improvement of mnemonic discrimination performance in the spermidine-treated group after 3-month supplementation in the same target group of participants, with a medium effect size. A 2020 study^21^ likewise provided evidence for an improvement of cognitive function after 3 months of higher spermidine intake through nourishment in older individuals with mild to moderate dementia. In addition, studies in aged model organisms also demonstrated improved memory function given a higher external supply of dietary spermidine.^10,12,22,23^ In rodent postmortem samples, polyamine concentration in the hippocampus was associated with age as well as with formation of memory, suggesting that maintenance and restoration of endogenous polyamine levels during aging could benefit memory function, especially in such a sensitive task as the one tested in our primary outcome.^18,19,24,25,26^

The absence of an intervention effect of this clinical phase 2b study on spermidine supplementation might be due to a combination of several factors. First, the daily dose of 0.9 mg spermidine might not have been sufficient to achieve strong effects on memory function and biomarkers in cognitively healthy older individuals. The amount of additional daily spermidine intake via capsules corresponds to about 10% of the average spermidine intake per day in developed countries.^27^ The decision to use a lower range dosage was based on previous reports of higher polyamine levels in some malignant neoplasms and a positive regulatory effect of polyamines on cell growth.^28^ In addition, our preclinical study in mice provided evidence for an increase of relative kidney weight after spermidine supplementation at high dosage.^15^ In our preliminary study, we therefore used a dosage of 750 mg wheat germ extract that was well below the levels at which adverse effects would be observed in mice to assess safety and first efficacy in humans. This dosage was maintained in the present phase 2b trial but might not have been sufficient to induce cognitive benefits. In line with our phase 2a study, blood polyamine levels were also not altered after 12-month intervention. However, blood polyamine levels might not necessarily be an indicator of higher dietary polyamine intake, as polyamines are likely rapidly taken up by solid tissues from blood after absorption from the intestinal lumen.^27,29^ This assumption is supported by a 2021 study^30^ that failed to detect increased spermidine levels in the blood after a 12-month intake of spermidine-enriched natto (a traditional Japanese product), corresponding to an increase of about 14 mg spermidine per day.

Second, individuals with SCD were chosen as a target group to evaluate the target intervention in an early stage of AD, when cognitive function was still preserved, as recommended by landmark publications in the field.^1,31^ However, supplementation with dietary spermidine might not act as a memory booster, but rather prevent age-related memory impairment and development of AD, a possibility supported by evidence from animal studies.^10^ Given that our target group comprised cognitively healthy older individuals with SCD, only subtle cognitive decline was to be expected,^2^ and in fact, we found no significant decline in memory function after 12 months in the placebo-treated group. Thus, to observe a significant difference between groups, a boost in memory performance in the target group would have been necessary, which might not be in the scope of this supplementation regimen. Future trials should include patients with more advanced stages of AD, such as those experiencing mild cognitive impairment, in whom decline in cognitive function over the study period is to be expected.^32^

Apart from a large number of well-described positive effects, there is also evidence of detrimental effects of polyamines, such as potential enhancement of tumor growth, increase of relative kidney weight, or modulation of neuronal excitability.^15,28,33^ In our clinical trial, we demonstrated excellent safety and tolerability of 12-month spermidine supplementation, indicated by comparable amounts and severity of AEs and SAEs without any evidence of newly developed tumors or seizures, inconspicuous kidney parameters, and high compliance to the intervention. However, patients with previously diagnosed malignant neoplasms were excluded, so the impact of spermidine supplementation on tumor growth could not be evaluated in this trial.

Exploratory per-protocol plus analyses provided evidence of a possible beneficial effect of spermidine supplementation on sICAM-1 blood plasma level, a parameter of blood vessel injury and inflammation that is elevated in endothelial dysfunction, inflammatory processes, aging, and dementia.^34,35^ Thus, downregulation of sICAM-1 levels through higher external supply of spermidine might indicate anti-inflammatory and vascular-protective effects, which in turn would contribute to the preservation of higher-order brain functions.^8,36^ Of note, we also observed a slight decrease in TMT B response time, a neuropsychological test assessing executive function and processing speed, which has been shown to be affected by aging and also in SCD.^37,38^ Thus, a decrease in TMT B performance induced by higher spermidine intake might indicate an impairment of white matter microstructure, which in turn is associated with poorer executive function, processing speed, and task-switching.^39,40^ However, other neuropsychological tests in this domain do not support this finding, nor does the positive evidence for anti-inflammatory effects of spermidine support a detrimental effect on white matter integrity, so this finding needs to be validated or refuted in future trials. Subgroup analyses in the per-protocol and per-protocol plus sets provided some indication for a possible beneficial effect of spermidine supplementation on memory performance in individuals aged 70 years and older, in men, and in individuals with more severe subjective cognitive concerns, respectively. However, all exploratory findings would not be statistically significant after correction for multiple testing. Thus, these exploratory analyses need to be interpreted with caution but might serve as starting points or useful for generating hypotheses to be tested in future intervention trials.

### Limitations

Several limitations should be considered when interpreting our findings. First, biomarkers for AD (amyloid, tau, phosphorylated tau) were not required for study participation, and cerebral amyloid-β status was available from only 30% of participants. Thus, negative findings in this trial might be due to a potentially large percentage of individuals without amyloid or tau pathology, indicating other reasons for worrying about their cognitive function that might be less susceptible to dietary approaches.^2,6^ However, our participants with SCD were characterized according to currently accepted guidelines for SCD,^1^ and our intervention did not specifically target amyloid or tau. Second, we chose an intervention period of 12 months, which might have been too short to observe significant changes in cognition and biomarkers. However, other supplement trials were similar in length, and an intervention of only 3 months had previously shown beneficial effects.^16,41^ Nevertheless, longer intervention periods might induce stronger effects and might allow for assessing effects of spermidine supplementation on naturally occurring cognitive decline in older adults.

## Conclusions

Supplementation with spermidine that increased daily supply by about 10% did not result in a beneficial effect on memory function or other neuropsychological, behavioral, or physiological parameters. Positive results from per-protocol and subgroup analyses regarding memory function and anti-inflammatory actions, combined with the excellent safety profile of the supplementation, endorse future trials with higher dosage to further investigate spermidine as a mean to delay cognitive decline in individuals at risk for AD.

## Notes

Supplement 1.
Trial Protocol and Statistical Analysis Plan

Supplement 2. **eMethods 1.** Study Design and Participants **eMethods 2.** Outcomes **eMethods 3.** Statistical Analyses **eAppendix 1.** Trial Participants and Compliance **eAppendix 2.** Subgroup Analyses **eAppendix 3.** Adverse Events **eTable 1.** Baseline Assessment of Primary and Secondary Outcomes **eTable 2.** Adverse Events During Spermidine and Placebo Supplementation **eFigure 1.** Effect of Spermidine Supplementation on Selected Neuropsychological Parameters—Intention-to-Treat Analysis **eFigure 2.** Effect of Spermidine Supplementation on Selected Behavioral Parameters—Intention-to-Treat Analysis **eFigure 3.** Effect of Spermidine Supplementation on Selected Physiological Parameters—Intention-to-Treat Analysis **eFigure 4.** Effect of Spermidine Supplementation on Neuropsychological Parameters—Per Protocol Analysis **eFigure 5.** Effect of Spermidine Supplementation on Behavioral Parameters—Per Protocol Analysis **eFigure 6.** Effect of Spermidine Supplementation on Physiological Parameters—Per Protocol Analysis **eFigure 7.** Effect of Spermidine Supplementation on Neuropsychological Parameters—Per Protocol Plus Analysis **eFigure 8.** Effect of Spermidine Supplementation on Behavioral Parameters—Per Protocol Plus Analysis **eFigure 9.** Effect of Spermidine Supplementation on Physiological Parameters—Per Protocol Plus Analysis
eReferences.

Supplement 3.
Data Sharing Statement
