# Supplementary material for: Effects of Spermidine Supplementation on Cognition and Biomarkers in Older Adults With Subjective Cognitive Decline: A Randomized Clinical Trial
Source: JAMA Netw Open. 2022 May 26;5(5):e2213875. doi: 10.1001/jamanetworkopen.2022.13875 (PMC9136623; doi:10.1001/jamanetworkopen.2022.13875)
Supplement: Supplement 1. — Trial Protocol and Statistical Analysis Plan [file jamanetwopen-e2213875-s001.pdf]

Schwarz C, Benson GS, Horn N, et al. Effects of spermidine supplementation on cognition and biomarkers in older adults with subjective cognitive decline: a randomized clinical trial. *JAMA Netw Open*. 2022;5(5):e2213875. doi:10.1001/jamanetworkopen.2022.13875

Trial Protocol

Request for advice from the ethics committee on the implementation of a medical-scientific project that does not involve the clinical testing of a medicinal product

1. Title of the study	Investigation of the effect of polyamine-rich dietary supplementation on cognitive performance and other biomarkers in elderly subjects with subjectively perceived cognitive deterioration (SmartAge)<br><br>- a randomized, monocentric, placebo-controlled, double-blind study
2. Ethics committee application number	EA1/250/16
3. Decisions of other ethics committees on the same issue	The study project was already evaluated by the Charité Ethics Committee in 2014 and initially rejected (EA1/347/14). We followed the recommendation and conducted a shorter preliminary study over 3 months to test the potential positive effect of polyamines on cognition. These results were incorporated into the present application and provide the basis the basis for a study with a longer intervention duration and a larger number of study participants.
4. Subject of the study and its objectives; stating the hypotheses, separated into main and secondary hypotheses, as well as the clinical parameters (primary and secondary endpoints), which will be used to test the hypotheses	**Subject**:<br>Animal studies have shown that polyamine-rich diets can help to stop age-related memory loss in fruit flies[1]. Endogenous polyamines are important products of cellular metabolism whose concentration decreases with age in both flies and humans[2,3]. At the same time, the accumulation of proteins such as beta-amyloid (Aß) in the brain increases, a process that is discussed as a major contributor to Alzheimer's disease[4]. Polyamines trigger a cellular process (autophagy), which removes cellular "waste" (including protein aggregates) and thus may contribute to the maintenance of cognitive health[5, 6]. However, to date, it is largely unclear whether the memory-enhancing effect of polyamines also operates in humans and to what extent this effect can be explained by the influence of polyamines on various biomarkers.<br><br>In a placebo-controlled proof-of-concept study of our own research group (EA1/233/15; further referred to as preliminary study) with elderly people with subjective cognitive decline (SCD; further referred to as SCD patients), it could be shown that polyamine supplementation compared to placebo (14 vs. 14 SCD patients) for 3 months [1] improved memory performance (trend). Furthermore, it could be shown that [2] capsules were taken regularly and [3] that they were very well tolerated.<br><br>The aim of the proposed randomized, monocentric, placebo-controlled, double-blind study is to evaluate the effect of polyamine supplementation (identical daily dosage per day as in the preliminary study) in a **larger cohort** of patients with SCD (SCD cohort, 50 patients with polyamine intervention vs. 50 patients with placebo intervention) over a **longer period of time (12 months)** on sensitive cognitive domains identified in the preliminary study (**memory function, primary target**); as well as on a number of potentially **underlying mechanisms (secondary targets)** to be investigated. For this purpose, various biomarkers (including markers in blood and muscle tissue, perfusion markers, and functional and structural markers of gray matter) will be investigated. As this is, to the best of our knowledge, the first long-term polyamine intervention study, it is important to establish in a comparison to what extent one biomarker or the combination of biomarkers explains the effect of polyamine supplementation on cognitive performance (further referred to as multimodal analysis).

Primary Objective:
The primary objective is to investigate whether a targeted polyamine-rich dietary supplementation (polyamine supplementation) compared to the control condition (placebo) leads to a change in memory performance in the SCD cohort.

Primary hypothesis:
The 12-month intervention (polyamine-rich dietary supplementation) will lead to improved memory performance in the SCD cohort compared with the control condition (placebo), as operationalized by a Pattern-Separation-Task.

Secondary Objectives:
Furthermore, the above study aims to investigate whether a polyamine-rich dietary supplementation leads to positive effects in the cognitive performance of other domains in addition to the positive effect on memory performance in the SCD cohort compared to the control condition (placebo), which mechanisms underlie these effects, which moderators influence the response to the intervention, and whether a long-term effect can be demonstrated. In addition, a multimodal analysis will be performed to investigate the influence of the polyamine intervention on different biomarkers [in blood and structural, functional and perfusion MRI (multimodal analysis)], as well as their association with cognitive performance will be comparatively investigated. This intended scale-independent comparability of biomarkers and their correction for normal age- and sex-typical changes are achieved using age- and sex-corrected z-transformations based on an independent healthy reference cohort (as also established in previous work [7, 8]). The model was developed with the Biostatistician Dr. U. Grittner.

Secondary hypotheses:
In the SCD cohort, polyamine-rich supplementation will lead to the following changes compared to the control condition (placebo):

Performance in other cognitive domains:

1) In addition to improved memory performance (primary hypothesis), intervention over a 12-month period will lead to improved executive function and attention (measured by validated neuropsychological tests, details under point 10).

Autophagy processes:

2) Furthermore, there will be improved autophagy in the body (measured by metabolite status in muscle tissue) and also associated improved muscle function and mass (as determined by sarcopenia testing and Bioelectrical Impendance Analysis (BIA)).

Imaging:

3) In addition, there will be better structural/functional neuronal plasticity (measured through MRI for gray/white matter structure, functional activation, and functional connectivity) and better cerebral vascular processes (including perfusion measurement using sensitive MRI sequence: arterial spin labeling).

Blood parameters:

4) An increase in the concentration of polyamines and neurotrophic factors will be observed, as well as a decrease in inflammatory factors and markers of oxidative stress in blood plasma.

	Peripheral biomarkers:<br>5) In addition, peripheral vascular processes will improve, including arterial vascular stiffness (pulse wave velocity and augmentation index measured using an arteriograph).<br><br>*<u>Moderators of response to the intervention</u>*<br>Interindividual differences in response to the intervention (polyamine supplementation vs. placebo) could be in part...<br><br>6) ... explained by different expressions in genes among study participants in terms of different genetic polymorphisms (e.g., brain-derived neurotrophic factor (BDNF), apolipoprotein E (APOE), catechol-O-methyltransferase (COMT), TOMM40), which are responsible for energy metabolism and memory performance.<br><br>7) ... explained by initial Aß deposition in the brain (more Aß deposition allows for a greater intervention effect than less Aß deposition).<br><br>*<u>Assessment of the long-term effect of the intervention</u>*<br>8) Compared to placebo intervention, even 6 months after completion of the polyamine intervention, the increased polyamine concentration in the blood and better cognitive performance persists.<br><br>*<u>Multimodal analysis of the intervention effects</u>*<br>9) The most sensitive biomarker or a combination of several biomarkers, which mediates the relationship between polyamine intervention and better cognitive performance compared to placebo intervention the most or explains the association, can be identified using data collected from a healthy reference cohort.<br><br>**Primary endpoint**<br>- to determine and compare test scores (see Primary Hypothesis above) immediately before and after the end of dietary supplementation with polyamines or placebo (follow-up after 12 months (T2, post-intervention) versus baseline (T1, pre-intervention)).<br><br>**Secondary endpoints**<br>- determination and comparison of test scores or markers (see above points 1-5) immediately before and after the end of the dietary supplementation with polyamines or placebo (follow-up after 12 months (T2, post-intervention) versus baseline (T1, pre-intervention)) with inclusion of markers from items 6 and 7 above.<br><br>- follow-up 6 months after post-intervention (equivalent to 18 months after baseline, T3). Determination and comparison of test scores or markers (points 1, 4, 5 above) in comparison to previous measurement points: T1 and T2 (see above point 8).<br><br><u>Note:</u> A follow-up examination is planned after 6 months (no endpoint). During this visit, compliance will be assessed (drug accountability and interview on intervention) and polyamine or placebo capsules will be dispensed. Furthermore, a telephone call after 3 and 9 months is planned to verify compliance (interview on intervention).

5. Explanation of the significance of the study	The increase in the number of older people in our society is expected to result in a 2-4-fold increase in people who develop dementia[9]. Therefore, the search for prevention and intervention strategies to prevent, or at least delay, cognitive deterioration and neurodegeneration is of high relevance in terms of health policy, economics, and medicine.<br><br>Older people with SCD and related concerns show an increased risk of developing Alzheimer's dementia compared to people without SCD[10], even if no objective deterioration can be detected in neuropsychological tests. SCD is considered a very early prodromal stage of Alzheimer's disease, in which pathological changes already occur[11]. The first complaint of memory problems is associated, for example, with increased Aß deposition[12], gray matter volume reduction[13, 14], hypometabolism[15], and functional changes[16] in brain regions typical for Alzheimer's disease. In this patient group, it is therefore of particular interest to slow down early brain pathological processes and, thus, preserve cognitive functions as long as possible.<br><br>Animal studies have shown that polyamine-rich diets can help to stop age-related memory loss in fruit flies.<br><br>Therefore, polyamine-rich dietary supplementation could be a potential intervention against age-related memory decline, especially in patients with SCD. First indications of this could already be observed in a preliminary study with a 3-month polyamine intervention in elderly patients with SCD. Here, a trend towards improved memory function was shown as a result of supplementation with polyamines.<br><br>It is therefore hypothesized that the proposed study, with a larger patient sample and longer intervention duration, will provide significant insights into the impact of polyamine-rich dietary supplementation on changes in cognitive abilities in SCD patients. This could be a significant step in the long-term development of interventional methods for the (early) therapy of dementia and the underlying pathological processes.<br><br>Furthermore, the study may provide insights into potential moderators of the intervention effects (such as Aß status in the brain and genetic manifestations) and may demonstrate the effect of polyamines on autophagy, muscle function, peripheral blood parameters, and brain structure and function.<br><br>*Rationale for studying a healthy reference cohort:*<br>In order to understand the underlying mechanisms of a possible protective effect of the polyamine intervention on cognitive performance in SCD patients, and thus to enable further development and/or refinement of such procedures, it is essential to comparatively examine different biomarkers for their mediating effect (by using a so-called multimodal analysis).<br><br>Regarding this, it is important to note that sensitive biomarkers of Alzheimer's disease are also influenced by normal aging processes and a person's sex[17,18]. However, it is possible to determine the extent of these associations using a healthy reference cohort, and to adjust the values of the SCD cohort accordingly. This has been shown in previous studies[7, 8]. For this purpose, the raw biomarker data from the SCD cohort (measured at T1 and T2) will be z-transformed using the independently collected biomarker data of the healthy reference cohort (measured at T1) and corrected for normal age- and sex-typical changes, also determined on the basis of the reference cohort[8, 19]. These z values describe for each biomarker the degree of deviation from the expected

	values in the healthy reference cohort and allow the identification of the most sensitive biomarkers with respect to the potential positive effect of a polyamine intervention on cognitive performance of SCD patients. For this purpose, it is important to examine the reference cohort using the same measurement methods and devices as the SCD cohort.
6. Which of the following regulations apply<br><br>*a) Medical Devices Act*<br>*-according to § 20 MPG (device does not have the declaration of conformity or this is and a different indication is being tested or additional invasive or other stressful examinations are being performed)* ***or***<br>*-according to § 23 MPG ?*<br>*b) Radiation Protection Ordinance § 23*<br>*c) X-ray ordinance § 28 a*<br>*d) Genetic Engineering Law*<br>*e) Data protection laws*	b) Radiation Protection Ordinance<br><br>e) Data protection laws<br>The collection and archiving of data follows the Berlin Data Protection Act - BlnDSG and the European Data Protection Regulation – DSGVO
7. If applicable: designation and characterization of the test products (e.g., devices for MPG studies; please include attachments)	Not applicable
8. Substantial results of the preclinical tests or reasons for not performing them	**Relevant studies on polyamines in general**<br>Endogenous polyamines (i.e., putrescine, spermidine, and spermine) contribute to the maintenance of cellular processes (cell growth, cell division and cell survival[2]), the regulation of neurotransmitter processes, and the modulation of learning and memory processes[20]. It is assumed that polyamines are substances that can mimic the positive effects of fasting/calorie restriction without having to follow a diet.<br><br>**Relevant studies on polyamines, memory and aging in animal models**<br>In many organisms, intracellular polyamine concentrations decrease with aging in a number of organs, including the brain[2]. In fruit flies, it has been shown that the age-related reduction of polyamine concentrations (especially spermidine and putrescine) in the brain is associated with reduced memory performance. However, external polyamine supplementation (e.g., spermidine) can increase endogenous polyamine concentration[1, 21], increase the life span in yeasts, fruit flies, as well as worms, and induce autophagy in these model organisms[6] and in mice[5]. Polyamine-rich diets can also stop the age-related memory loss in fruit flies, which simultaneously correlates with the increase in autophagy processes[1]. This cellular self-cleaning process can reduce cellular "junk," including protein aggregates, e.g., of tau protein[22] in mouse models, and thus may help to prevent neurodegenerative processes. Soda and colleagues were able to show that mice which were fed with polyamine-rich diets lived longer and had fewer age-related pathologies[23]. Furthermore, hippocampal polyamine concentrations (spermidine, spermine) correlated with memory formation in this animal model[20].<br><br>**Impact of polyamines on disease processes and neuronal excitation in animal and human models**<br>In mammals, polyamines are involved in many growth and

	differentiation processes of tissues as well as in remodeling processes[24]. However, polyamines are also the subject of cancer research, as a correlation between increased polyamine synthesis, cell growth and cancer has been shown[25]. However, an increase in oncogenic alterations by polyamine-rich supplementation has not been observed in either healthy mice or humans[21]. It is suggested that the increase of putrescine concentrations in the cell leads to increased neoplasia[26, 27]. Another study in mice has shown that spermine plays an important role in inhibiting pathological changes (such as tumorigenesis), while it can accelerate the growth of existing tumors[28].<br><br>Polyamines also influence neuronal excitability in the brain through modulation of ion channels and receptors[29], e.g., NMDA-type glutamate receptors[30] and inwardly rectifying potassium channels (Kir channels)[31]. Hence, polyamines modulate the generation of excitation in neurons. Concomitantly, dysregulation of polyamine metabolism has been associated with several diseases[32], such as epilepsy[33], mental disorders[34], stroke[35] and diabetes mellitus[36, 37].<br><br>The effect of polyamines in these processes is highly complex and may involve both neuroprotective and neurotoxic mechanisms. However, in a previous study with polyamine-rich diet, no such side effects were observed in healthy mice or humans[21]. In addition, age-related dysregulation of polyamine metabolism is suggested to be partly underlying the various pathologies and morbidities that can become established in old age. A study by Pucciarelli and colleagues[38] has shown that spermidine and spermine concentrations in relation to total polyamine concentration are elevated in the blood of healthy ninety- to one hundred-year-old individuals. Therefore, it is hypothesized that the maintenance of spermidine and spermine concentrations and the decrease in systemic putrescine concentration in the blood may be important for healthy aging.
9. Essential content and results of previous studies/applications of the products to be tested in the study	**Polyamine concentration in food**<br>Polyamines, such as spermine and spermidine, are present in almost all plants and animal products and have important physiological functions. . Based on previous studies, the fermented soybean product "Natto", for example, contains approx. 50-120 mg/kg polyamines or 80- 100 mg/kg spermidine and spermine. For example, Okamoto and colleagues report 116 mg/kg of the polyamines, spermidine and spermine, in natto[39]. Another study by Zhang and colleagues (2007, online reference: http://en.cnki.com.cn/Article_en/CJFD TOTALSHNX200701001.htm) detects spermidine, spermine, and Putrescine in commercial "natto." The authors report the total concentration of biogenic amines in two types of "natto" as 80-100 mg/kg. The results of Zhang and colleagues show that 95% of the biogenic amines in "Natto" are polyamines, which are not acutely toxic to humans, including 70% spermidine. A third study by Ibe and colleagues[40] measured a spermidine concentration of 53 mg/kg. Slight discrepancies in the data on polyamine content may be due to the fact that "Natto" is a fermentation product. The composition of the final product depends, among other things, on the bacterial strains used, the conditions under which the fermentation took place and which starting material was used.<br><br>To date, there is only a limited understanding of the uptake of polyamines from major dietary sources. Some studies have analyzed the content of polyamines in foods[39, 41] or estimated the mean intake of polyamines in individual countries such as Japan 200 µmol, mainly putrescine,[42] and the United Kingdom 350-500 µmol[43]. According to estimates in various countries, including the United Kingdom, Italy, Spain, Finland, Sweden, and the Netherlands[44], the mean polyamine

intake for adults is: approximately 212 µmol/day putrescine, 87 µmol/day spermidine, and 55 µmol/day spermine. The average polyamine intake through food is thus below the guideline of the Swedish nutrition recommendations objectified (SNO), which recommend an average polyamine intake of 541 µmol/day[45].

In the study by Soda and colleagues[21], the average intake of "Natto" is 66.4 ± 3.7 g/day. This corresponds to a daily polyamine intake of approx. 54 µmol/day (data of the authors) and therefore amounts to about one tenth of the SNO guidelines.

Extraction of polyamines from wheat germs
The collaborating partner of the preliminary study and this study (Prof. Dr. Frank Madeo, Institut für Molekulare Biowissenschaften, Graz, Österreich) has developed a method of polyamine extraction that enables the production of dietary polyamine-rich plant extracts to produce health-promoting food supplements. This extraction method manages to produce polyamine-rich plant extracts without strong acids, organic solvents and/or the use of harmful chemicals harmful to health or the environment to extract polyamines from plants. The equipment and techniques used in this process have already been used to produce food and/or various pharmaceuticals. Thus, their application in the production of food products intended for use on animals and humans is safe and harmless. Wheat germs are used as the starting material for the extraction, as they contain a very high concentration of polyamines (especially spermidine) and are used in human nutrition for centuries. The efficiency of the extraction was further optimized in the laboratory under the direction of Prof. Dr. Frank Madeo.

In the extract to be administered, mainly spermidine and spermine are enriched (with 1.2 mg spermidine and 0.6 mg spermine per 1 g of extract). Furthermore, 1 g of extract contains: 0.2 mg Putrescine, <0.005 mg Cadaverine, and 0.166 mg L-ornithine. In combination with a normal diet, the intake of the planned daily dose of 750 mg of extract (even in the case of multiple overdoses) is below the calculated NOAEL (No-Observed-Adverse-Effects Level) for humans of 29 mg/kg body weight (bw)/day for cadaverine and putrescine[46]. This is also the case for spermidine and spermine, for which the NOAEL is 13.5 mg/kg bw/day and 3.1 mg/kg bw/day, respectively[46]. The NOAEL for L-ornithine in humans is above 500 mg/kg bw/day[47] and thus remains unattainable with the planned administration of the extract.

Within the scope of this study, the wheat germ extract is administered daily in the form of a maximum of six capsules, each containing 125 mg of extract, thus resulting in a daily dosage of 750 mg extract or 0.9 mg spermidine, 0.5 mg spermine, 0.2 mg Putrescine, <0.004 mg Cadaverine, and 0.12 mg L-ornithine. This dosage is thus below the administered amount of polyamines in the earlier substitution study from Japan[21]. The study by Soda and colleagues administered approximately 10 mg/day of spermidine (maximum value) in humans. This amount of polyamines would also be conceivable with a normal and targeted diet with, e.g., 200 g of cooked soybeans,[45] and should accordingly lead to no side effects.

To verify safety of the extracts for animal and human use, chemical food analysis was performed on the extracts. Except for didecyldimethylammonium chloride (DDAC) and benzalkonium chloride (BAC), no traces of toxins and pesticides were detected in the extracts. The measured concentration was 2.7 mg/kg extract for DDAC and 1.2 mg/kg extract for BAC (total). At a dosage of 750 mg extract per day, the EFSA (European Food Safety Authority) is not reached by these two substances. The daily dosage for DDAC is 0.029 µg/kg bw[1] and for

	BAC (total) is 0.013 µg/kg bw[2] at an intake of 750 mg extract[3]. Thus, the ADI (Acceptable Daily Intake) of 100 µg/kg bw/day for these two substances is not exceeded in an adult.<br><br>Additional ingredients that were found to be above the detection limit in the extracts were various B vitamins and cadmium (Cd, 0.15 mg/kg extract). With the dosage of 750 mg extract per day, a weekly amount of 0.0016 µg Cd/kg bw/day is supplied and is thus far below the prescribed 0.36 µg Cd/kg bw/day[4]. In the case of the B vitamins, the measured amount is too low to have an effect on health at the planned dosage. Other components of the extract include plant proteins and sugars, but these are not expected to have any effect on human health. However, the extracts are not suitable for use in people with gluten intolerance.<br><br>**Tolerability studies on polyamine supplementation in mouse model**<br>A 28-day tolerance study on mice (performed according to the OECD guideline for studies of chemical substances "Test 407: Repeated Dose 28-day Oral Toxicity Study in Rodents") showed no changes in eating, drinking or general behavior as a result of the treatment (qualitative evaluation). The following amounts of the extract were administered daily via the feed to mice: 0.56 g/kg bw/day (low), 5.6 g/kg bw/day (medium) and bw/day (medium), and 56 g/kg bw/day (high). These correspond to 10 mg spermidine/kg bw/day, 100 mg/kg bw/day, resp. 1000 mg spermidine/kg bw/day. Organ weights of all examined organs except spleen and kidneys remained unchanged by treatment. The kidney weight (relative to bw) was increased by 9.9% at the high dose in both sexes (p-value: 0.005). Based on this measurement, it is recommended that individuals be screened for kidney disease and, if necessary, to exclude them from the study after assessment by the physician. Spleen weight (relative to bw) was decreased by 18% only in males at the highest dose (p-value: 0.004). Among females, the spleen weight (absolute and relative to bw) remained unchanged. The incidence of cardiac fibrosis was reduced by 80% percent by treatment at the low and medium doses. At the highest applied concentration, no cases of cardiac fibrosis were identified. Since the dosage used in humans is a factor of 100 lower than the low concentration used in animal studies, we do not expect any negative effects in terms of supplementation of the extracts on humans.<br><br>In the literature, inhibition of Kir channels is suggested by cationic properties of polyamines (especially spermine)[48]. This could even explain the different dynamics of uptake of potassium ions by Kir channels in different tissues[49]. Despite many in vitro studies on this topic, few animal models have been developed to investigate this property of polyamines and their physiological relevance in vivo[50]. Most importantly, no positive study results on such a modulation during oral administration of polyamines have been published[48]. Despite the lack of information, these effects cannot be ruled out with adequately dosed supplementation. They could even explain the observed beneficial effects on myocardial function with lifelong oral supplementation (Madeo, unpublished data).<br><br>In the context of this study, polyamine amounts are used that can be easily reached and even exceeded in a balanced diet. The daily dose of 11.15 µmol spermidine and spermine in total per person can only with difficulty compensate for a concentration increase from 5 µM to 20 µM spermine in the intracellular space[49] known from literature. Therefore, we estimate the risk for modulation of Kir channels by extract administration to be low, being comparable to a vegetarian diet rich in soy products.

Relevant studies on polyamines in humans
In humans, an age-related reduction in endogenous polyamine concentration has been reported in basal ganglia[51] and in cortical regions[3] in post-mortem studies. A two-month polyamine-rich diet containing "Natto" (on average 66 g/day, maximum 100 g/day), Soda and colleagues were able to increase the endogenous polyamine concentration in humans by a factor of 1.39 (esp. spermine) in blood plasma[21]. This pioneering study simultaneously demonstrated a good tolerance and acceptance of the nutritional intervention among study participants.

<u>Results of the preliminary study (EA1/233/15)</u>

In the just completed preliminary study, with 14 patients with SCD (age range 60-80 years) who received a polyamine-rich dietary supplement extracted from wheat germs (approx. 0.9 mg/day spermidine, 0.5 mg/day spermine) for 3 months (vs. 14 SCD patients receiving placebo), we evaluated the safety of the intervention and the effect of the supplementation on cognitive performance were confirmed, and very good compliance was demonstrated.

<u>Safety/ Tolerability</u>
Of the total 33 SCD patients recruited in the preliminary study, 3 participants did not meet the screening criteria and 2 participants terminated the intervention prematurely due to lack of motivation and time constraints, leaving a total of 14 participants who completed the polyamine and 14 participants who completed the placebo intervention after 3 months. There were 2 events (one per group) that were classified as adverse events (AE; serious adverse event, SAE) but were not associated with capsule intake from a physician's point of view. This involved, on the one hand, an acute bronchitis with pneumonia developing from it (with a short stay in the hospital). This participant received placebo. Furthermore, two days before the end of the 3-month intervention with polyamines, one participant suffered a mild allergic reaction, which was accompanied by a short hospital visit (emergency room). The definite cause could not be determined. A possible explanation would be an allergic reaction to oak processionary moths, since the participant had been in a corresponding risk area shortly before and the symptoms were fitting. From a medical point of view, there was no correlation with the ingestion of the polyamine capsules. The participant had completely recovered after 2-3 days.

Capsule intake and tolerability of the capsules were otherwise consistently described as good by the participants, with only 4 participants of the placebo group reporting the following side effects: Weight gains (n=2), stimulated digestion, slightly more diarrhea and slight gastric distress at the beginning, which disappeared after a few days (n=2).

Furthermore, with respect to potential polyamine-associated disease-causing processes no changes were observed in the blood count (e.g., altered leukocyte count) that would indicate some form of organ dysfunction or oncogenesis.

<u>Compliance</u>
To determine capsule compliance over the 3 months, the patients were asked about the regularity of their capsule intake at the end of the study and the number of remaining capsules was counted.

In a questionnaire, study participants reported that they had forgotten no more than 5 capsules on average (out of a planned 252 capsules) during

	the entire intervention period of 3 months. After counting the remaining capsules per study participant, it also showed a very good compliance rate of 98.01% (98.83% in the polyamine group, 97.17% in the placebo group).<br><br><u>Cognitive performance</u><br>At baseline, both intervention groups (polyamine vs. placebo intervention) did not differ in age, sex, and education, nor in blood pressure, body mass index, and smoking behavior (all p's <0.05).<br><br>It could be shown that polyamine intake for 3 months already has first positive effects on the memory performance of patients with SCD:<br><br>In the so-called pattern separation test, the study participant was first shown 32 pictures of everyday objects on the computer, referred to as the encoding phase. Participants were asked to decide whether these items could be found "indoors" or "outdoors," with no "correct" or "incorrect" rating. This was followed immediately by a recognition phase in which these pictures were shown again (n=32), mixed with similar-looking (lures, n=32) or completely new objects (novel foils, n=32). Here, participants were asked to name whether these pictures were "old," "new," or "similar" to the pictures in the encoding phase. As a sensitive measure of memory performance, a so-called BPS score (Behavioral Pattern Separation Score) was calculated as follows: correct naming of similar items (lures) with "similar" minus incorrect naming of novel items (foils) with "similar".<br><br>After a 3-month polyamine intervention, there was a tendency for greater improvement in BPD scores (n=14, baseline MW=0.18 ± SD=0.13, follow-up MW=0.26 ± SD=0.19, Cohen's d=0.49) compared with placebo intervention (n=13, baseline MW=0.17 ± SD=0.17, follow-up MW=0.20 ± SD=0.15, Cohen's d=0.19). The effect size Cohen's d of the group difference was 0.5, after correction for age, sex, and education (ANCOVA). Based on these data, the analysis described in point 15 " PROPOSED SAMPLE SIZE/POWER CALCULATION " listed case number estimation of this study was performed.<br><br>*<u>Significance of an examination of muscle tissue, muscle function and muscle mass</u>*<br><br>It is assumed that polyamines have an influence on cellular metabolism and thus on the vitality of the organism by, among other things, modulating autophagy processes[6, 52]. Preliminary results in mice show that chronic ingestion of spermidine leads to improved mitochondrial function and maintenance of morphology in (cardiac) muscle tissue[53]. However, no robust method exists to measure the systemic autophagy rate in blood. So far, a statement can only be made by analyzing metabolically more active tissues such as brain or muscle. Since the sampling of brain tissue is not possible, a muscle biopsy should help to look at intervention-associated autophagy processes and to detect therapeutic targets.<br><br>In the context of the polyamine intervention study presented here, proteins involved in the assembly of autophagolysosomes as the starting point of cellular macroautophagy will be investigated by means of muscle biopsies (via immunohistochemistry)[54] and the posttranslational regulation of cellular autophagy capacity in dependence on the intervention will be examined (via acetylproteomics)[55].<br><br>The sampling of muscle tissue offers a unique opportunity to investigate the effect of polyamines on autophagy processes directly in the tissue,

i.e., at the "reaction site", and thus allows indirect conclusions to be drawn as to the extent to which polyamines can counteract cognitive decline in the brain by means of such cell purification processes.

In addition to cognitive impairments, muscle wasting (age-related disease: sarcopenia) in old age is one of the main factors affecting the quality of human life[56]. In the mouse model it could be shown that the administration of polyamines leads not only to morphological improvements but also to functional improvements (function of the mitochondria)[53]. Using short and non-invasive tests (for details see point 10) the degree of age-associated sarcopenia phenomena can also be easily determined in humans. The aim is to investigate whether the administration of polyamines can improve muscle function and thus increase the patients' sense of well-being. Already a reduction of the risk or a slowing down of such a disease would represent a valuable result.

Possible moderators of response to intervention:
Genetic analyses
Genetic factors may influence both vulnerability to cognitive decline and the impact of lifestyle factors on memory performance and underlying neurobiological mechanisms. For example, there is evidence that protective effects of physical activity on hippocampal atrophy are enhanced in carriers of the ApoE ε4 allele, a risk gene for Alzheimer's disease[57]. Another study showed a significant interaction between the COMT- polymorphism [Val158Met], which plays a central role in cortical dopamine degradation, and dietary intervention[58]. Accordingly, homozygous Val/Val carriers exhibited greater improvement by, among other things, caloric restriction. The Met variant of the BDNF polymorphism [Val66Met], a genotype associated with abnormal hippocampal and memory function, interacted with physical activity in relation to cognitive decline and dementia incidence rates in the elderly[59].

The significance of functional polymorphisms on the response of memory-enhancing dietary interventions in older people at increased risk of dementia is unclear. Therefore, this study will analyze genetic polymorphisms (e.g., ApoE, BDNF, COMT, TOMM40 rs10524523) that have been linked to energy metabolism and memory performance and could lead to differences in response to polyamines in carriers of the above polymorphisms.

Positron emission tomography
Patients with SCD are more likely to show initial Aß deposition in the brain compared to healthy elderly without SCD[4]. In the present study, study participants will be examined and characterized with respect to this biomarker by PET. As previously described, a reduction in polyamine concentration is associated with a reduction in cellular autophagy processes[52]. It is hypothesized that a potentially protective effect of polyamines on the age-associated cognitive decline is mediated via an increase in autophagy[1], a process that, among other things, clears the cell from harmful protein aggregates such as Aß plaques[60, 61]. It has been shown that even low concentrations of spermine, spermidine, and putrescine reduce the toxicity of Aß oligomers[62].

It is therefore of great interest to investigate to what extent an incipient Aß pathology influences the effects of a 12- month polyamine supplementation on cognitive abilities of the study participants and whether the cellular autophagy processes are influenced by the supplementation (determined via the metabolite status in the muscle tissue, see also "Significance of an investigation of muscle tissue and muscle function"). An absolute Aß cell purification and thus change of

	the Aß status is not expected by the polyamine intervention, so that the PET can also be performed during the course and not essentially at the beginning of the intervention.
10. Description of the planned measures/examination methods and any deviations from the measures/examinations commonly used in medical practice (what is "routine", what is done differently in the study?)	**Course of the study**<br><br>**SCD cohort**<br><br>**At the beginning of the study, time point 1 (T1)**<br><br>**Visit 1**, duration approx. 5-6 hours (incl. breaks)<br>**- Participant information and consent**<br>The subject or patient is informed verbally and in writing about possible participation in the study. If, after sufficient time for reflection, written consent to participate in the study has been given (informed consent), inclusion in the study takes place and a unique identification number is generated for subsequent pseudonymization. The consent can be revoked at any time.<br><br>**- Entrance examination**<br>The initial examination is divided into screening and baseline.<br><br>*Screening*<br>Here it is clarified whether the study participant can take part in the study without health risks and whether the inclusion ability is guaranteed.<br><br>The following information will be collected from all study participants for screening:<br><br>a) medical and demographic data, incl. nutritionally relevant parameters, such as height, weight, waist-hip ratio<br>b) questions about subjectively perceived cognitive deterioration and related concerns[10]<br>c) objective measurement of cognitive abilities using standardized neuropsychological screening tests<br>d) questionnaires on depression and daily activity<br>e) examination of the MRI/PET suitability<br>f) clarification of possible contraindications regarding the muscle biopsy<br><br>For more details, see "Description of methods/tasks used".<br><br>*Baseline*<br>If all inclusion criteria are met and there is no exclusion criterion, the participant will be included in the study and further investigated as follows:<br><br>g) endogenous polyamine concentration in blood plasma<br>h) analysis of peripheral blood markers: including inflammatory processes, glucose metabolism, neurotrophin, lipid and fatty acid profile<br>i) detailed neuropsychological testing using standardized test procedures. The following functional areas are tested: Verbal and visuo-spatial memory, attention, executive functions, sensory-motor speed<br>j) validated questionnaires are used to further characterize the sample, including those on sleep quality and lifestyle<br>k) examination of peripheral vascular processes (measurement of arterial stiffness by means of an arteriograph)<br>l) investigation of genetic polymorphisms in blood

	m) determination of the hair cortisol level (participation optional)<br><br>For more details, see "Description of methods/tasks used".<br><br>**Visit 2**, duration approx. 1.5 hours<br>n) structural and functional imaging of the brain (MRI)<br><br>**Visit 3**, duration approx. 1.5 hours<br>o) ^{18}F FBB-PET (determination of Aβ status, participation optional)<br><br>**Visit 4**, duration approx. 2-3 hours<br>p) muscle biopsy (to determine autophagy; participation optional) and measurement of muscle function and muscle mass (sarcopenia testing)<br><br>**<u>Intervention:</u>**<br>This is a randomized placebo-controlled study design. Study participants are randomized into groups (stratified randomization by age and gender) and participate in an intervention (polyamine group) or control condition (placebo group) over 12 months.<br><br>During the intervention period, participants are blinded to the experimental condition (polyamines, placebo). Likewise, the researcher collecting the measurements is blinded to the experimental condition before, during, and after the intervention.<br><br>The polyamine group receives polyamine supplementation in capsule form (750 mg lyophilisates from wheat germ, equivalent 10 g wheat germ/day, equivalent to 10 g "natto"/per day (see also point 9).<br><br>The control group receives placebo capsules filled with cellulose.<br><br>All capsules are identical in appearance (blinding), and are ingested by the study participants in the same manner at mealtimes.<br><br>Study participants will receive detailed information on capsule use and capsules for the first 6 months in an individual onset session (30 minutes) at the end of visit 1.<br><br>**<u>Progress survey:</u>**<br><br>**Visit 5**, duration approx. 15 minutes<br><br>After 6 months, a personal interview is held with the participant regarding his or her mental and physical condition and compliance is determined. In addition, the participant receives new capsules, and the old capsules are collected and counted.<br><br>**<u>Final examination, time point 2 (T2)</u> (1st follow-up, 12 months after the start of the intervention)**<br><br>**Visit 6,** duration approx. 4-5 hours<br>Repeat the measurements described above [a-k].<br><br>**Visit 7,** duration approx. 1.5 hours<br>Repeat the measurement described above [n].<br><br>**Visit 8,** duration approx. 2-3 hours<br>Repeat the measurement described above [p].

	**Follow-up, time point 3 (T3) (2nd follow-up, 18 months after the start of the intervention)**<br><br>**Visit 9**, duration approx. 3 hours<br>Repeat medical examination with blood sampling [a, g, h], measurement with arteriograph [k], abbreviated psychometric testing [c, i], and the collection of validated questionnaires [d, j].<br><br>**Reference cohort**<br>The following studies will be conducted equivalently to the SCD cohort:<br><br>**Visit 1**, duration approx. 5-6 hours (incl. breaks)<br>- participant information and consent<br>- initial examination (screening + baseline) [a-l, except f and m]<br><br>**Visit 2,** duration approx. 1.5 hours<br>- MRI examination [n]<br><br>**Visit 3,** duration approx. 1.5 hours<br>- PET examination [o]<br><br>**Description of the methods/tasks used**<br>*Psychometrics:*<br>Validated paper-and-pencil and computer-based tests will be used with study participants in both cohorts.<br><br>Participants undergo neuropsychological screening to objectively measure cognitive status. Tests used for this purpose include: Mini Mental State Examination[67], Wechsler Memory Scale Logical Memory II[68], Trail Making Test A (TMT A)[69]. In addition, questionnaires on depression (Geriatric Depression Scale, GDS[70]) and daily activity (Instrumental activities of daily living, IADL) are to be completed during screening.<br><br>Subsequently, the visual and verbal learning and memory abilities of the study participants are assessed using the following tests: Auditory Verbal Learning Test (AVLT)[71], People-and- Door Test[72] and a computer-based sensitive and validated visual memory task, so-called Pattern Separation Test[73, 74]. Executive functions, attention and working memory as well as sensorimotor speed are assessed among others by the following tests: Digit Span[75], Corsi Block Tapping[76], Trail Making Test A and B[77], Digit Symbol Coding[78], Semantic and Phonological Word Fluency (Regens- burger Word Fluency Test, RWT), Stroop Test[79], and Attention Assessment Subtests (TAP)[80].<br><br>Questionnaires on sleep quality (Pittsburgh Sleep Quality Index[81]) and lifestyle (smoking and drinking habits, physical and mental activity, quality of life (Freiburger physical activity questionnaire[82]), general subjective well-being (Positive and negative Affective Schedule, PANAS[83]) are also to be completed.<br><br>In terms of psychometrics, we cooperate with the neuropsychologist Dr. Ute Kopp of the Charité.<br><br>**Laboratory parameters (blood):**<br>Study participants in both cohorts will have the following parameters determined, among others:<br>- polyamine concentrations (spermine, spermidine, putrescine)<br>- basic parameters including small blood count, liver and kidney values, triglycerides, LDL and HDL cholesterol, coagulation (PTT, Quick)[84, 85]

	- glucose metabolism: glucose, insulin, HbA1c, leptin<br>- inflammatory parameters: hsCRP, TNFα, IL-6, IL-1b, fibrinogen, among others<br>- neurotrophins: BDNF, GDNF, NGF, G-CSF, IGF-1, among others<br><br>The analyses of the various blood parameters are performed in the "Alterung & Zelltod" - Laboratory under the direction of Prof. Dr. Frank Madeo (Institut für Molekulare Biowissenschaften, Graz, Österreich), in the laboratories headed by Prof. Dr. Stephan Sigrist (Freie Universität Berlin, Institut für Biologie/Genetik, Berlin and NeuroCure Charité - Universitätsmedizin Berlin, Campus Mitte, Berlin), in the laboratory headed by Prof. Dr. Guido Kroemer (Centre de Recherche des Cordoliers, Paris, France), in the Clinic and Polyclinic for Neurology under the direction of Prof. Dr. Agnes Flöel (Universitätsmedizin Greifswald) and by the contract laboratory Berlin - Charité Vivantes GmbH.<br><br>***Laboratory parameters (CSF, optional for the SCD cohort; reference cohort not concerned):***<br>If a medically indicated CSF diagnosis is planned for the study participants during the study or if a CSF diagnosis was performed within the last 12 months (only in the SCD cohort), only the results of this clinical diagnosis will be used within the framework of the above-mentioned study after the participant has been informed and has given his consent. For study purposes only, no lumbar puncture will be performed, and no cerebrospinal fluid will be collected for additional analysis.<br><br>***Determination of genetic polymorphisms:***<br>DNA is extracted from peripheral blood samples using standard methods (Qiagen kit). Genotyping will be performed at Martin Luther University Halle-Wittenberg, Medical Faculty Hospital, University Clinic and Polyclinic for Psychiatry by Prof. Dr. med. Rujescu.<br><br>***Measurement of arterial stiffness:***<br>To determine vessel elasticity/stiffness, two parameters are measured using an arteriograph: Pulse wave velocity and augmentation index. Pulse wave velocity is the pressure wave generated by the contraction of the heart, which travels through the arterial vascular system at a certain speed (normally 6 - 12 m/s). The augmentation index, on the other hand, describes the share of the pressure increase caused by the reflected pulse wave in the total pulse pressure.<br><br>***Hair cortisol determination (optional for the SCD cohort; reference cohort not concerning):***<br>For hair cortisol determination, 10 mg of a hair segment is required. For this purpose, thin hair strands (2-3 hair strands) are cut off at the back of the head, tied together with a thread about 2 cm above the skull bone and then cut off as close as possible to the hairline. The hair samples are wrapped in aluminum foil and can be kept at room temperature for a longer period. Participation in hair cortisol determination is optional. In the case of study participants who consent to hair cortisol determination, the hair sample is taken once during the study.<br><br>***Magnetic resonance imaging (MRI):***<br>The MRI examination will take place at the Berlin Center for Advanced Neuroimaging (Campus Charité Mitte, Charité - Universitätsmedizin Berlin, Charitéplatz 1, 10117 Berlin). The MRI measurement of the brain at 3 Tesla includes structural and functional sequences.

For structural imaging, the following three sequences are used: T1-weighted 3D sequence with isotropic resolution of at least 1 x 1 x 1 mm^3, Fluid Attenuated Inversion Recovery (FLAIR) sequence, and Diffusion tensor imaging (DTI) sequence. The aim of structural MRI is, among other things, to determine the volume of Alzheimer-specific brain regions and the integrity of gray/white matter.

The following three sequences are used for functional MRI imaging: Arterial Spin Labeling (ASL) Sequence, BOLD sequence during a validated visual memory task[73, 74] and BOLD sequence at rest (resting-state connectivity). The aim of functional MRI is, among other things, to investigate vascular processes (brain perfusion) and functional connectivity of vulnerable brain regions both at rest and during a specific task.

The MRI images are analyzed using standard methods, including "Voxel-Based Morphometry" and "Tract-Based-Spatial Statistics". Established software packages are used (SPM and Matlab, FSL, Freesurfer and AFNI).

Positron emission tomography (PET) (optional for all study participants):
The burden of amyloid-ß plaques on the brain is performed by PET with the approved plaque ligand ^{18}F-florbetaben (trade name NeuraCeq). Imaging is performed with the PET/MR hybrid system (Siemens mMR) of the Charité at Campus Virchow. Participation in the amyloid PET is optional. In study participants who consent to amyloid PET, amyloid PET will be performed once during the study.

For amyloid PET, 300 MBq of ^{18}F-florbetaben is administered intravenously. Immediately thereafter, an early image of the florbetaben distribution in the brain is taken (duration: 10 minutes). A late image of the florbetaben distribution in the brain is taken 90 minutes after florbetaben administration (duration: 20 minutes).

Quantitative analysis of amyloid PET is based on predefined regions of interest. In addition, explorative voxel-based evaluations are performed.

Muscle biopsy (optional for SCD cohort; reference cohort not concerning):
The muscle biopsy will be performed at the Clinical Research Unit, Berlin Institute of Health (BIH) at Charité Campus Mitte.

The muscle biopsy will be performed on the thigh (Musculus quadrizeps). For this purpose, local anesthesia with lidocaine 2% (without epinephrine) is performed. A scalpel is then used to make a minimal skin incision (3-5 mm) in the anesthetized skin area. The tissue biopsy is performed with a semi-automatic biopsy device (CR Bard GmbH, Karlsruhe, Germany) with a 12G muscle biopsy needle (Bergstrom). Four samples (each approx. 50-100 mg muscle tissue) are obtained from the affected area through the same skin incision. After the biopsy, which takes only a few minutes, is completed, pressure is applied to the wound for about 5-10 minutes to stop bleeding and prevent hematoma formation. The skin edges are then closed with steristrips and covered with another sterile bandage plaster. In addition, a lightly compressing bandage with elastic bandages is applied for the following 12 hours. Although rest and recuperation are not absolutely necessary after the procedure, we recommend that the patient rest for about 24 hours (no sports, no cycling), although general physical activities are possible without any problems.

	In order to additionally test the potential positive influence of the polyamine intervention on possible age-associated sarcopenia symptoms, the following frequently used test battery for muscle function/muscle strength will be applied to the study participants: SPPB (Short Physical Performance Battery) including a 6-meter running test and a hand grip strength test. Furthermore, a non-invasive, painless bioelectric impact analysis (/) can be used to make a statement about early changes in muscle mass (pre-sarcopenia). During the BIA measurement, the study participant remains at rest for about 10 minutes until the blood volume is evenly distributed throughout the body. Via two skin electrodes each on hand and foot, an alternating current field is generated and the impedance of the body to this alternating current is measured.<br><br>**Representativeness of study participant selection**<br>To record all participants suitable for the study, a list is kept by the investigator (trial registry) showing how many meet the inclusion and exclusion criteria but were not included in the study for various reasons.
11. Evaluation and weighing of the foreseeable risks and disadvantages of study participation against the expected benefits for the study participants and persons who will become ill in the future (risk-benefit analysis)	Overall, the risk of health damage from the examination is to be assessed as minimal, taking into account the contraindications (for details see point 11c).<br><br>Study participants will be informed about all possible risks of the study and that they can withdraw their consent at any time without giving reasons (see attached study information). The expected "drop-out" of participants is balanced by the number of participants (see case number analysis).<br><br>All adverse effects (AE and SAE see below) arising from the study will be documented as standard.<br><br>***Adverse Events (AE)*** of the planned study: any undesirable or unfavorable event that may affect a study participant, including abnormal findings (e.g., abnormal physical examinations or laboratory findings), symptoms or illnesses that occur during study participation, regardless of whether they are directly related to study participation.<br><br>***Serious Adverse Events (SAE)*** of the planned study:<br>any occurrence which:<br>- is life threatening<br>- leads to hospitalization<br>- leads to serious physical impairments<br>- any other occurrence that is judged by the study director to be a significant hazard<br><br>In the event of the occurrence of an SAE:<br>The investigator first assesses whether a causal relationship with the use of the investigational product is considered possible. Since this is a double-blind study, this is done without knowing the group membership (polyamines vs. placebo).<br><br>Assignment to the individual treatment groups will be made only after unblinding as part of the evaluation of the study.<br><br>The exception is an emergency unblinding, which the investigator may perform if the decision on the further treatment of the affected patient depends on the knowledge of the study treatment. If more than three of the enrolled study participants in the intervention group have SAEs that are likely to be related to polyamine use (as judged by the study physician), the study will be terminated.

a. Foreseeable therapeutic benefit for the study participants (**individual benefit** for the individual study participant)	It is known from animal studies that polyamine-rich supplements can have positive effects, e.g., improved memory and an increase in autophagy processes. The first promising results of the preliminary study (with a short intervention period of 3 months) also reflect that polyamine supplementation over a longer period could lead to a significant improvement in memory function.<br><br>The measurement of autophagy in tissues is central to understanding the underlying mechanisms that may explain the potential positive effect of polyamine intervention on cognitive performance in patients with SCD. Furthermore, it can be assumed that an increase in the autophagy rate through polyamine administration leads to improved muscle function of the study participants and thus to an improved quality of life.<br><br>In summary, according to the assessment of the data to date, a positive effect can be expected for the study participants in the polyamine group. However, even if individually "only" an improvement in well-being/quality of life could be achieved, this would be considered positive for the individual.<br><br>A direct individual benefit for the study participants of the control group and the reference cohort is not expected. Nevertheless, the study participants will receive extensive medical and neuropsychological testing, which will provide information about their current health status.
b. Foreseeable medical benefit for persons who will become ill in the future **(group benefit)**	If the intervention has a positive effect, new possibilities will arise in the treatment of elderly persons with subjectively perceived cognitive deterioration. The polyamine-rich dietary supplement is an easy-to-use application. In addition, the non-invasive treatment approach has a very good safety profile[21] (see also data from our own preliminary study under point 9).<br><br>From a medical-scientific point of view, the investigation of changes in brain function and structure as well as changes in autophagy processes is particularly important. So far, the exact mechanisms of long-term polyamine-rich dietary supplementation have been little studied.<br><br>For this purpose, also the comparison of whether certain genetic constants or early biomarkers (Aß pathology) can predict a good or less good response to the intervention is of great interest for a possible therapeutic use.<br><br>The extensive investigation of different biomarkers should enable a better understanding of the underlying mechanisms of a polyamine intervention to ultimately create a starting point for the prevention and early intervention of Alzheimer's disease in the long-term through the targeted use of the intervention.
c. **Risks** and burdens for study participants (list all in detail)	Study participants will be under medical supervision and treatment throughout the study.<br><br><u>Taking a polyamine supplement in capsule form, obtained from wheat germs</u>: Polyamine-rich foods such as "Natto" (made from soy) or wheat germ are generally very well tolerated. Basically, as with all foods, intolerances, or allergies to the concerned food are possible. In this case, the concerned food should not be consumed. Ingestion of wheat germs or the corresponding extracts may cause mild gastrointestinal complaints, such as belching and diarrhea, which, however, disappear completely after discontinuation. Belching can be reduced or prevented by taking the capsules with meals and is therefore recommended to the

	participants.<br>Since wheat products generally contain gluten, polyamine substitution with wheat germ derivatives should not be performed in cases of known gluten intolerance.<br><br>The polyamines putrescine, spermine and spermidine in the suture also delay histamine oxidation by diamine oxidase (DAO). Therefore, polyamine supplementation is not performed in cases of known histamine intolerance.<br><br>In previous work on polyamine substitution in high doses, no specific side effects beyond these have been reported[21]. Also in the preliminary study, there were no side effects indicating intolerance of the polyamine dose also used here.<br><br>*Psychometric testing:* is not associated with any special risks, apart from fatigue phenomena in individual cases.<br><br>*Venous blood sampling* can lead to complications such as hematoma, infection, or pain at the injection site, but these complications are extremely rare, easily treatable and completely reversible.<br><br>*Measurement of vascular elasticity/stiffness (arteriograph)* as part of our examinations is painless and takes very little time.<br><br>*Hair sampling:* is not associated with any special risks.<br><br>*MRI:* is a non-invasive procedure that is used worldwide and does not pose a risk to study participants if the relevant exclusion criteria (see point 16) are observed.<br><br>*PET:* PET examinations have been reliable and well established for several years in healthy subjects (and clinical patients) at the Virchow-Clinic of the Charité. The application of a weakly radioactive substance requires exposure to radiation. For the amyloid PET examination, the study participant is injected with 300 MBq of ^{18}F-florbetaben (trade name NeuraCeq) according to the approval. Intravenous injection of ^{18}F-florbetaben causes a radiation exposure of 19.3 µSv/MBq. Thus, application of 300 MBq of ^{18}F-florbetaben is associated with an effective dose of 5.8 mSv. The limit of 20 mSv specified in §24 paragraph (2) of the Radiation Protection Ordinance of July 20, 2001, is therefore clearly undercut in any case.<br><br>The risk from the total radiation exposure of 5.8 mSv is limited to stochastic radiation effects, with the causation of radiation-induced cancer playing the dominant role. Deterministic radiation effects can be excluded for radiation exposure < 100 mSv.<br><br>The "official" lifetime risk of radiation induction of a fatal cancer has been set by the International Commission on Radiological Protection (ICRP) at 5 %/Sv[86]. This value applies to the population as a whole, i.e., represents an average value across all age groups. For persons aged > 60 years, the lifetime risk of radiation induction of a fatal cancer is about 1 %/Sv[86]. For the persons to be included in the requested study, the calculated risk of a fatal cancer induced by ^{18}F-florbetaben PET is therefore about 0.00006. The lifetime risk of a "spontaneously" occurring fatal cancer is about 0.2.<br><br>Complications such as hematoma, infection, or pain at the injection site may occur during flexure insertion, but these complications are extremely rare, highly treatable, and completely reversible.

	The probability of an allergic reaction to the injected radioactive substance is considered low. Participants are asked about known intolerances and are under medical supervision during the entire examination.<br><br><u>*Muscle biopsy:*</u> If there are no complications, a small pigment shift could occur. However, infection and hemorrhage can also occur as complications during biopsies. The risk of infection is minimized by aseptic work. Bleeding is treated by compression. Prophylactically, a pressure dressing is applied after muscle biopsy. However, the occurrence of reversible hematomas without complications cannot be ruled out. These are treated conservatively with cooling and, if allowed, with ointment dressings. Larger hemorrhages/ complications cannot be ruled out with certainty; if necessary, these will be treated according to medical requirements. In order to recognize and treat possible complications in time, the study participants are requested to contact the study center at any time in case of complaints or to present themselves in our department. A known hypersensitivity to anesthetics will be excluded as far as possible.<br><br>All muscle biopsies are taken at the Clinical Research Unit of the Berlin Institute of Health (BIH). The performing physicians have sufficient and long-term experience of 3-10 years (more than 500 biopsies in different studies), so that the procedure is performed routinely. In several studies (e.g., EA2/017/09, EA2/050/10) repeated (>2) muscle biopsies per study participant were performed, showing good tolerability. In the present study, only one biopsy will be performed at the beginning and one at the end of the intervention with an interval of 12 months, so that the expected tolerability is considered to be good.<br><br>The performance of a BIA measurement and the sarcopenia tests for muscle function are painless and no major side effects are to be expected. Only slight, easily treatable skin irritation could occur due to the electro- den patches, although this risk is considered to be very low.
12. Risk control measures	The following safety measures should help to minimize the risks mentioned under point 11c in this study (see also explanations under point 11c):<br><br>1) Compliance with inclusion and exclusion criteria is strictly monitored. Study participants may discontinue the study at any time (even during the trial).<br><br>2) All participants receive an initial neurological and medical examination.<br><br>3) All examinations are performed by experienced investigators. In case of undesirable side effects, access to emergency measures is provided immediately. Participants are informed of all possible risks and that they can withdraw their consent at any time without giving reasons.<br><br>4) MRI examinations of the brain are performed after detailed questioning regarding possible risks or contraindications (see point 16). If these contraindications are considered, the risk of the MRI examination is minimal.<br><br>5) Participants in amyloid PET are urged to drink plenty of fluids after the examination, as frequent bladder emptying after the examination further reduces radiation exposure.

	6) Reference is made to the explanations given under point 11 regarding measures to ensure safety during muscle biopsy.<br><br>7) Study participants are requested to contact the investigator or the Neurological Polyclinic immediately in case of health problems. Appropriate telephone numbers will be handed out.
13. Termination criteria	Any participant may withdraw from the study without giving a reason. Possible reasons for dropping out may be:<br><br>- withdrawal of consent<br>non-tolerance of the MRI/PET examination (claustrophobia)<br>- non-tolerance of regular intake of polyamine supplementation or placebo capsules<br>- occurrence of other serious events that necessitate termination
14. Number, age and sex of the persons concerned	The **SCD cohort** includes 100 elderly patients with subjectively perceived cognitive decline and related concerns, aged 60-90 (objective cognitive testing still within norm (< -1.5 SD), both men and women (see case number estimate).<br><br>The **reference cohort** provides for 50 cognitively intact elderly persons without subjectively perceived cognitive deterioration and related concerns aged between 60 and 90 years, both men and women.
15. Biometric planning with indication of the statistical methodology, including justification of the number of cases. Specification of the statistician(s)	**<u>Experimental design:</u>**<br>This is a randomized placebo-controlled trial design.<br><br>**<u>Dependent variables:</u>**<br>*Primary*: change in memory performance compared between baseline (T1) and end of intervention (T2).<br><br>*Secondary*: The following changes will be tested in comparison between baseline (T1) and end of intervention (T2):<br><br>1) performance in other cognitive domains<br>2) autophagy and muscle function<br>3) blood parameters<br>4) vascular parameters<br>5) MRI parameters<br><br>In addition, a multimodal analysis will be performed with different sensitive biomarkers in relation to a healthy reference cohort.<br><br>For a detailed description of the measurement methods and target parameters, see items 4 and 10.<br><br>**<u>Evaluation:</u>**<br>The primary hypothesis is tested using analysis of covariance. Memory performance (measured by the Pattern Separation Test) at the end of the study (follow-up, T2) is the dependent outcome measure; the covariate is memory performance at the beginning of the study (baseline, T1). Memory performance between the polyamine group and the placebo group is tested.<br><br>The secondary parameters are analyzed analogously.<br><br>Furthermore, the influence of the polyamine intervention on various sensitive biomarkers compared to the placebo intervention will be determined by means of a so-called multimodal analysis. Using a reference cohort, the raw values of the individual biomarkers of the SCD

	cohort will be z-transformed (using the mean and standard deviation of the reference cohort) and adjusted for normal age and sex variability in order to achieve scale invariance and to avoid bias due to normal age- and sex-typical changes in the above-mentioned biomarkers. Finally, a multivariate analysis of variance is used to determine which biomarkers are most strongly changed by the intervention and can thus influence cognitive abilities.<br><br>In addition, we will investigate whether genetic polymorphisms or Aß deposits are associated with cognitive performance or modulate the response to the intervention by including them as covariates (or as interaction terms with group membership) in the statistical models.<br><br>The analysis of follow-up is also performed using analysis of covariance, with the parameters at T3 (after 18 months) representing the dependent outcome variables, group membership forming the independent variable, and the parameters at T1 (baseline) being inserted as covariates.<br><br>The main hypothesis is tested at a two-sided significance level of alpha=0.05.<br><br>**PROPOSED SAMPLE SIZE/POWER CALCULATION**<br>In the preliminary study, ANCOVA yielded an effect size (partial Eta squared, η^2) of 0.058 for the group difference in the pattern separation test, which corresponds to Cohen's d effect size of 0.5. Corrections were made for age, gender, and education.<br><br>Cohen's d was calculated according to the formula:<br>$$d = \frac{2 * \eta}{\sqrt{1 - \eta^2}}$$<br>Case number estimation was performed conservatively using a t-test for differences (follow-up value - baseline value) in memory performance (BPS score) in the two groups (polyamine and placebo) even when the analysis is performed using analysis of covariance.<br><br>In this study, we expect a stronger effect size of Cohen's d of 0.6 due to a longer intervention. If 50 participants per group (incl. 10% drop-out rate) are included in the analyses, such an effect strength can be demonstrated by means of t-test for unconnected samples (two-sided significance level α=0.05, power: 80%).<br><br>This case number estimate was compiled using the R package "pwr"[87].<br><br>__**Randomization**__<br>Stratified randomization by sex and age (60-70, 70-80, 80-90 years) will be performed.<br><br>The study is biometrically supervised by Dr. U. Grittner.
16.<br>a. Presentation and, if necessary, explanation of the **inclusion and exclusion criteria**	__**Inclusion criteria of all study participants:**__<br>- age: 60-90 years<br>- existing health insurance for clarification of possible incidental findings<br>- capacity to consent<br>- age-appropriate performance on neuropsychological screening tests (no more than 1.5 SD below gender-, age-, and education-adjusted norms)<br><br>__Specific to SCD cohort:__

	- notification by the person of subjectively perceived cognitive deterioration (for at least 6 months) and concerns in this regard<br>- and that they would see or have already seen a doctor because of the subjective cognitive deterioration.<br><br>**<u>Exclusion criteria all study participants:</u>**<br>- DSM-IV manifest dementia<br>- severe or untreated internal diseases (advanced arteriosclerosis, advanced cardiac or respiratory disease, untreated thyroid dysfunction, or untreated diabetes mellitus), psychiatric (untreated depression; psychosis) or neurological (epilepsy, clinically manifest stroke) diseases<br>- malignancies current or in the medical history (exception: basalioma)<br>- drug / medication / alcohol addiction<br>- non-MR-capable individuals [individuals with claustrophobia, metallic implants (e.g., intracranial metal clips), and wearers of electronic devices (e.g., pacemakers) or tattoos]<br>- participation in another study involving the use of ionizing radiation in the last 3 months<br>- restrictions in other everyday activities<br><br><u>Specific to SCD cohort:</u><br>- already existing polyamine substitution or participation in corresponding intervention studies<br>- known intolerance or allergies to wheat sprouts or known gluten intolerance or histamine intolerance<br>- when performing a muscle biopsy: allergy or intolerance to the local anesthetic (especially lidocaine), coagulation disorders, current therapy with clopidogrel, ASA, Marcumar, or Falithrom<br><br><u>Specifically for reference cohort:</u><br>- subjectively perceived cognitive deterioration and related concerns, communicated by the person
b. **Participant information** (who gives this verbally and indication of how much time remains between informing and consenting, otherwise reference to its content as an attachment possible)	see study information attached<br>1. SCD Cohort<br>2. Reference Cohort
c. **Declaration of consent** (reference to its content as an attachment is possible).	see study information attached<br>1. SCD Cohort<br>2. Reference Cohort
d. If applicable, **information and consent of the legal representative** (if applicable, also description of the procedure for the establishment of a judicial guardianship)	Not applicable, as only study participants who can give their own consent are included.
17. Measures for the recruitment of study participants (notice board ?, newspaper advertisements ? etc.)	The study is advertised in the Berlin memory consultations. In addition, there will be information and contact opportunities for the study on the Internet, e.g., on the website of the Clinic for Neurology of the Charité and on www.pro- bandeninfo.de. Depending on the recruitment success there will be additional advertisements in Berlin newspapers. Recruitment will continue to take place via notices in adult education centers or senior citizens' associations, contacting the BANA (Berlin

	model for post-professional activities in which people over 45 have access to university continuing education).
18. If applicable: **reason for inclusion and statement of therapeutic benefit for persons who are minors and/or unable to consent**	Not applicable
19. Relationship between study participant and study physician (Is the study physician also the treating physician?)	Study participants are not dependent on the study physician.<br><br>For recruitment within the memory clinic Charité Campus Mitte as well as for recruitment via non-own memory clinics, the study physician will be the non-treating physician.
20. Statement on the possible involvement of persons dependent on the sponsor or study physician	Study participants enrolled in the study are not dependent on the study physician or a sponsor.
21. Measures that allow a determination of whether a study participant is participating in more than one study at the same time or before the end of a period specified in the previous study	During the initial telephone contact, prospective study participants are asked about prior/simultaneous other study participation, and if so, in which one(s).
22. If applicable: remuneration or reimbursement of the study participants (amount, for what should be paid ?)	At the end of the study, study participants in the SCD cohort will be paid an expense allowance (one-time lump sum for time and travel expenses) of a maximum of 180 Euros (100 Euros study participation, 40 Euros for PET and 40 Euros for muscle biopsy).<br>Participants in the reference cohort will receive an expense allowance of maximum 85 euros (45 euros study participation and 40 euros for PET).
23. If applicable: plan for follow-up and medical care of the persons concerned after the end of the study	If participants are recruited from memory consultations, then they will be followed up as part of the respective memory consultation.
24. If applicable: insurance of the study participants (insurance confirmation and insurance conditions, insurer, scope of insurance, duration of insurance)	An insurance policy for volunteers will be taken out with HDI-Gerling Industrie Versicherung AG (Riethorst 2, 30659 Hannover, Germany, insurance certificate no. 5701032603017).<br>The maximum benefit payable by the insurer per insured person is € 250,000.00. A copy of the insurance certificate and the terms and conditions of insurance will be given to each study participant.
25. If applicable: documentation procedure (reference to CRF sheets possible).	A study file will be maintained to collect and document all study data (demographic data, psychometric test results, etc.) in pseudonymous form.<br><br>**The following personal data is collected**<br>- age, gender, date of birth, level of education, address, health/disease data<br><br>**Data categories**<br>- study data<br><br>**Survey type**<br>- data is collected both via paper pencil and computer-assisted testing<br>- the tests do not contain clear names, but the pseudonym defined for each participant (see point 28)

	- all data is transferred and stored in a digital data matrix(s) in pseudonymized form<br><br>**Handling data and samples**<br>Test results and study data<br>- participants have the right to request information; this can be done verbally or in the form of printouts (the study participant can only be assigned to the test results via the participant identification list)<br>- pseudonymized study data of study participants who consent to a PET examination may be shared with the tracer manufacturer (Piramal Imaging GmbH, Tegeler Straße 6-7, 13353 Berlin, Germany)<br>- pseudonymized study data will be passed on to the co-study director Prof. Dr. med. Agnes Flöel (Klinik und Poliklinik für Neurologie, Universitätsmedizin<br>- Greifswald, Ferdinand-Sauerbruch-Strasse, 17475 Greifswald, Germany).<br><br>Blood and muscle biopsy samples<br>- are blinded, i.e., given a code (see point 28), age and sex (the latter for reference values) and sent to the laboratories mentioned in point 30 for the purpose of the study<br><br>Hair samples<br>- are blinded and sent to the laboratory mentioned in point 30 for the purpose of the study<br><br>MRI data<br>- are transmitted pseudonymously to a neuro-radiologist for diagnosis<br>- in the case of abnormal findings, the study participant is identified via the participant identification list (see point 28) of the pseudonyms and, if necessary, informed of this fact<br>- the actual MRI analysis regarding the questions within the scope of the study is also carried out pseudonymously<br><br>PET data<br>- the PET analysis is pseudonymized within the scope of the study<br><br>**Storage**<br>Personal data<br>- are stored in accordance with good scientific practice for a period of 10 years, after which they are deleted and, if necessary, only used in purely anonymous form<br><br>Samples<br>- are stored in the respective analytical laboratories for a maximum period of 10 years after the end of the study for possible follow-up testing and are then destroyed<br><br>MRI/ PET data<br>- the MRI and PET images are stored and archived digitally on the server of the working group in pseudonymized form<br><br>**Access to the data**<br>- only the members of the SmartAge study team involved in the study will have access to the personal data. All employees of the SmartAge study team are subject to the obligation of secrecy

26. If applicable: description of how the health status of healthy affected persons is to be documented	The study participants are asked about their health status regarding certain criteria (see point 16). The health status of the study participants will be recorded on the documentation sheet.
27. If applicable: methods to identify, document and report adverse events (when, by whom and how??)	During the examination, the participants will be in direct contact with the supervising research assistants and neurologists. For the time after completion of the examination and in case of any side effects, a telephone number is provided where a neurologist can always be reached.<br><br>During the 12-month intervention phase, an additional visit and two telephone calls are planned to document compliance and tolerability of the interventions. Adverse events will also be asked about and documented.<br><br>So-called incidental findings may occur in the study participants (e.g., previously unknown brain structural changes); therefore, all MRIs will be reviewed radiologically by a specialist. If necessary, the study physician will inform the study participant about any abnormalities in this regard.
28. Procedure to protect the secrecy of the stored data, documents, and samples, if applicable, explanation of the coding of the data of study participants (please do not use initials and date of birth as coding scheme!).	All study-related data will be collected and stored in pseudonymized form after inclusion of study participants. Study-related data collected in paper form (tests, questionnaires) will be stored in a lockable cabinet in an office within NCRC. If study participants indicate on the test and questionnaires personal data, such as name and date of birth, these are blacked out and the documents are only stored in pseudonymous form. The data is then reused in electronic form. Once the data has been entered in electronic form, it is stored on a password-protected computer in the working group.<br><br>The following code is assigned: Letters "SMART" (SCD cohort) or "SMART-REF" (reference cohort) plus three-digit number for the respective study participant (e.g., "001" for the first participant), plus code for the respective measurement (for pre-intervention T1, for post-intervention T2, follow-up T3), for example: "SMART001-T1" or "SMART-REF001-T1".<br><br>The respective identification codes of the study participants are recorded in a separate file (participant identification list) and stored on the server of the working group, which has a separate data protection concept for this. The creation of a file with personal information is reported to the data protection officer of the Charité.<br><br>Paper copies of the study participants' consent forms are kept in a lockable cabinet in the SmartAge study team's office. The folder is located in an office within the NCRC.<br><br>**Access rights**<br>During and after the study (see point 25 "Access to the data")<br><br>**Procedure and deadlines for deletion, blocking of data**<br>The personal data will be stored in accordance with the evidence of good scientific practice for a period of 10 years and then deleted.<br><br>Participants may revoke their consent to participate in the study at any time. They can also object to the further processing of the collected data at any time and request its deletion or destruction. All data will then be destroyed immediately. The data will be manually deleted from the PCs.

	Furthermore, data in paper form (questionnaires, tests) are destroyed by shredding. Back-up DVDs are also destroyed.
29. Statement on compliance with data protection<br>- assurance that all data collected and stored about the study participant will remain data will be treated confidentially (data secrecy and medical confidentiality)<br><br>- assurance that the identifying Data will be accessible only to the study director or staff designated by the study director<br><br>- indication of the measures taken to ensure confidentiality<br><br>- measures for the data-protection-compliant transfer of data that do not allow third parties to establish a personal reference<br><br>- indication of the information, objection, and deletion options<br><br>- measures to ensure the rights of the participants	The data/information collected as part of this study is stored pseudonymously on the AG server (drive S), processed, and saved at regular intervals.<br><br>The file with the participant identification code is also stored on the server of the AG under password protection. Only the study director and employees authorized by the study director have access to this file. The access authorization is granted by the study director and expires after the end of the study participation.<br><br>All employees of the AG sign a form when they start work, whereby they undertake to comply with the department's internal guidelines regarding confidential information, confidentiality, and the protection of personal data. In particular, the unauthorized disclosure of data to third parties and the transfer of data worthy of protection to the to the outside world is prohibited.<br><br>The study participants are explicitly informed about the compliance with data protection, type and scope of the stored data and the right to their own data (incl. information, correction, data portability, restriction of processing, objection, and deletion).<br><br>The data and samples are passed on to the contract laboratory and the cooperation partners exclusively in pseudonymized form. A data transfer to other bodies is not intended.<br><br>The data will be retained as long as required by law and in accordance with good scientific practice (10 years) and subsequently deleted. Individual data sets can be deleted at the request of the study participant during the study (e.g., if consent to the processing of personal data is withdrawn). Deletion is understood to mean the final deletion of the respective individual data record and the removal of any security copies.<br><br>GCP monitoring is performed to verify the proper conduct of the study. The monitor appointed by the study director is given access to the study and patient files.
30. The names and addresses of the institutions involved in the study as study centers or study laboratories, as well as the study directors and the study physicians	**Study Center:**<br>NeuroCure Clinical Research Center (NCRC), Charité Universitätsmedizin Berlin, Charitéplatz 1, 10117 Berlin<br><br>**Principal Investigator:**<br>Prof. Dr. Dietmar Schmitz<br>[NeuroCure und Neurowissenschaftliches Forschungszentrum (NWFZ), Charité - Universitätsmedizin Berlin, Campus Mitte, Charitéplatz 1, 10117 Berlin]<br><br>**Co-Principal Investigator:**<br>Prof. Dr. med. Agnes Flöel<br>(Klinik und Poliklinik für Neurologie, Universitätsmedizin Greifswald, Ferdinand-Sauerbruch-Straße, 17475 Greifswald)<br><br>**Cooperation partners** (The study will take place exclusively in Berlin at the Charité)<br><br><u>BMBF partner:</u><br>Prof. Stephan J. Sigrist, Freie Universität Berlin, Institut für Biologie/Genetik, Takustrasse 6, 14195 Berlin und NeuroCure Charité -

	Universitätsmedizin Berlin, Campus Mitte, Charitéplatz 1, 10117 Berlin<br><br>Prof. Volker Haucke, Leibniz-Institut für Molekulare Pharmakologie im Forschungsverbund Berlin e.V. (FMP), Campus Berlin-Buch, Robert-Roessle-Str. 10, 13125 Berlin<br><br>Prof. Evgeni Ponimaskin, Institut für Neurophysiologie, OE4230 (I3, Raum 3100), Carl-Neuberg-Straße 1, 30625 Hannover<br><br>Prof. Frank Madeo, Alterung & Zelltod – Labor, Institut für Molekulare Biowissenschaften, Humboldtstrasse 50/EG, 8010 Graz, Österreich<br><br><u>Providing the polyamine and placebo capsules:</u><br>Prof. Frank Madeo, Alterung & Zelltod – Labor, Institut für Molekulare Biowissenschaften, Humboldtstrasse 50/EG, 8010 Graz, Österreich<br><br><u>Examination of blood samples:</u><br>Labor Berlin - Charité Vivantes GmbH, Laboratoriumsmedizin CVK, Sylter Straße 2, 13353 Berlin<br><br>Prof. Frank Madeo, Alterung & Zelltod – Labor, Institut für Molekulare Biowissenschaften, Humboldtstrasse 50/EG, 8010 Graz, Österreich<br><br>Prof. Dan Rujescu (im Rahmen der Analysen genetischer Polymorphismen), Universitätsklinik und Poliklinik für Psychiatrie, Psychotherapie und Psychosomatik, Julius-KühnStr.7, 06112 Halle/Saale<br><br>Prof. Stephan J. Sigrist, Freie Universität Berlin, Institut für Biologie/Genetik, Takustrasse 6, 14195 Berlin und NeuroCure Charité - Universitätsmedizin Berlin, Campus Mitte, Charitéplatz 1, 10117 Berlin<br><br>Prof. Dr. med. Agnes Flöel, Universitätsmedizin Greifswald, Klinik und Poliklinik für Neurologie, Ferdinand-Sauerbruch-Straße, 17475 Greifswald<br><br>Prof. Dr. Guido Kroemer, Centre de Recherche des Cordoliers, UMR1138 équipe 11, 15 Rue de l'École de Médecine, 75006 Paris, Frankreich<br><br><u>Hair sample analysis</u><br>Prof. Clemens Kirschbaum, Technische Universität Dresden, Lehrstuhl Biopsychologie, Zellescher Weg 19, 01069 Dresden<br><br><u>Implementation and evaluation of MRI/ PET</u><br>Prof. Holger Amthauer, Charité - Universitätsmedizin Berlin, Klinik für Nuklearmedizin CVK, Augustenburger Platz 1, 13353 Berlin (PET)<br><br>Prof. John-Dylan Haynes, Berlin Center for Advanced Neuroimaging, Campus Charité Mitte, Charité – Universitätsmedizin Berlin, Charitéplatz 1, 10117 Berlin (MRI)<br><br><u>Provision of the tracer (PET)</u><br>Piramal Imaging GmbH, Tegeler Straße 6-7, 13353 Berlin<br><br><u>Performance of the muscle biopsy and examination of the tissue samples</u><br>PD Dr. Knut Mai, Clinical Research Unit (CRU), Berlin Institute of Health (BIH), Charité Campus Mitte (CCM), Charitéplatz 1, 10117 Berlin (biopsy)

	Prof. Stephan J. Sigrist, Freie Universität Berlin, Institut für Biologie/Genetik, Takustrasse 6, 14195 Berlin und NeuroCure Charité - Universitätsmedizin Berlin, Campus Mitte, Charitéplatz 1, 10117 Berlin (analysis of tissue samples)<br><br>Prof. Frank Madeo, „Alterung & Zelltod“ – Labor, Institut für Molekulare Biowissenschaften, Humboldtstrasse 50/EG, 8010 Graz, Österreich (analysis of tissue samples)<br><br>Prof. Dr. Guido Kroemer, Centre de Recherche des Cordoliers, UMR1138 équipe 11, 15 Rue de l'École de Médecine, 75006 Paris, France (analysis of tissue samples)<br><br><u>Monitoring</u><br>NeuroCure Clinical Research Center (NCRC), Charité Universitätsmedizin Berlin, Charitéplatz 1, 10117 Berlin
31. Information on the suitability of the trial site, in particular on the adequacy of the resources and facilities available there and of the personnel available to conduct the clinical trial, and on experience in the conduct of similar trials	The study is funded by the BMBF. The study physicians are employed at the Charité. Sufficient examination rooms and laboratory space are available, as is the material required for testing. Through the project, scientific/medical doctoral students are employed to assist in the conduct and analysis of the studies. The study management and study physicians already have extensive experience in conducting similar studies.
32. Agreement on the access of the investigator/principal investigator/leader of the clinical trial, to the data and the principles on publication	The study data are published by the study management. The study results are published in an anonymous form that does not allow any conclusions to be drawn about the participant.
33. Information on the funding of the study:<br>Funding source (name and location)	Federal Ministry of Education and Research (BMBF) Hannoversche Strasse 28-30, 10115 Berlin

4

eTable 1. Amendments approved by the ethics committee of the Charité-Universitätsmedizin Berlin

**Amendment and Date of Votum**	**Changes**	**Reasons for Changes**
Amendment and Votum 28.09.2016	Replacement of potato starch in the placebo capsules with cellulose	Already in the preliminary study, cellulose was added to both the lyophilizate from wheat germ (polyamine capsules) and the potato starch (placebo capsules) to prevent "crumbling" of the extracts. To obtain a pure control preparation on the one hand and to reduce production costs on the other, the potato starch of the placebo capsules is now to be replaced by cellulose.
Amendment and Votum 28.11.2016	Participants will also have MRI measurements performed at the Berlin Center for Advanced Neuroimaging (BCAN)	Due to high utilization and possible capacity bottlenecks at the MRI/PET hybrid system the MRI measurements for participants who do not consent to PET will also be performed at the BCAN.
Amendment and Votum 07.02.2017	For all participants, MRI examinations should be performed at the BCAN<br><br>Additional visit for study participants who agree to PET examination, which can also be performed after the initial medical examination	Due to logistical and capacity limitations on the MRI/PET hybrid system and for reasons of more consistent data analysis, MRI measurements from all participants will be performed at the BCAN. PET measurements will be performed at the MRI/PET hybrid system. As PET examination only serves to characterize the study participants based on amyloid status, it may be performed with a time delay to the initial medical examination.
Amendment and Votum 06.09.2017	Small administrative changes (no additional/change in assessments)	The local PI changed from Dr. Flöel (now Co-PI) to Dr. Schmitz due to a change of the affiliation of Dr. Flöel. Passing on pseudonymized study and PET data to the tracer manufacturing company. Updating and addition of cooperation partners.
Amendment and Votum 27.03.2018	Concentration of polyamine capsules was updated<br><br>Optional hair sample analyses of cortisol	The presence of SCD symptoms has been associated with chronically elevated stress levels. Chronic stress has been discussed with an increased risk of chronic disease, e.g., Alzheimer's disease, and has negative effects on brain and cognitive functions in the elderly. Subgroup analyses will examine whether the effects of the polyamine intervention differ in patients with high and low cortisol levels.
Amendment and Votum 26.06.2018	Small administrative changes (no additional/change in assessments)	Adjustments in accordance with the requirements and conditions of the Data Protection Regulation (DSGVO) of the Data Protection Office (Charité Universitätsmedizin Berlin).

References

1. Gupta VK, Scheunemann L, Eisenberg T, et al. Restoring polyamines protects from age-induced memory impairment in an autophagy-dependent manner. Nat Neurosci 2013;16:1453-1460.

2. Minois N, Carmona-Gutierrez D, Madeo F. Polyamines in aging and disease. Aging (Albany NY) 2011;3:716-732.

3. Morrison LD, Becker L, Ang LC, Kish SJ. Polyamines in human brain: regional distribution and influence of aging. J Neurochem 1995;65:636-642.

4. Snitz BE, Lopez OL, McDade E, et al. Amyloid-beta Imaging in Older Adults Presenting to a Memory Clinic with Subjective Cognitive Decline: A Pilot Study. J Alzheimers Dis 2015;48 Suppl 1:S151-159.

5. Brown BM, Peiffer JJ, Taddei K, et al. Physical activity and amyloid-beta plasma and brain levels: results from the Australian Imaging, Biomarkers and Lifestyle Study of Ageing. Mol Psychiatry 2012.

6. Eisenberg T, Knauer H, Schauer A, et al. Induction of autophagy by spermidine promotes longevity. Nat Cell Biol 2009;11:1305-1314.

7. Wirth M, Madison CM, Rabinovici GD, Oh H, Landau SM, Jagust WJ. Alzheimer's Disease Neurodegenerative Biomarkers Are Associated with Decreased Cognitive Function but Not beta-Amyloid in Cognitively Normal Older Individuals. J Neurosci 2013;33:5553-5563.

8. La Joie R, Perrotin A, Barre L, et al. Region-specific hierarchy between atrophy, hypometabolism, and beta-amyloid (Abeta) load in Alzheimer's disease dementia. J Neurosci 2012;32:16265-16273.

9. Bickel H. Demenzsyndrom und Alzheimer Krankheit: Eine Schätzung des Krankenbestandes und der jährlichen Neuerkrankungen in Deutschland. Gesundheitswesen 2000;62:211-218.

10. Jessen F, Wolfsgruber S, Wiese B, et al. AD dementia risk in late MCI, in early MCI, and in subjective memory impairment. Alzheimers Dement 2014;10:76-83.

11. Scheef L, Spottke A, Daerr M, et al. Glucose metabolism, gray matter structure, and memory decline in subjective memory impairment. Neurology 2012;79:1332-1339.

12. Amariglio RE, Becker JA, Carmasin J, et al. Subjective cognitive complaints and amyloid burden in cognitively normal older individuals. Neuropsychologia 2012;50:2880-2886.

13. Jessen F, Feyen L, Freymann K, et al. Volume reduction of the entorhinal cortex in subjective memory impairment. Neurobiol Aging 2006;27:1751-1756.

14. Cherbuin N, Sargent-Cox K, Easteal S, Sachdev P, Anstey KJ. Hippocampal atrophy is associated with subjective memory decline: The PATH Through Life study. The American journal of geriatric psychiatry : official journal of the American Association for Geriatric Psychiatry 2015;23:446-455.

15. Mosconi L, De Santi S, Brys M, et al. Hypometabolism and altered cerebrospinal fluid markers in normal apolipoprotein E E4 carriers with subjective memory complaints. Biol Psychiatry 2008;63:609-618.

16. Erk S, Spottke A, Meisen A, Wagner M, Walter H, Jessen F. Evidence of neuronal compensation during episodic memory in subjective memory impairment. Arch Gen Psychiatry 2011;68:845-852.

17. Jack CR, Jr., Knopman DS, Jagust WJ, et al. Tracking pathophysiological processes in Alzheimer's disease: an updated hypothetical model of dynamic biomarkers. Lancet Neurol 2013;12:207-216.

18. Chen JJ, Rosas HD, Salat DH. Age-associated reductions in cerebral blood flow are independent from regional atrophy. Neuroimage 2011;55:468-478.

19. Abir M, Choi H, Cooke CR, Wang SC, Davis MM. Effect of a mass casualty incident: clinical outcomes and hospital charges for casualty patients versus concurrent inpatients. Academic emergency medicine : official journal of the Society for Academic Emergency Medicine 2012;19:280-286.

20. Tiboldi A, Lentini A, Provenzano B, et al. Hippocampal polyamine levels and transglutaminase activity are paralleling spatial memory retrieval in the C57BL/6J mouse. Hippocampus 2012;22:1068-1074.

21. Soda K, Kano Y, Sakuragi M, Takao K, Lefor A, Konishi F. Long-term oral polyamine intake increases blood polyamine concentrations. J Nutr Sci Vitaminol (Tokyo) 2009;55:361-366.

22. Schaeffer V, Lavenir I, Ozcelik S, Tolnay M, Winkler DT, Goedert M. Stimulation of autophagy reduces neurodegeneration in a mouse model of human tauopathy. Brain 2012;135:2169-2177.

23. Soda K, Dobashi Y, Kano Y, Tsujinaka S, Konishi F. Polyamine-rich food decreases ageassociated pathology and mortality in aged mice. Exp Gerontol 2009;44:727-732.

24. Gerner EW, Meyskens FL, Jr. Polyamines and cancer: old molecules, new understanding. Nat Rev Cancer 2004;4:781-792.

25. Russell D, Snyder SH. Amine synthesis in rapidly growing tissues: ornithine decarboxylase activity in regenerating rat liver, chick embryo, and various tumors. Proceedings of the National Academy of Sciences 1968;60:1420-1427.

26. Pegg AE, Feith DJ. Polyamines and neoplastic growth. Biochem Soc Trans 2007;35:295-299.

27. Welsh PA, Sass-Kuhn S, Prakashagowda C, McCloskey D, Feith D. Spermine synthase overexpression in vivo does not increase susceptibility to DMBA/TPA skin carcinogenesis or Min-Apc intestinal tumorigenesis. Cancer Biol Ther 2012;13:358-368.

28. Soda K, Kano Y, Chiba F, Koizumi K, Miyaki Y. Increased polyamine intake inhibits ageassociated alteration in global DNA methylation and 1,2-dimethylhydrazine-induced tumorigenesis. PLoS One 2013;8:e64357.

29. Ficker E, Taglialatela M, Wible BA, Henley CM, Brown AM. Spermine and spermidine as gating molecules for inward rectifier K+ channels. Science 1994;266:1068-1072.

30. Ransom RW, Stec NL. Cooperative Modulation of [3H]MK-801 Binding to the N-Methyl-d-Aspartate Receptor-Ion Channel Complex by l-Glutamate, Glycine, and Polyamines. J Neurochem 1988;51:830-836.

31. Oliver D, Baukrowitz T, Fakler B. Polyamines as gating molecules of inward-rectifier K+ channels. Eur J Biochem 2000;267:5824-5829.

32. Park MH, Igarashi K. Polyamines and Their Metabolites as Diagnostic Markers of Human Diseases. Biomol Ther (Seoul) 2013;21:1-9.

33. Bell MR, Belarde JA, Johnson HF, Aizenman CD. A neuroprotective role for polyamines in a Xenopus tadpole model of epilepsy. Nat Neurosci 2011;14:505-512.

34. Chen GG, Fiori LM, Moquin L, et al. Evidence of altered polyamine concentrations in cerebral cortex of suicide completers. Neuropsychopharmacology 2010;35:1477-1484.

35. Ferchmin PA, Perez D, Biello M. Spermine is neuroprotective against anoxia and N-methyl-D-aspartate in hippocampal slices. Brain Res 2000;859:273-279.

36. Kramer DL, Diegelman P, Jell J, Vujcic S, Merali S, Porter CW. Polyamine acetylation modulates polyamine metabolic flux, a prelude to broader metabolic consequences. J Biol Chem 2008;283:4241-4251.

37. Tersey SA, Colvin SC, Maier B, Mirmira RG. Protective effects of polyamine depletion in mouse models of type 1 diabetes: implications for therapy. Amino Acids 2014;46:633-642.

38. Pucciarelli S, Moreschini B, Micozzi D, et al. Spermidine and spermine are enriched in whole blood of nona/centenarians. Rejuvenation Res 2012;15:590-595.

39. Okamoto A, Sugi E, Koizumi Y, Yanagida F, Udaka S. Polyamine content of ordinary foodstuffs and various fermented foods. Biosci Biotechnol Biochem 1997;61:1582-1584.

40. Ibe S, Kumada K, Yoshida K, Otobe K. Natto (fermented soybean) extract extends the adult lifespan of Caenorhabditis elegans. Biosci Biotechnol Biochem 2013;77:392-394.

41. Zoumas-Morse C, Rock CL, Quintana EL, Neuhouser ML, Gerner EW, Meyskens FLJ. Development of a Polyamine Database for Assessing Dietary Intake. J Am Diet Assoc 2007;107:1024-1027.

42. Nishibori N, Fujihara S, Akatuki T. Amounts of polyamines in foods in Japan and intake by Japanese. Food Chem 2007;100:491-497.

43. Bardocz S, Duguid TJ, Brown DS, et al. The importance of dietary polyamines in cell regeneration and growth. Br J Nutr 1995;73:819-828.

44. Ralph A, Englyst K, Bardócz S. Polyamine content of the human diet. In: Bardócz S, White A, eds. Polyamines in Health and Nutrition. Londin, UK: Kluwer Academic Publishers, 1999: 123-137.

45. Ali MA, Poortvliet E, Strömberg R, Yngve A. Polyamines: total daily intake in adolescents compared to the intake estimated from the Swedish Nutrition Recommendations Objectified (SNO). Food Nutr Res 2011;55:10.3402/fnr.v3455i3400.5455.

46. Til HP, Falke HE, Prinsen MK, Willems MI. Acute and subacute toxicity of tyramine, spermidine, spermine, putrescine and cadaverine in rats. Food Chem Toxicol 1997;35:337-348.

47. Ishida S, Sarada M, Seki H, McGirr L, Lau A, Morishita K. Genotoxicity and subchronic oral toxicity of L-ornithine monohydrochloride. Regul Toxicol Pharmacol 2013;67:360-371.

48. Pegg AE. Mammalian polyamine metabolism and function. IUBMB Life 2009;61:880-894.

49. Yan DH, Nishimura K, Yoshida K, et al. Different intracellular polyamine concentrations underlie the difference in the inward rectifier K(+) currents in atria and ventricles of the guinea-pig heart. J Physiol 2005;563:713-724.

50. Pegg AE, Wang X. Mouse models to investigate the function of spermine. Commun Integr Biol 2009;2:271-274.

51. Vivo M, de Vera N, Cortes R, Mengod G, Camon L, Martinez E. Polyamines in the basal ganglia of human brain. Influence of aging and degenerative movement disorders. Neurosci Lett 2001;304:107-111.

52. Vanrell MC, Cueto JA, Barclay JJ, et al. Polyamine depletion inhibits the autophagic response modulating Trypanosoma cruzi infectivity. Autophagy 2013;9:1080-1093.

53. Eisenberg T, Abdellatif M, Schroeder S, et al. Cardioprotection and lifespan extensionby the natural polyamine spermidine. Nat Med 2016 (in revision).

54. Feng Y, He D, Yao Z, Klionsky DJ. The machinery of macroautophagy. Cell Res 2014;24:24-41.

55. Eisenberg T, Schroeder S, Andryushkova A, et al. Nucleocytosolic depletion of the energy metabolite acetyl-coenzyme a stimulates autophagy and prolongs lifespan. Cell Metab 2014;19:431-444.

56. Woo T, Yu S, Visvanathan R. Systematic Literature Review on the Relationship Between Biomarkers of Sarcopenia and Quality of Life in Older People. J Frailty Aging 2016;5:88-99.

57. Smith JC, Nielson KA, Woodard JL, et al. Physical activity reduces hippocampal atrophy in elders at genetic risk for Alzheimer's disease. Front Aging Neurosci 2014;6:61.

58. Witte AV, Jansen S, Schirmacher A, Young P, Floel A. COMT Val158Met Polymorphism Modulates Cognitive Effects of Dietary Intervention. Front Aging Neurosci 2010;2:146.

59. Kim JM, Stewart R, Bae KY, et al. Role of BDNF val66met polymorphism on the association between physical activity and incident dementia. Neurobiol Aging 2011;32:551 e555-512.

60. Madeo F, Zimmermann A, Maiuri MC, Kroemer G. Essential role for autophagy in life span extension. J Clin Invest 2015;125:85-93.

61. Nilsson P, Loganathan K, Sekiguchi M, et al. Abeta secretion and plaque formation depend on autophagy. Cell Rep 2013;5:61-69.

62. Luo J, Mohammed I, Warmlander SK, Hiruma Y, Graslund A, Abrahams JP. Endogenous polyamines reduce the toxicity of soluble abeta peptide aggregates associated with Alzheimer's disease. Biomacromolecules 2014;15:1985-1991.

63. Eckerström M, Berg AI, Nordlund A, Rolstad S, Sacuiu S, Wallin A. High Prevalence of Stress and Low Prevalence of Alzheimer Disease CSF Biomarkers in a Clinical Sample with Subjective Cognitive Impairment. Dement Geriatr Cogn Disord 2016;42:93-105.

64. Machado A, Herrera Antonio J, de Pablos Rocío M, et al. Chronic stress as a risk factor for Alzheimer's disease. Rev Neurosci 2014: 785.

65. Lupien SJ, de Leon M, de Santi S, et al. Cortisol levels during human aging predict hippocampal atrophy and memory deficits. Nat Neurosci 1998;1:69.

66. Wright KD, Hickman R, Laudenslager ML. Hair Cortisol Analysis: A Promising Biomarker of HPA Activation in Older Adults. Gerontologist 2015;55:S140-S145.

67. Folstein M, Folstein S, McHugh P. “Mini-mental state”: a practical method for grading the cognitive state of patients for the clinicians. J Psychiatr Res 1975;12:189-198.

68. Wechsler D. Wechsler Memory Scale - Third edition. Manual. San Antonio, TX: The Psychological Corperation, 1997.

69. Lezak M. Neuropsychological assessment. New York, Oxford: Oxford University Press, 2004.

70. Yesavage JA, Brink TL, Rose TL, et al. Development and validation of a geriatric depression screening scale: a preliminary report. J Psychiatr Res 1982;17:37-49.

71. Lezak MD. Neuropsychological assessment, 4 ed: New York, Oxford: Oxford University Press, 2004.

72. Baddeley AD, Wilson B, Watts F. Handbook of Memory Disorders: Chichester, W.Sussex: John Wiley & Sons Ltd., 1995.

73. Duzel E, Schutze H, Yonelinas AP, Heinze HJ. Functional phenotyping of successful aging in long-term memory: Preserved performance in the absence of neural compensation. Hippocampus 2011;21:803-814.

74. Stark SM, Yassa MA, Lacy JW, Stark CE. A task to assess behavioral pattern separation (BPS) in humans: Data from healthy aging and mild cognitive impairment. Neuropsychologia 2013;51:2442-2449.

75. Härting C, Markowitsch H, Neufeld H, Calabrese P, Dejerine J, Deisinger K. Wechsler Gedächtnistests - revidierte Fassung (WMS-R). Bern: Huber, 2000.

76. Kessels RP, van Zandvoort MJ, Postma A, Kappelle LJ, de Haan EH. The Corsi Block-Tapping Task: standardization and normative data. Appl Neuropsychol 2000;7:252-258.

77. Reitan RM, Herring S. A short screening device for identification of cerebral dysfunction in children. J Clin Psychol 1985;41:643-650.

78. Wechsler D. Wechsler Adult Intelligence Scale-III. San Antonio, TX: The Psychological Corporation, 1997.

79. Trenerry MR, Crosson B, DeBoe J, Leber WR. Stroop Neuropsychological Screening Test manual. Odessa, FL: Psychological Assessment Resources, 1989.

80. Zimmermann P, Fimm B. Testbatterie zur Aufmerksamkeitsprüfung (TAP). Freiburg: Psytes, 1993.

81. Buysse DJ, Reynolds CF, 3rd, Monk TH, Berman SR, Kupfer DJ. The Pittsburgh Sleep Quality Index: a new instrument for psychiatric practice and research. Psychiatry research 1989;28:193-213.

82. Frey I, Berg A, Grathwohl D, Keul J. [Freiburg Questionnaire of physical activity--development, evaluation and application]. Soz Praventivmed 1999;44:55-64.

83. Watson D, Clark LA, Carey G. Positive and negative affectivity and their relation to anxiety and depressive disorders. J Abnorm Psychol 1988;97:346-353.

84. Yao Jk, Stanley JA, Reddy RD, Keshavan MS, Pettegrew JW. Correlations between peripheral polyunsaturated fatty acid content and in vivo membrane phospholipid metabolites. Biol Psychiatry 2002;52:823-830.

85. Kohlmeier L. Future of dietary exposure assessment. Am J Clin Nutr 1995;61:702S-709S-702S-709S.

86. Recommendations of the International Commission on Radiological Protection. ICRP Publication 60. Oxford: International Commission on Radiological Protection ICRP, 1991.

87. Champely S, Ekstrom C, Dalgaard P, Gill J, Wunder J, De Rosario H. pwr: Basic Functions for Power Analysis. R package. CRAN 2015.

Statistical Analysis Plan (SAP)

"Effects of Spermidine Supplementation on Cognition and Biomarkers in Older Adults with Subjective Cognitive Decline", Acronym: SmartAge

Revised Version *1.1 (18.02.2022)*

**Revision:**	As part of the revision, the effect size used to calculate the sample size was corrected from Cohen's d = 0.5 to 0.6.
**Intervention therapy:**	The intervention will be spermidine supplement administered daily in the form of six capsules, each containing 125 mg extract, resulting in a daily dose of 750 mg extract or 0.9 mg spermidine, 0.5 mg spermine, 0.2 mg putrescine, < 0.004 mg of cadaverine, and 0.12 mg of L-ornithine.
**Control therapy:**	Placebo capsules (containing microcrystalline cellulose); all capsules have identical appearance.
**Study population:**	Older individuals with subjective cognitive decline (SCD)
**Funding:**	Funding for this trial is provided by the Bundesministerium für Bildung und Forschung (SMARTAGE, FKZ 01GQ1420B), the Hans Gerhard Creutzfeldt scholarship (FKZ CSB II, 01EO1301 TP T2), and the Deutsche Forschungsgemeinschaft (DFG, EXC 257 NeuroCure). For the applied PET measurements, the approved ligand FBB (Neuraceq™) is provided free of charge by Life Molecular Imaging (LMI). Frank Madeo is grateful to BioTechMed-Graz for the flagship project "EPIAge", to the Austrian Science Fund FWF (Austria) for grants, P29262, P29203, P27893, and SFB Lipotox' (F3012), as well as to BMWFW and the Karl-Franzens University for grants 'Unkonventionelle Forschung' and flysleep (80.109/0001 – WF/V/3b/2015). Sponsor: Charité – Universitätsmedizin Berlin, Germany.
**Clinical Phase:**	Monocentric, double-blind Phase IIb trial
**Clinicaltrials.gov Identifier:**	NCT03094546

Approved by

Dietmar Schmitz, Principal Investigator	Berlin, 22.2.2022<br>Place, Date, Signature
Agnes Flöel, Co-Principal Investigator	Greifswald, 18.2.2022<br>Place, Date, Signature
Claudia Schwarz, Study coordinator	Helsinki,18.02.2022<br>Place, Date, Signature
Ulrike Grittner, Statistician	Ulrike Grittner Digital unterschrieben von Ulrike Grittner Datum: 2022.02.22 16:45:19 +01'00'<br>Place, Date, Signature
Ralph Schilling, Deputy Statistician	Ralph Schilling Digital unterschrieben von Ralph Schilling DN: cn=Ralph Schilling, o, ou, email=ralph.schilling@charite.de, c=DE Datum: 2022.02.22 15:40:09 +01'00'<br>Place, Date, Signature

Content

1 Background 4
1.1 Study Objective 4
1.2 Primary Hypothesis 4
1.3 Secondary Hypotheses 4
1.4 Study Design 5
1.5 Sample Size Calculation 5
2 Analysis Sets 5
2.1 Definitions 5
2.2 Application 6
3 Trial Centres 6
3.1 Recruitment 6
4 Analysis Variables and Time Points of Assessment 7
4.1 Baseline Characteristics 11
4.2 Primary Outcome 11
4.3 Secondary Outcomes 11
4.4 Safety Outcomes 12
5 Handling of Missing Values 13
6 Statistical Analyses 13
6.1 Primary Analysis 13
6.2 Secondary Analyses 13
6.3 Safety/Tolerability 14
6.4 Planned subgroup analyses 14
7 References 15

1 Background

Given the global increase in the aging population and age-related diseases, the promotion of healthy aging is one of the most crucial public health issues. This trial aims to contribute to the establishment of effective approaches to promote cognitive and brain health in older individuals with subjective cognitive decline (SCD) (see Wirth and colleagues[1] for study protocol). Presence of SCD is known to increase the risk of objective cognitive decline and progression to dementia due to Alzheimer's disease[2-5]. Therefore, it is our primary goal to determine whether spermidine supplementation has a positive impact on memory performance in this at-risk group, as compared with placebo. The secondary goal is to examine the effects of spermidine intake on other neuropsychological, behavioral, and physiological parameters. This SAP was prepared in accordance with the Guidelines for the Content of Statistical Analysis Plans in Clinical Trials[6].

1.1 Study Objective

The planned analyses will assess the impact of spermidine supplementation on cognitive and brain health in older individuals with subjective cognitive decline (SCD). In addition, we will identify possible mechanisms of action underlying the anticipated cognitive benefits. Overall, this trial will contribute to the possible establishment of nutrition intervention in the prevention of Alzheimer's disease.

1.2 Primary Hypothesis

The primary hypothesis of this trial is that spermidine supplementation has a beneficial impact on memory performance in cognitively unimpaired older individuals with SCD at the end of the 12-month intervention (V2), as compared with placebo.

1.3 Secondary Hypotheses

We hypothesize favorable developments in the intervention group compared to the control group in the following domains (for details see section *4.3. Secondary Outcomes*):

- in memory performance (operationalized by mnemonic discrimination performance) between baseline (V1) and follow-up assessment (V3, 18 months after baseline)
- in neuropsychological parameters
- in behavioral parameters and lifestyle
- in quality of life
- in physiological parameters
- in subjective cognitive function, cardiovascular risk, as well as muscle function/strength on an exploratory basis

1.4 Study Design

This is a monocentric, randomized, double-blind, placebo-controlled Phase IIb trial, carried out at the NeuroCure Clinical Research Center, Charité – Universitätsmedizin Berlin.
The trial includes 12 months of intervention with spermidine supplementation (target intervention) compared with 12 months of placebo intake (control intervention). The trial will compare outcomes of the two intervention groups, with participants randomized to one of the two study arms. Participants will be randomly assigned to either spermidine group or placebo group. A blockwise (block size of 6) randomization sequence, stratified by age (60-70, 70-80, and 80-90 years) and sex, will be generated using a computer-based algorithm (http://www.randomization.com/). Participant allocation will be performed at a 1:1 ratio by a study investigator without involvement in outcome assessments.
This study will be a placebo-controlled trial, using microcrystalline cellulose as a comparator condition. Our placebo capsules will be identical to the verum intervention in shape, color, taste, and smell, but contain no active ingredients. The World Health Organization (WHO) and the U.S. Food and Drug Administration (*FDA*) recognize that the use of cellulose as a food additive is safe and well tolerated in animal models and humans.
The SmartAge trial has been approved by the responsible Institutional Review Board and will be carried out in compliance with institutional ethical standards and the Declaration of Helsinki (see section: Ethics approval).

1.5 Sample Size Calculation

Power calculation was conducted to estimate the number of participants required to detect a group difference in the primary outcome (i.e., mnemonic discrimination performance) at the end of the intervention. Sample size estimation was based on behavioral data obtained in a Phase IIa pilot trial[7]. In this pre-study including 28 SCD participants and a 3-month intervention period, we determined an effect size of Cohen's d = 0.65 on the group difference in change of mnemonic discrimination performance between baseline (V1) and the end of intervention (V2). Since this is a first estimate of the effect size based on a small sample, we based our sample size estimation for the present Phase IIb trial on a smaller effect size of Cohen's d = 0.6. To demonstrate a significant effect in the primary outcome, 50 participants per group (including a 10% drop-out rate) need to be included in the analysis with an unpaired-sample t-test (two-sided significance level = 0.05, power: 80%). Sample size estimation was conducted using a conservative approach[8] based on an unpaired-sample t-test, even though the intended analysis of the primary outcome will be performed using ANCOVA models (see section: statistical analysis). Case number estimation was compiled with the R package "pwr"[9].

2 Analysis Sets

2.1 Definitions

The full analysis set comprises all subjects who were randomised. Randomization is performed block-wise with a 1:1 allocation ratio. In case of missing values, multiple imputation methods will be used to estimate missing values. The per protocol analysis set comprises all subjects who received intervention

or control medication for at least 12 months. The safety analysis set comprises all patients who received at least one dosage of intervention or control medication.

2.2 Application

The primary efficacy analysis will be done using the full analysis set including estimated values from multiple imputations for missing values (Intention to treat). An analysis of the primary outcome in the per protocol analysis set will be used as sensitivity analysis. The safety analysis will be done using the safety analysis set and comparing patients according to the actual medication they received.

3 Trial Centres

Patients were recruited at the NeuroCure Clinical Research Center, Charité – Universitätsmedizin Berlin.

3.1 Recruitment

Participants were recruited from healthcare facilities as well as through advertisements from the general German population. Telephone screenings for study eligibility were conducted as part of the study enrollment with all potential participants. Criteria of SCD have been assessed in accordance with the framework of Jessen and colleagues[10]. Eligible candidates were invited for on-site screening, followed by a baseline assessment (V1) if the screening met our inclusion and exclusion criteria (i.e. normal cognitive performance).

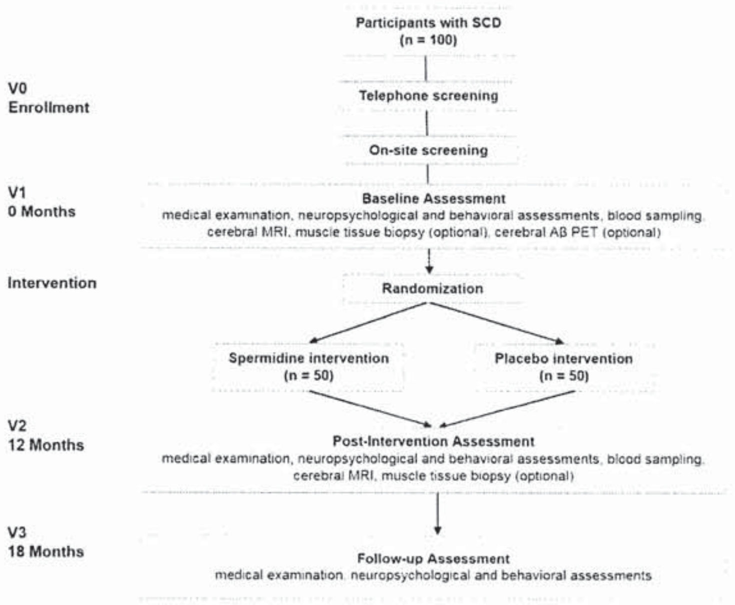

Figure 1 SmartAge study flowchart. Abbreviations: MRI, magnetic resonance imaging; PET, positron emission tomography, SCD, subjective cognitive decline. Obtained from Wirth and colleagues[1].

4 Analysis Variables and Time Points of Assessment

Table 1 SmartAge study outcome assessment
Adapted from SmartAge study protocol (Wirth et al., 2019)

Study procedure	Screening		Baseline	Double-blind period		
Visit number	V0 phone	V0 clinic	V1 clinic	Intervention	V2 clinic	V3 clinic
Study month	0	0	0	1 - 12	12	18
Enrollment /Eligibility screening						
Inclusion/exclusion criteria	X	X				
Suitability MRI/PET	X				(X)	
Suitability muscle biopsy	X	X			(X)	
Assessment of presence of SCD incl. worries	X					
Enrollment / Screening assessment						
Medical assessment		X	X		X	X
Mini-Mental State Examination (MMSE)[11]		X			X	X
Logical Memory II subscale[12]		X			X	X
Trail Making Test A (TMT)[13]		X			X	X
Geriatric Depression Scale (GDS)[14,15]		X			X	X
Instrumental Activities of Daily Living Scale (IADL)[16]		X			X	X
Informed consent		X				
Allocation/randomization			X			
Intervention/ Baseline variables						
Demographics (age, civil status, education)	X	X			(X)	(X)
Family history (Dementia, Parkinson disease, and stroke)		X			(X)	(X)

Study procedure	Screening		Baseline	Double-blind period		
Visit number	V0 phone	V0 clinic	V1 clinic	Intervention	V2 clinic	V3 clinic
Everyday Cognition Scales (ECog-39)[17]			X			
Lifetime Experience Questionnaire (LEQ)[18]			X			
Cognitive Activity Interview (CAI)[19]			X			
Big Five Inventory-10 (BFI-10)[20]			X			
Stress Coping Style Questionnaire (Stressverarbeitungsfragebogen [SVF-78])[21]			X			
Oldfield Hand Preference[22]			X			
Multiple-Choice Vocabulary Intelligence Test (Mehrfachwahl-Wortschatztest [MWT])[23]			X			
APOE ε4 status			X			
Aβ status (cerebral PET, optional consent)			X			
Intervention/ Primary outcome						
Mnemonic Similarity Task (MST)[24]			X		X	X
Intervention/ Secondary outcome						
Auditory Verbal Learning Test (Verbaler Lern-und Merkfähigkeitstest [VLMT])[25]			X		X	X
Doors and People Test[26]			X		X	X
Digit Symbol[27]			X		X	X
Trail Making Test B (TMT)[13]			X		X	X
Block Tapping Test[28]			X		X	X
Stroop Test[29]			X		X	X

Study procedure	Screening		Baseline	Double-blind period		
Visit number	V0 phone	V0 clinic	V1 clinic	Intervention	V2 clinic	V3 clinic
Digit Span[27]			X		X	X
Test of Attentional Performance (Testbatterie zur Aufmerksamkeitsprüfung [TAP])[30] Subtests: alertness and divided attention			X		X	
Semantic/Phonemic Fluency[31]			X		X	X
Boston Naming Test[31]			X		X	X
Lifestyle						
Freiburg questionnaire on physical activity (Freiburger Fragebogen zur körperlichen Aktivität [FKA])[32]			X		X	X
CAI Present[19]			X		X	X
Pittsburgh Sleep Quality Index (PSQI)[33]			X		X	X
Food Frequency Questionnaire (FFQ)[34,35]			X		X	X
Mediterranean Diet Adherence Screener (MEDAS)[36,37]			X		X	X
Food Frequency List (FFL)[38,39]			X		X	X
Penn State Worry Questionnaire (PSWQ)[40,41]			X		X	X
Response Styles Questionnaire - Deutsche Version (RSQ-D)[42]			X		X	X
State-Trait Anxiety Inventory (STAI-G)[43]			X		X	X
Short Form Health Survey (SF-12)[44]			X		X	X

Study procedure	Screening		Baseline	Double-blind period		
Visit number	V0 phone	V0 clinic	V1 clinic	Intervention	V2 clinic	V3 clinic
World Health Organization Quality of Life (WHOQOL-BREF)[45]			X		X	X
Autophagy markers from muscle biopsy (optional consent) (e.g., proteomics, metabolomics)[46-48]			X		X	
Blood-based markers (e.g., polyamine levels[34], metabolomics, proinflammatory biomarkers)			X		X	
Cerebral neuroimaging markers (i.e. brain structure, perfusion, function)			X		X	
Additional outcomes						
ECog-39, adapted[17]			X		X	X
Meta Memory Questionnaire (MMQ), adapted[49]			X		X	X
Cardiovascular risk factors (e.g., blood pressure)			X		X	X
Short Physical Performance Battery (SPPB) (optional consented to muscle biopsy)[50]			X		X	X
Handgrip strength (optional consented to muscle biopsy)			X		X	X
Compliance to intervention						
Capsule count				X	X	
Self-reported compliance				X	X	

Abbreviations: Aβ, β-amyloid; APOE, apolipoprotein E; MRI, magnetic resonance imaging; PET, positron emission tomography.

4.1 Baseline Characteristics

Assessments of participants' characteristics will be conducted at baseline, including among others the following parameters (also summarized in Table 2, see below): (a) demographic information including age and education; (b) information on family history focused on AD; and (c) behavioral measures of subjective cognitive function, lifestyle, and personality traits. In addition, (d) physiological measures of brain Aβ status, measured using [18F] florbetaben (FBB) PET, and genotype information on apolipoprotein E (APOE) ε4 status, measured using genotyping of blood-derived deoxyribonucleic acid (DNA), will be obtained. Potential changes in demographic information and family history will be recorded throughout the trial.

4.2 Primary Outcome

The primary endpoint of this trial is the change in memory performance between baseline visit (V1) and post-intervention visit (V2). Memory performance is operationalized by mnemonic discrimination ability, to be assessed by the Mnemonic Similarity Task (MST).

4.3 Secondary Outcomes

Secondary endpoints are:

a) Neuropsychological parameters on verbal and visual-spatial memory, attention, executive functions, and sensorimotor speed, assessed at V1, V2, and V3
   - German version of the Auditory Verbal Learning Test (Verbaler Lern- und Merkfähigkeitstest [VLMT][25]), Doors and People Test[26], Digit Symbol[27], Trail Making Test B (TMT)[13], Block Tapping Test[28], Stroop Test[29], Digit Span[27], Test of Attentional Performance (Testbatterie zur Aufmerksamkeitsprüfung [TAP][30]) including the subtests alertness and divided attention, semantic/phonemic fluency[31], and Boston Naming Test[31]

b) Behavioral parameters of lifestyle behaviors, psycho-affective characterization, and perceived quality of life, assessed at V1, V2, and V3
   - Freiburg questionnaire on physical activity (Freiburger Fragebogen zur körperlichen Aktivität [FKA][32]), CAI Present[19], Pittsburgh Sleep Quality Index (PSQI)[33], Food Frequency Questionnaire (FFQ)[34,35], Mediterranean Diet Adherence Screener (MEDAS)[36,37], Penn State Worry Questionnaire (PSWQ)[40,41], Response Styles Questionnaire - German version (RSQ-D)[42], State-Trait Anxiety Inventory (STAI-G)[43], Short Form Health Survey (SF-12)[44], and World Health Organization Quality of Life (WHOQOL-BREF)[45]

c) Physiological parameters including autophagy signaling (measured in muscle biopsies), peripheral vascular parameters (measured in blood), and parameters of brain structure, perfusion, and function (measured using cerebral magnetic resonance imaging [MRI]) to be assessed at V1 and V2

- Autophagy markers from muscle biopsy (e.g., proteomics, metabolomics)[46-48]
- Blood-based markers (e.g., polyamine levels[34], metabolomics, proinflammatory biomarkers)
- Cerebral neuroimaging markers (i.e. brain structure, perfusion, function)

d) Outcomes of subjective cognitive function, cardiovascular risk factors, as well as muscle function and strength markers (available for a sub-sample)
   - Everyday Cognition Scales-39 (ECog-39)[17], systolic/diastolic blood pressure, heart rate, Short Physical Performance Battery (SPPB)[50], and Handgrip strength

4.4 Safety Outcomes

The following measures will be used as safety outcomes, between inclusion of the patient in the trial and 18 months after baseline (V3):

Severe adverse events (SAEs): monitored continuously throughout the trial, and recorded at baseline (V1), during intervention (3, 6, and 9 months), 12-month visit at the end of intervention (V2), 18-month visit (V3):

- fatal or life-threatening events
- referral to an acute hospital as well as inpatient hospital treatment or its extension
- events that result in a permanent and/or significant disability or in incapacity for work
- events that lead to a congenital anomaly/birth defect
- diagnosis of a malignant/neoplastic processes

Exceptions to the SAE definition:

- inpatient hospital stays, which are already planned before study enrollment or occurred during the screening phase and therefore are not associated with the study intervention
- inpatient hospital stays within the scope of rehabilitation measures

Adverse events (AEs): monitored continuously throughout the trial, and recorded at baseline (V1), during intervention (3, 6, and 9 months), 12-month visit at the end of intervention (V2), 18-month visit (V3):

- occurrence/deterioration of signs of disease or symptoms that occur after inclusion of the subject in the study, whether or not it is causally related to participation in the study
  - including among others gastrointestinal symptoms, burping, diarrhea

Both AEs and SAEs will be grouped in classes and summarized including the following parameters: incidence of (S)AEs, intensity (mild, moderate, and severe), and relationship to intervention (not related, unlikely related, possibly related, and related).

5 Handling of Missing Values

In case of missing values and under the assumption of missing at random (MAR) or missing completely at random (MCAR) as missing data mechanism, data will be estimated using multiple imputation methods with 30 imputed data sets. To estimate values in a realistic range and with values similar as in complete cases, we will use predictive mean matching as this method uses original values of participants without missings for the estimation procedure.

6 Statistical Analyses

For all analyses (including analysis of primary outcome) appropriate descriptive statistics (mean, standard deviation, median, interquartile range, absolute and relative frequencies) depending on the scale and distribution of the outcome variable will be presented.

Statistical analyses will be divided to analyze

1. immediate treatment effects by including all measures until include V2 (post intervention assessment)
2. long-term treatment effects by focusing on V3 (6 months follow up)

6.1 Primary Analysis

Statistical analysis will be performed using an ANCOVA model with the change in memory performance (Mnemonic Similarity Task, MST) (V2–V1) as dependent variable, intervention group as independent variable, and baseline memory performance (MST) as well as sex and age (stratification variables from stratified randomisation as co-variates). The primary analysis will be carried out in the full analysis set including estimated values in case of missings (multiple imputed data sets).

6.2 Secondary Analyses

Immediate treatment effects

Performance on secondary outcomes will be analyzed in the same manner as the primary outcome, using separate ANCOVA models for change in outcome from baseline to V2 as dependent variable for each endpoint, intervention group as factor, and particular baseline measure as well as age and sex as covariates. Interaction terms and additional covariates will be included to adjust for possible confounders or to test subgroup differences. Type of link function will depend on the scaling of the dependent variable.

Long-term treatment effects

Long term treatment effects will be analyzed using separate mixed models over the whole study period for each outcome. These models will include the baseline measure of the particular outcome, age and sex as covariates and a random effect for the participant (random intercept). Type of link

function (logistic, linear, ordinal) will depend on the scaling of the dependent variable. In case of skewed continuous data, variables will be transformed before analysis.

All secondary analyses will be done using the full analysis set with multiple imputed data in case of missing values. Per protocol analyses will be done as sensitivity analyses. All secondary analyses will be done in an exploratory framework.

6.3 Safety/Tolerability

Safety outcomes will be reported separately as incidences (n, incidence rate with 95%CI) in total and by intervention group based on the safety analysis set. Patients will be grouped according to their actually received treatment. Incidence rates and 95%CI will be based on poisson regression models that account for the different observation periods for each patient. Group comparisons will be done using incidence rate ratios and 95%CI. Results of safety analysis will be interpreted and discussed thoroughly also for minor group differences, since statistically significance is not of importance here.

6.4 Planned Subgroup Analyses

Potential differences in the efficacy of the intervention on primary and secondary outcomes will be analyzed among the following pre-specified subgroups at time points V1, V2 and V3 (subgroups defined by using baseline information):

- based on dichotomization with regard to age (<70 and ≥70 years)
- based on dichotomization with regard to sex (male vs. female)
- based on dichotomization with regard to genetic phenotype (e.g., APOE ε4 positive vs. APOE ε4 negative)
- based on defined categories of severity of subjective cognitive complaints using the ECog-39 (e.g., median split or defined categories)
- based on defined categories of dietary spermidine intake using the Food Frequency Questionnaire (e.g., median split or defined categories)
- based on defined categories of memory function using the MST (e.g., median split or defined categories)

All subgroup analyses will be done within an exploratory framework. Moreover, to evaluate the complex relationship between several variables, such as primary/secondary outcomes, intervention group and cerebral amyloid-β status or genetic phenotype, we also plan to conduct structural equation modelling.

7 References

1. Wirth M, Schwarz C, Benson G, et al. Effects of spermidine supplementation on cognition and biomarkers in older adults with subjective cognitive decline (SmartAge)—study protocol for a randomized controlled trial. *Alzheimer's research & therapy.* 2019;11(1):36.
2. Jessen F, Wolfsgruber S, Wiese B, et al. AD dementia risk in late MCI, in early MCI, and in subjective memory impairment. *Alzheimer's & Dementia.* 2014;10(1):76-83.
3. Mitchell A, Beaumont H, Ferguson D, Yadegarfar M, Stubbs B. Risk of dementia and mild cognitive impairment in older people with subjective memory complaints: meta-analysis. *Acta Psychiatrica Scandinavica.* 2014;130(6):439-451.
4. van Harten AC, Mielke MM, Swenson-Dravis DM, et al. Subjective cognitive decline and risk of MCI: the Mayo Clinic Study of Aging. *Neurology.* 2018;91(4):e300-e312.
5. Snitz BE, Wang T, Cloonan YK, et al. Risk of progression from subjective cognitive decline to mild cognitive impairment: The role of study setting. *Alzheimer's & Dementia.* 2018;14(6):734-742.
6. Gamble C, Krishan A, Stocken D, et al. Guidelines for the content of statistical analysis plans in clinical trials. *Jama.* 2017;318(23):2337-2343.
7. Wirth M, Benson G, Schwarz C, et al. The effect of spermidine on memory performance in older adults at risk for dementia: a randomized controlled trial. *Cortex.* 2018;109:181-188.
8. Borm GF, Fransen J, Lemmens WA. A simple sample size formula for analysis of covariance in randomized clinical trials. *Journal of clinical epidemiology.* 2007;60(12):1234-1238.
9. Champely S, Ekstrom C, Dalgaard P, Gill J, Wunder J, Rosario H. pwr: basic functions for power analysis v. 1.1. 3. *Computer software] Retrieved from http://cran r-project org/package= pwr.* 2015.
10. Jessen F, Amariglio RE, van Boxtel M, et al. A conceptual framework for research on subjective cognitive decline in preclinical Alzheimer's disease. *Alzheimer's & dementia.* 2014:1-9.
11. Folstein MF, Folstein SE, McHugh PR. "Mini-mental state". A practical method for grading the cognitive state of patients for the clinician. *J Psychiatr Res.* 1975;12(3):189-198.
12. Wechsler D. *WMS-R : Wechsler Memory Scale--Revised : manual.* San Antonio, TX: Psychological Corporation/Harcourt Brace Jovanovich; 1987.
13. Reitan RM. Validity of the trailmaking test as an indication of organic brain damage. *Percept Mot Skills.* 1955;8:271-276.
14. Yesavage JA, Brink TL, Rose TL, et al. Development and validation of a geriatric depression screening scale: a preliminary report. *Journal of psychiatric research.* 1982;17(1):37-49.
15. Gauggel S, Birkner B. Validity and reliability of a German version of the Geriatric Depression Scale (GDS). *Zeitschrift fur Klinische Psychologie-Forschung und Praxis.* 1999;28(1):18-27.
16. Lawton MP, Brody EM. Assessment of older people: self-maintaining and instrumental activities of daily living. *The gerontologist.* 1969;9(3_Part_1):179-186.
17. Farias ST, Mungas D, Reed BR, et al. The measurement of everyday cognition (ECog): scale development and psychometric properties. *Neuropsychology.* 2008;22(4):531.
18. Valenzuela MJ, Sachdev P. Assessment of complex mental activity across the lifespan: development of the Lifetime of Experiences Questionnaire (LEQ). *Psychological medicine.* 2007;37(7):1015-1025.
19. Wilson R, Barnes L, Bennett D. Assessment of lifetime participation in cognitively stimulating activities. *J Clin Exp Neuropsychol.* 2003;25(5):634-642.
20. Rammstedt B, Kemper CJ, Klein MC, Beierlein C, Kovaleva A. Eine kurze Skala zur Messung der fünf Dimensionen der Persönlichkeit: Big-Five-Inventory-10 (BFI-10). 2012.
21. Janke W, Erdmann G. *SVF 78: eine Kurzform des Stressverarbeitungsfragebogens SVF 120; Kurzbeschreibung und grundlegende Kennwerte; Manual.* Hogrefe, Verlag für Psychologie; 2002.
22. Oldfield RC. The assessment and analysis of handedness: the Edinburgh inventory. *Neuropsychologia.* 1971;9(1):97-113.
23. Hessler J, Jahn T, Kurz A, Bickel H. The MWT-B as an estimator of premorbid intelligence in MCI and dementia. *Zeitschrift für Neuropsychologie.* 2013.
24. Stark SM, Yassa MA, Lacy JW, Stark CE. A task to assess behavioral pattern separation (BPS) in humans: Data from healthy aging and mild cognitive impairment. *Neuropsychologia.* 2013;51(12):2442-2449.
25. Helmstaedter C, Lendt M, Lux S. *Verbaler Lern- und Merkfähigkeitstest* Goettingen: Hogrefe; 2001.
26. Baddeley A, Emslie H, Nimmo-Smith I. Doors and People: A test of visual and verbal recall and recognition. *Test Company Flempton, Bury St Edmunds.* 1994.
27. Wechsler D. Wechler Adult Intelligence Scale-Revised (San Antonio, TX: The Psychological Corporation). 1981.

28. Schelling D. Block-Tapping-Test. *Frankfurt/Main, Germany: Swets Test Services.* 1997.
29. Bäumler G. Farb-Wort-Interferenztest (FWIT) nach J. R. Stroop. *Göttingen: Hogrefe.* 1985.
30. Zimmermann P, Fimm B. Testbatterie zur Aufmerksamkeitsprüfung [Test of Attentional Performance (TAP)](Version 2.3). *Würselen, Germany: Psytest.* 2012.
31. Morris JC, Heyman A, Mohs RC, et al. The Consortium to Establish a Registry for Alzheimer's Disease (CERAD). Part I. Clinical and neuropsychological assessment of Alzheimer's disease. *Neurology.* 1989;39(9):1159-1165.
32. Frey I, Berg A, Grathwohl D, Keul J. Freiburg Questionnaire of physical activity--development, evaluation and application. *Sozial-und Praventivmedizin.* 1999;44(2):55-64.
33. Buysse DJ, Reynolds CF, Monk TH, Berman SR, Kupfer DJ. The Pittsburgh Sleep Quality Index: a new instrument for psychiatric practice and research. *Psychiatry res.* 1989;28(2):193-213.
34. Eisenberg T, Abdellatif M, Schroeder S, et al. Cardioprotection and lifespan extension by the natural polyamine spermidine. *Nat Med.* 2016;22(12):1428-1438.
35. Willett WC, Sampson L, Stampfer MJ, et al. Reproducibility and validity of a semiquantitative food frequency questionnaire. *American journal of epidemiology.* 1985;122(1):51-65.
36. Schröder H, Fitó M, Estruch R, et al. A short screener is valid for assessing Mediterranean diet adherence among older Spanish men and women. *The Journal of nutrition.* 2011;141(6):1140-1145.
37. Hebestreit K, Yahiaoui-Doktor M, Engel C, et al. Validation of the German version of the Mediterranean Diet Adherence Screener (MEDAS) questionnaire. *BMC cancer.* 2017;17(1):341.
38. Floel A, Witte AV, Lohmann H, et al. Lifestyle and memory in the elderly. *Neuroepidemiology.* 2008;31(1):39-47.
39. Winkler G, Döring A. Validation of a short qualitative food frequency list used in several German large scale surveys. *Zeitschrift für Ernährungswissenschaft.* 1998;37(3):234-241.
40. Stöber J. Besorgnis: Ein Vergleich dreier Inventare zur Erfassung allgemeiner Sorgen. *Zeitschrift für differentielle und diagnostische Psychologie.* 1995.
41. Glöckner-Rist A, Rist F. Deutsche Version des Penn State Worry Questionnaire (PSWQ-d). Paper presented at: Zusammenstellung sozialwissenschaftlicher Items und Skalen2014.
42. Kühner C, Huffziger S, Nolen-Hoeksema S. *RSQ-D: Response Styles Questionnaire; deutsche Version; Manual.* Hogrefe; 2007.
43. Spielberger C, Gorsuch R, Lushene R. Manual for the state-trait anxiety inventory. 1970.
44. Bullinger M, Kirchberger I. *SF-36: Fragebogen zum Gesundheitszustand; Handanweisung.* Hogrefe, Verlag für Psychologie; 1998.
45. Angermeyer MC, Kilian R, Matschinger H. WHOQOL-100 und WHOQOL-BREF. Handbuch für die deutschsprachige Version der WHO Instrumente zur Erfassung von Lebensqualitaet.[WHOQOL-100 and WHOQOL-BREF. Handbook of the German Version of the WHO Instrument to Assess Quality of life]. *Göttingen: Hogrefe.* 2000.
46. Pietrocola F, Malik SA, Mariño G, et al. Coffee induces autophagy in vivo. *Cell Cycle.* 2014;13(12):1987-1994.
47. Mariño G, Pietrocola F, Eisenberg T, et al. Regulation of autophagy by cytosolic acetyl-coenzyme A. *Molecular cell.* 2014;53(5):710-725.
48. Pietrocola F, Castoldi F, Maiuri MC, Kroemer G. Aspirin—another caloric-restriction mimetic. *Autophagy.* 2018;14(7):1162-1163.
49. Troyer AK, Rich JB. Psychometric properties of a new metamemory questionnaire for older adults. *The Journals of Gerontology Series B: Psychological Sciences and Social Sciences.* 2002;57(1):P19-P27.
50. Guralnik JM, Simonsick EM, Ferrucci L, et al. A short physical performance battery assessing lower extremity function: association with self-reported disability and prediction of mortality and nursing home admission. *Journal of gerontology.* 1994;49(2):M85-M94.

Example table for the description of baseline characteristics

Table 2 Baseline characteristics of the study sample

	**Total** n =	**Placebo group** n =	**Spermidine group** n =
**Demographics**			
n (women) [n]			
Age [years]			
Education [years]			
**Screening**			
MMSE [score]			
LMS delayed recall [score]			
TMT A [sec]			
GDS [score]			
**Vascular factors**			
SBP [mmHg]			
DBP [mmHg]			
Heart rate [bpm]			
Weight [kg]			
BMI [kg/m²]			
Waist circumference [cm]			
Diabetes [n]			
Hypertension [n]			
Smoker [n]<br>current<br>former			
**Nutrition parameter**			
Total energy intake [kcal/day]			
MEDAS [score]			
Alcohol intake [g/day]			
**Lifestyle**			
LEQ [score]			
CAI [score]			
**Personality**			
BFI-10, mean [score]			
SVF-78, positive coping [score]			
SVF-78, negative coping [score]			
**Others**			
Oldfield Hand Preference [%]			
MWT [score]			
ECog-39 [score]			
Family history of dementia/AD [n]			

APOE ε4, positive [n]<br>heterozygous<br>homozygous			
Aβ status [n]<br>positive<br>negative			

Data will be given as mean, standard deviation (SD), and range for total as well as the spermidine and placebo group separately. AD: Alzheimer's disease, APOE: apolipoprotein E, Aβ: β-amyloid, BFI-10: Big Five Inventory-10, BMI: body mass index, CAI: Cognitive Activity Interview, cSUVR: composite standardized uptake value ratio, DBP: diastolic blood pressure, ECog-39: Everyday Cognition Scales, LEQ: Lifetime Experience Questionnaire, LMS: Logical Memory Scale, MEDAS: Mediterranean Diet Adherence Screener, MMSE: Mini-Mental State Examination, MWT: Multiple-Choice Vocabulary Intelligence Test (Mehrfachwahl-Wortschatztest), TMT: Trail Making Test, GDS: Geriatric Depression Scale, SBP: systolic blood pressure, SVF-78: Stress Coping Style Questionnaire (Stressverarbeitungsfragebogen).
