# Supplementary material for: Effects of Spermidine Supplementation on Cognition and Biomarkers in Older Adults With Subjective Cognitive Decline: A Randomized Clinical Trial
Source: JAMA Netw Open. 2022 May 26;5(5):e2213875. doi: 10.1001/jamanetworkopen.2022.13875 (PMC9136623; doi:10.1001/jamanetworkopen.2022.13875)
Supplement: Supplement 2. — eMethods 1. Study Design and Participants eMethods 2. Outcomes eMethods 3. Statistical Analyses eAppendix 1. Trial Participants and Compliance eAppendix 2. Subgroup Analyses eAppendix 3. Adverse Events eTable 1. Baseline Assessment of Primary and Secondary Outcomes eTable 2. Adverse Events During Spermidine and Placebo Supplementation eFigure 1. Effect of Spermidine Supplementation on Selected Neuropsychological Parameters—Intention-to-Treat Analysis eFigure 2. Effect of Spermidine Supplementation on Selected Behavioral Parameters—Intention-to-Treat Analysis eFigure 3. Effect of Spermidine Supplementation on Selected Physiological Parameters—Intention-to-Treat Analysis eFigure 4. Effect of Spermidine Supplementation on Neuropsychological Parameters—Per Protocol Analysis eFigure 5. Effect of Spermidine Supplementation on Behavioral Parameters—Per Protocol Analysis eFigure 6. Effect of Spermidine Supplementation on Physiological Parameters—Per Protocol Analysis eFigure 7. Effect of Spermidine Supplementation on Neuropsychological Parameters—Per Protocol Plus Analysis eFigure 8. Effect of Spermidine Supplementation on Behavioral Parameters—Per Protocol Plus Analysis eFigure 9. Effect of Spermidine Supplementation on Physiological Parameters—Per Protocol Plus Analysis eReferences. [file jamanetwopen-e2213875-s002.pdf]

Supplemental Online Content

Schwarz C, Benson GS, Horn N, et al. Effects of spermidine supplementation on cognition and biomarkers in older adults with subjective cognitive decline: a randomized clinical trial. *JAMA Netw Open*. 2022;5(5):e2213875. doi:10.1001/jamanetworkopen.2022.13875

eMethods 1. Study Design and Participants

eMethods 2. Outcomes

eMethods 3. Statistical Analyses

eAppendix 1. Trial Participants and Compliance

eAppendix 2. Subgroup Analyses

eAppendix 3. Adverse Events

eTable 1. Baseline Assessment of Primary and Secondary Outcomes

eTable 2. Adverse Events During Spermidine and Placebo Supplementation

eFigure 1. Effect of Spermidine Supplementation on Selected Neuropsychological Parameters—Intention-to-Treat Analysis

eFigure 2. Effect of Spermidine Supplementation on Selected Behavioral Parameters—Intention-to-Treat Analysis

eFigure 3. Effect of Spermidine Supplementation on Selected Physiological Parameters—Intention-to-Treat Analysis

eFigure 4. Effect of Spermidine Supplementation on Neuropsychological Parameters—Per Protocol Analysis

eFigure 5. Effect of Spermidine Supplementation on Behavioral Parameters—Per Protocol Analysis

eFigure 6. Effect of Spermidine Supplementation on Physiological Parameters—Per Protocol Analysis

eFigure 7. Effect of Spermidine Supplementation on Neuropsychological Parameters—Per Protocol Plus Analysis

eFigure 8. Effect of Spermidine Supplementation on Behavioral Parameters—Per Protocol Plus Analysis

eFigure 9. Effect of Spermidine Supplementation on Physiological Parameters—Per Protocol Plus Analysis

eReferences.

This supplemental material has been provided by the authors to give readers additional information about their work.

© 2022 Schwarz C et al. *JAMA Network Open.*

eMethods 1. Study design and participants

During on-site screening the following measures were applied to determine the absence of objective cognitive impairment and current psychiatric disorders: Mini-Mental State Examination (MMSE) [1] score ≥26, performance within -1.5 standard deviation (SD) of age-adjusted norms in appropriate neuropsychological tests, no deficits on selected items of the Instrumental Activities of Daily Living Scale (IADL) [2], and 15-item Geriatric Depression Scale (GDS) [3] score ≤10 (detailed information on screening procedure are provided in the trial protocol [4]).

Inclusion criteria encompassed a diagnosis of SCD based on established guidelines[5], comprising I) expression of subjective cognitive complaints for at least six months, II) associated concerns (worries), III) affirmation to consult and/or previous consultation of a doctor due to these symptoms, IV) normal cognitive performance, and V) no restrictions on activities of daily living. Potential study participants were excluded if screening revealed a diagnosis of mild cognitive impairment or dementia. Further exclusion criteria comprised severe or untreated medical (e.g., malignant hypertension, untreated diabetes mellitus), neurological or psychiatric disorders, malignancies (current or on medical history), known allergies to wheat germs, gluten or histamine intolerance, pretrial polyamine intake, and alcohol dependency as well as drug abuse.

Overall, 1 g of the administered plant extract contained 1.2 mg spermidine, 0.6 mg spermine, 0.2 mg putrescine, <0.005 mg cadaverine, and 0.166 mg L-ornithine. To ensure double-blinding of the study, both verum and placebo capsules were identical in shape, color, taste, and smell. Participants in both groups were instructed to maintain their dietary habits throughout the intervention period and additionally ingest two capsules with each of their three main meals (six capsules in total per day). To ensure compliance with regular capsule intake, the number of remaining capsules at post-intervention assessment was counted.

© 2022 Schwarz C et al. *JAMA Network Open.*

eMethods 2. Outcomes

Mnemonic discrimination performance was assessed for its proven sensitivity for neural dysfunction in the hippocampus and robustness to detect memory deficits associated with aging and neurodegenerative diseases.[6-8] The mnemonic discrimination index was calculated from the responses of the recognition phase, comparable to previous studies: percentage of lure items endorsed as similar [P("similar"|lure)] plus percentage of lure items classified as new [P("new"|lure)] minus percentage of foil items endorsed as similar [P("similar"|foil)].[8,9] In addition, preliminary evidence of the efficacy of spermidine supplementation on memory performance in humans was shown by the MST in our previous phase IIa study, and thus served as the basis for the power analysis of this study.[9] The MST is a visual computer-based memory task that consists of an encoding and a subsequent recognition phase (available for public download http://faculty.sites.uci.edu/starklab/mnemonic-similarity-task-mst/).

Secondary outcomes encompassed additional neuropsychological, behavioral, and physiological parameters. Neuropsychological parameters were evaluated in different cognitive domains, including verbal and visual-spatial memory (e.g., learning ability, delayed recall, and recognition as parts of the German version of the Rey Auditory Verbal Learning Test [AVLT, VLMT]), attention (e.g., Test for Attentional Performance battery [TAP]), and executive functions (e.g., Trail Making Test B). Behavioral parameters were assessed by questionnaires on lifestyle, psycho-affective measures, and perceived quality of life. Physiological parameters were assessed in a standardized medical examination with fasting blood sampling, encompassing among others analysis of hematological safety, inflammation, and vascular injury parameters. In addition, genotyping of blood-derived deoxyribonucleic acid for apolipoprotein E (APOE) ε4 status and the assessment of cardiovascular risk factors (e.g., blood pressure, weight) were part of the medical examination.

Note that the calculation for the Stroop test interference was based on the following formula: Stroop named color-word - ((Stroop reading + Stroop colors naming) / 2).[10] Moreover, dietary intake of polyamines per day, assessed through the self-reported 89-item food frequency questionnaire, was analyzed similar to previous studies.[11-13]

Plasma-based inflammatory and vascular injury biomarkers (e.g., soluble intercellular adhesion molecule-1 [sICAM-1] and interleukins) were analyzed by the Institute of Molecular Biosciences, University of Graz, Austria using the commercially available V-PLEX Proinflammatory Panel 1 (human) and Vascular Injury Panel 2 (human) Kits (Meso Scale Diagnostics, USA). Missing data in the proinflammatory panel that were below the fit curve range were replaced by the half median of the lower limit of detection for each analyte as provided by the manufacturer (case for interleukin-6 and interleukin-10). In addition, whole blood polyamine levels were measured in the same laboratory with support from Joanneum Research HEALTH, Graz, Austria. Polyamine extraction and quantification using high performance liquid chromatography coupled to mass spectrometry (LC-MS/MS) was performed essentially as described previously.[14,15] Polyamine derivatives were extracted by SPE (Strata-X, Polymeric Reversed Phase, 96 well plate). SPE was conditioned with 500µl acetonitrile, equilibrated with 500µl dest. water containing 0.2% acetic acid. Trichloracid extracts were loaded onto the SPE and after two washing steps with 500µl 0.2% acetic acid samples were eluted with 250µl 80% acetonitrile containing 0.2% acetic acid. Eluted SPE extracts were subjected to LC-MS/MS (mobile phase: isocratic 80% acetonitrile containing 0.2% acetic acid; flow rate 250µl/min; HPLC column: Kinetex 2,6µm C18 100A 50mm x 2,1mm).

© 2022 Schwarz C et al. *JAMA Network Open.*

eMethods 3. Statistical analyses

Overall, 100 participants (50 in each group; including 10% dropout rate) were needed to provide 80% power to detect a statistically significant intervention effect for the primary endpoint (mnemonic discrimination performance at post-intervention assessment compared with baseline; two-sided significance level α=0.05) using an unpaired-sample t-test if the true effect is 0.6 (Cohen's d). Baseline characteristics of all participants were reported descriptively using mean, standard deviation, percentage, and range, if applicable, as well as for each intervention group separately. For safety analysis, AEs and SAEs were grouped into system organ classes using the Medical Dictionary for Regulatory Activities (MedDRA system, version 23.1, http://www.meddra.org/) and were reported descriptively including the following parameters: incidence of (S)AEs, intensity, and relationship to intervention.

Pre-specified subgroup analyses for the primary outcome and neuropsychological secondary outcomes were performed according to age, sex, apolipoprotein E phenotype, severity of subjective cognitive complaints using the 39-item Everyday Cognition Scales, defined categories of dietary spermidine intake using the Food Frequency Questionnaire, and mnemonic discrimination index at baseline. In addition, we performed further exploratory subgroup analyses according to cognitive performance at baseline using a composite score of global cognitive measure similar to the Preclinical Alzheimer's Cognitive composite (PACC) and change in blood spermidine levels from baseline to post-intervention visit. For each subgroup analysis, we conducted an analysis of covariance model (adjusted for age, sex, and particular baseline measure) and tested the interaction between intervention group and subgroup to test for differential treatment effects on neuropsychological outcome measures. Additionally, when a differential treatment effect was observed (interaction term $P<0.05$), treatment effects by subgroup with 95% CI were calculated as marginal means of the models. All subgroup analyses were conducted in the full analysis, per protocol, and per protocol *plus* set. Furthermore, we exploratory investigated the association between blood spermidine levels and neuropsychological outcome measures at post-intervention visit, regardless of the arm of intervention (adjusted for age and sex).

For all analyses appropriate descriptive statistics (mean, median, standard deviation [SD], interquartile range, 95% CI) depending on the scale and distribution of the outcome variable were presented. All analyses were performed using SPSS 24.0 statistical package (PASW, SPSS; IBM, Armonk, NY, USA) and R statistical software version 4.0.3.

© 2022 Schwarz C et al. *JAMA Network Open.*

eAppendix 1. Trial participants and compliance

The total sample of participants (n=100) exhibited the following SCD features at baseline: onset of SCD between 1 and 10 years (mean: 3.5, SD: 2.3) in the age range of 53 to 78 (mean: 65.7, SD: 5.7), and a positive family history of dementia in 29 participants (29%).

For assessing compliance to intervention, participants were also asked at post-intervention assessment for their subjective feeling of capsule compliance (6.7% never forgot intake, 42.7% forgot max. 5 intakes in total, 43.8% forgot max. 5 intakes per month, 6.7% forgot max. 5 intakes per week), yielding similar results as counting the number of remaining capsules at post-intervention assessment. Furthermore, efficacy of blinding from the participants' perspective was determined by asking each participant at post-intervention visit to provide guesses of the assigned intervention group, showed no association of guesses with actual randomization (47 out of 89 [52.8%] participants correctly guessed the group allocation, 95% CI of correct guessing: 41.9% - 63.5%, P=.67 in binomial test).

© 2022 Schwarz C et al. *JAMA Network Open.*

eAppendix 2. Subgroup analyses

In the per protocol set, subgroup analyses for mnemonic discrimination performance suggested a small negative intervention effect in participants with higher baseline dietary spermidine intake than in participants with lower baseline dietary spermidine intake: -0.11 (95% CI: -0.23 to 0, *P*=.05; favoring placebo) *v* 0.04 (95% CI: -0.07 to 0.15, *P*=.80); *P*=.01 for interaction. In parallel, a greater intervention effect was observed for the German version of the Rey Auditory Verbal Learning Test (VLMT) learning ability in older participants compared to younger participants: 4.8 (95% CI: 0.1 to 9.6, *P*=.04; favoring spermidine) *v* -0.9 (95% CI: -4.9 to 3.1, *P*=.93); *P*=.02 for interaction. In the per protocol *plus* set, subgroup analyses suggested a greater effect of intervention on VLMT learning ability in older participants compared to younger participants: 5.7 (95% CI: 0.2 to 11.2, *P*=.04; favoring spermidine) *v* -0.5 (95% CI: -5.0 to 4.1, *P*=0.99); *P*=.03 for interaction. A similar picture was observed for subgroup analyses on sex, showing a greater intervention effect on VLMT learning ability in male participants compared to female participants: 5.7 (95% CI: 0.6 to 10.8, *P*=.02; favoring spermidine) *v* -1.5 (95% CI: -6.3 to 3.3, *P*=.84); *P*=.008 for interaction. Moreover, a small negative effect of intervention in the Trail Making Test B (TMT B) was observed for older participants compared to younger participants: 32.44 sec (95% CI: 6.8 to 58.1, *P*=.007; favoring placebo) *v* -0.04 sec (95% CI: -21.5 to 21.4, *P*=1.00); *P*=.01 for interaction. In addition, a greater effect of spermidine supplementation was observed for VLMT recognition performance in participants with more severe cognitive complaints compared to participants with less severe cognitive complaints: 2.5 (95% CI: 0.1 to 4.9, *P*=.04; favoring spermidine) *v* -0.4 (95% CI: -2.9 to 2.2, *P*=.98); *P*=.04 for interaction. All other pre-specified subgroup analyses showed no differential effects of intervention.

In our additional exploratory subgroup analyses, a greater intervention effect was observed in the per protocol *plus* set for VLMT learning ability in participants with lower PACC scores compared to participants with higher PACC scores: 5.4 (95% CI: 0.4 to 10.5, *P*=.03; favoring spermidine) *v* -1.6 (95% CI: -6.6 to 3.4, *P*=.83); *P*=.01 for interaction. All other non-pre-specified subgroup analyses showed no differential effects of intervention, and no main effects of blood spermidine levels were observed on neuropsychological outcome measures at post-intervention visit.

© 2022 Schwarz C et al. *JAMA Network Open.*

eAppendix 3. Adverse events

Moreover, 22 AEs were documented in the system organ class of gastrointestinal disorders, with descriptively more observations in the placebo group (n=13) compared to the spermidine group (n=9) and an incidence rate ratio of 0.73 (95% CI: 0.30 to 1.68). One participant reported three AEs and five participants reported two AEs in this system organ class. In total, three participants (spermidine group: n=2, placebo group: n=1) discontinued capsule intake and study participation because of AEs in this system organ class, including nausea, meteorism, and reflux. The two dropouts in the spermidine group could be explained by possible unknown allergies or intolerances against gluten or wheat germs.

© 2022 Schwarz C et al. *JAMA Network Open.*

eTable 1. Baseline assessment of primary and secondary outcomes

	Mean (SD)		
**Parameter**	**Total** n=100	**Spermidine** n=51	**Placebo** n=49
**PRIMARY OUTCOME**			
MST, Mnemonic discrimination (score)	0.4 (0.2)	0.4 (0.2)	0.4 (0.2)
**SECONDARY OUTCOMES**			
**Neuropsychological**			
Memory			
MST, Recognition memory (score), median (IQR)	0.8 (0.7-0.9)	0.8 (0.7-0.9)	0.8 (0.7-0.9)
VLMT, Learning ability (score)	51.3 (10.0)	50.3 (9.9)	52.3 (10.1)
VLMT, Delayed recall, forgotten words (score)	2.2 (2.1)	2.1 (2.1)	2.2 (2.2)
VLMT, Recognition (score), median (IQR)	13.0 (9.0-14.0)	13.0 (9.0-14.0)	14.0 (10.5-15.0)
Doors and People Test, Visual recognition (score)	17.7 (2.9)	17.5 (3.1)	17.8 (2.7)
Executive function			
TMT B (sec)	92.3 (33.1)	89.0 (33.0)	95.8 (33.1)
Digit Span forward (score)	7.5 (2.0)	7.4 (2.1)	7.6 (2.0)
Digit Span backward (score)	6.7 (1.7)	6.7 (1.8)	6.7 (1.6)
Block Tapping forward (score)	7.9 (1.6)	8.0 (1.5)	7.7 (1.7)
Block Tapping backward (score)	7.0 (1.6)	6.9 (1.7)	7.1 (1.5)
Digit Symbol (score)	46.8 (9.8)	46.3 (9.9)	47.3 (9.8)
Stroop, Interference (sec)	48.9 (17.1)	48.4 (16.8)	49.5 (17.6)
Language			
Phonemic Fluency (score)	12.2 (4.2)	11.8 (4.1)	12.6 (4.4)
Semantic Fluency (score)	22.7 (5.5)	22.0 (5.1)	23.4 (6.0)
Boston Naming Test (score), median (IQR)	15 (14-15)	15 (14-15)	15 (14-15)
Attention			
TAP Alertness, median RT uncued trials (sec), median (IQR)	296.8 (265.9-357.8)	299.0 (263.0-349.0)	292.0 (269.5-368.8)
TAP Alertness, median RT cued trials (sec), median (IQR)	293.3 (262.8-339.1)	297.0 (262.0-344.0)	287.0 (263.5-338.0)
TAP Alertness, Phasic alertness	0.001 (0.153)	-0.023 (0.173)	0.025 (0.126)
TAP Divided Attention, Auditory median RT (sec)	672.4 (122.8)	664.6 (116.3)	680.5 (130.0)
TAP Divided Attention, Visual median RT (sec)	873.5 (105.7)	873.5 (107.4)	873.6 (105.0)
TAP Divided Attention, Omissions (score), median (IQR)	2 (1-3)	2 (1-3)	1 (0-3)
**Behavioral**			
Psycho-affective/worry			
MMQ (score)	7.8 (4.3)	8.1 (4.1)	7.5 (4.6)
RSQ−D, Self-focused rumination (score)	11.7 (3.4)	11.1 (3.3)	12.4 (3.3)
RSQ−D, Symptom-focused rumination (score)	13.6 (3.7)	13.4 (4.2)	13.8 (3.2)
RSQ−D, Distraction (score)	19.3 (4.8)	18.0 (5.0)	20.6 (4.3)
PSWQ (score)	41.6 (10.7)	42.5 (11.5)	40.7 (9.8)
STAI-G X1 (score)	35.4 (8.8)	35.5 (10.3)	35.4 (7.0)
Lifestyle			
CAI present (score)	3.4 (0.6)	3.4 (0.7)	3.4 (0.6)
PSQI (score)	5.8 (2.9)	6.2 (3.4)	5.4 (2.3)
FKA, Lifestyle (score)	16.6 (2.5)	16.7 (2.6)	16.6 (2.3)
FFQ, Spermidine	1.7 (0.2)	1.7 (0.2)	1.7 (0.2)
FFQ, Spermine	1.3 (0.2)	1.3 (0.1)	1.3 (0.2)

© 2022 Schwarz C et al. *JAMA Network Open.*

	Mean (SD)		
**Parameter**	**Total n=100**	**Spermidine n=51**	**Placebo n=49**
FFQ, Putrescine	2.0 (0.2)	2.1 (0.2)	2.0 (0.2)
SF-12, Physical health (score)	49.4 (7.3)	48.7 (8.0)	50.2 (6.6)
SF-12, Mental health (score)	52.1 (7.9)	52.3 (8.6)	51.8 (7.1)
WHOQOL−BREF, Physical (score)	76.5 (14.1)	76.1 (13.8)	76.9 (14.6)
WHOQOL−BREF, Psychological (score)	71.5 (12.0)	71.4 (11.8)	71.6 (12.3)
WHOQOL−BREF, Social (score)	69.7 (16.1)	69.4 (15.2)	69.9 (17.1)
WHOQOL−BREF, Environmental (score)	82.6 (8.6)	82.8 (8.4)	82.5 (8.9)
WHOQOL-BREF, Overall (score)	70.8 (14.6)	69.1 (12.6)	72.4 (16.3)
**Physiological**			
Leukocytes (/nl)	5.9 (1.5)	6.1 (1.6)	5.6 (1.4)
Erythrocytes (/pg)	4.7 (0.4)	4.7 (0.4)	4.7 (0.4)
Quick (%), median (IQR)	100.0 (95.0-104.0)	100.0 (93.0-104.0)	100.0 (96.3-104.8)
INR, median (IQR)	1.00 (0.97-1.03)	1.00 (0.97-1.05)	1.00 (0.97-1.02)
Fibrinogen (g/l)	3.2 (0.6)	3.2 (0.5)	3.1 (0.6)
Cholesterol (mg/dl)	204.0 (43.9)	195.7 (43.7)	212.7 (42.8)
HDL cholesterol (mg/dl)	67.4 (20.7)	65.6 (21.4)	69.3 (20.0)
LDL cholesterol (mg/dl)	131.7 (39.7)	124.9 (38.6)	138.8 (39.9)
Triglycerides (mg/dl)	102.9 (51.3)	101.2 (52.8)	104.7 (50.2)
Glucose (mg/dl)	94.1 (16.3)	94.4 (18.1)	93.8 (14.2)
HbA1c (%)	5.5 (0.4)	5.5 (0.5)	5.5 (0.3)
Insulin (mU/l)	8.2 (4.7)	8.3 (5.3)	8.1 (4.0)
Homocysteine (µmol/l)	15.8 (4.1)	15.8 (4.2)	15.7 (4.2)
Liver			
AST (U/l), median (IQR)	27.0 (23.0-31.8)	29.0 (24.0-33.0)	26.0 (23.0-29.0)
ALT (U/l), median (IQR)	22.5 (16.3-27.8)	23.0 (17.0-30.0)	21.0 (15.0-25.0)
Kidney			
Creatinine (mg/dl)	0.8 (0.2)	0.8 (0.1)	0.9 (0.2)
eGFR, median (IQR)	85.0 (76.3-90.0)	86.0 (81.0-90.0)	82.0 (75.0-90.0)
Inflammation/vascular injury			
CRP (mg/l), median (IQR)	1.7 (0.7-3.1)	2.0 (0.7-3.4)	1.3 (0.7-2.7)
SAA (mg/l), median (IQR)	2.5 (1.3-4.3)	2.8 (1.3-3.9)	2.1 (1.3-4.8)
sICAM-1 (ng/ml)	356.5 (99.2)	354.5 (101.1)	358.6 (98.2)
sVCAM-1 (ng/ml)	469.9 (120.7)	457.2 (123.2)	483.0 (118.0)
IFN−gamma (pg/ml), median (IQR)	2.5 (1.5-3.3)	2.6 (1.5-3.2)	2.3 (1.4-4.3)
IL−10 (pg/ml), median (IQR)	0.11 (0.02-0.15)	0.10 (0.02-0.13)	0.11 (0.09-0.16)
IL−6 (pg/ml), median (IQR)	0.21 (0.03-0.35)	0.20 (0.03-0.36)	0.21 (0.03-0.34)
IL−8 (pg/ml), median (IQR)	1.51 (1.22-1.95)	1.48 (1.16-2.01)	1.53 (1.30-1.85)
TNF−alpha (pg/ml)	1.3 (0.4)	1.3 (0.3)	1.3 (0.4)
Polyamines			
Spermidine (µM)	8.1 (1.9)	8.5 (2.0)	7.8 (1.8)
Spermine (µM), median (IQR)	4.6 (3.7-6.3)	4.7 (3.7-6.8)	4.4 (3.4-5.7)
L-Ornithine (µM)	96.8 (16.2)	97.2 (16.6)	96.4 (16.0)

Missing data of one participant of the spermidine group for the MST (mnemonic discrimination performance and recognition memory) as well as for blood levels of spermidine, spermine, and L-ornithine. Missing data of one participant of the placebo group for Quick and INR. For homocysteine level data of five participants (spermidine group: n=3, placebo group: n=2) were missing. Abbreviations: ALT, alanine aminotransferase; AST, aspartate aminotransferase; CAI, Cognitive Activity Interview; CRP, c-reactive protein; eGFR, estimated glomerular filtration rate; FFQ, Food Frequency Questionnaire; FKA, Freiburger Fragebogen zur körperlichen Aktivität; HbA1c, hemoglobin A1c; HDL, high-density lipoprotein; IFN-gamma, interferon gamma; IL, interleukin; INR, international normalized ratio of blood clotting; IQR, interquartile range; LDL, low-density lipoprotein; MMQ, Meta Memory Questionnaire; MST, Mnemonic Similarity Task; PSQI, Pittsburgh Sleep Quality Index; PSWQ, Penn State Worry Questionnaire; RSQ-D, Response Styles Questionnaire – German Version; RT, reaction time; SAA, Serum amyloid A; SF-12, Short Form Health Survey; sICAM-1, soluble intercellular adhesion molecule-1; STAI-X1, State-Trait Anxiety Inventory – X1; sVCAM-1, soluble vascular cell adhesion molecule-1; TAP, Test for Attentional Performance battery; TMT, Trail Making Test; TNF-alpha, tumor necrosis factor alpha; VLMT, Verbaler Lern- und Merkfähigkeitstest, German version of the Rey Auditory Verbal Learning Test (AVLT); WHOQOL-BREF, World Health Organization Quality of Life.

© 2022 Schwarz C et al. *JAMA Network Open.*

eTable 2. Adverse events during spermidine and placebo supplementation

	Participants, No., IR per 100 person years (95% CI)			IRR (95% CI)	*P-value*
	**Total n=100**	**Spermidine n=51**	**Placebo n=49**		
**Total number of AEs**	129<br>134.2<br>(112.4-158.7)	58<br>123.6<br>(94.4-158.1)	71<br>144.4<br>(113.4-180.6)	--<br>0.86<br>(0.60-1.21)	0.38
**Cardiac disorders**	2<br>2.1<br>(0.3-6.4)	2<br>4.3<br>(0.7-13.1)	0<br>--<br>--	--<br>--<br>--	1.00
**Endocrine disorders**	1<br>1.0<br>(0.1-4.6)	1<br>2.1<br>(0.1-9.4)	0<br>--<br>--	--<br>--	1.00
**Eye disorders**	4<br>4.2<br>(1.3-9.7)	1<br>2.1<br>(0.1-9.4)	3<br>6.1<br>(1.5-15.8)	--<br>0.35<br>(0.02-2.73)	0.36
**Gastrointestinal disorders**	22<br>22.9<br>(14.6-33.8)	9<br>19.2<br>(9.2-34.6)	13<br>26.4<br>(14.5-43.5)	--<br>0.73<br>(0.30-1.68)	0.46
**Hepatobiliary disorders**	2<br>2.1<br>(0.3-6.4)	0<br>--<br>--	2<br>4.1<br>(0.7-12.6)	--<br>--<br>--	1.00
**Injury, poisoning and procedural complications**	8<br>8.3<br>(3.8-15.5)	2<br>4.3<br>(0.7-13.1)	6<br>12.2<br>(4.8-24.7)	--<br>0.35<br>(0.05-1.52)	0.20
**Musculoskeletal and connective tissue disorders**	15<br>15.6<br>(9.0-24.9)	11<br>23.4<br>(12.2-40.1)	4<br>8.1<br>(2.5-18.9)	--<br>2.88<br>(0.99-10.40)	0.07
**Neoplasms benign, malignant and unspecified**	2<br>2.1<br>(0.3-6.4)	1<br>2.1<br>(0.1-9.4)	1<br>2.0<br>(0.1-8.9)	--<br>1.05<br>(0.04-26.49)	0.97
**Nervous system disorders**	6<br>6.2<br>(2.5-12.6)	3<br>6.4<br>(1.6-16.6)	3<br>6.1<br>(1.5-15.8)	--<br>1.05<br>(0.19-5.66)	0.96
**Psychiatric disorders**	3<br>3.1<br>(0.8-8.1)	1<br>2.1<br>(0.1-9.4)	2<br>4.1<br>(0.7-12.6)	--<br>0.52<br>(0.02-5.47)	0.60
**Renal and urinary disorders**	5<br>5.2<br>(1.9-11.2)	3<br>6.4<br>(1.6-16.6)	2<br>4.1<br>(0.7-12.6)	1.57<br>(0.26-11.93)	0.62
**Reproductive system and breast disorders**	2<br>2.1<br>(0.3-6.4)	0<br>--<br>--	2<br>4.1<br>(0.7-12.6)	--<br>--<br>--	1.00
**Respiratory, thoracic and mediastinal disorders**	20<br>20.8<br>(13.0-31.3)	7<br>14.9<br>(6.4-28.8)	13<br>26.4<br>(14.5-43.5)	0.56<br>(0.21-1.38)	0.22
**Skin and subcutaneous tissue disorders**	9<br>9.4<br>(4.5-16.9)	5<br>10.7<br>(3.8-22.9)	4<br>8.1<br>(2.5-18.9)	1.31<br>(0.35-5.29)	0.69
**Surgical and medical procedures**	21<br>21.8<br>(13.8-32.6)	9<br>19.2<br>(9.2-34.6)	12<br>24.4<br>(13.1-40.9)	--<br>0.79<br>(0.32-1.86)	0.58
**Vascular disorders**	7<br>7.3<br>(3.1-14.1)	3<br>6.4<br>(1.6-16.6)	4<br>8.1<br>(2.5-18.9)	--<br>0.79<br>(0.15-3.56)	0.75

Abbreviations: AE, adverse event; CI, confidence interval; IQR, interquartile range; IR, incidence rate; IRR, incidence rate ratio.

© 2022 Schwarz C et al. *JAMA Network Open.*

eFigure 1. Effect of spermidine supplementation on selected neuropsychological parameters – intention-to-treat analysis

Parameter	Participants	Mean change placebo (95% CI)	Mean change spermidine (95% CI)	Group differences (95% CI)	P-value
Memory					
MST, Recognition memory (score)	100	0.01 (-0.05 – 0.07)	-0.02 (-0.09 – 0.04)	-0.03 (-0.12 – 0.05)	0.42
VLMT, Delayed recall, forgotten words (score)	100	-0.18 (-1.03 – 0.67)	0.26 (-0.67 – 1.20)	-0.44 (-1.71 – 0.82)	0.49
Doors Test, Visual recognition (score)	100	0.72 (-0.18 – 1.62)	1.05 (-0.08 – 2.18)	0.33 (-1.11 – 1.76)	0.65
Executive function					
Digit Span forward (score)	100	0.28 (-0.24 – 0.80)	0.01 (-0.67 – 0.69)	-0.27 (-1.17 – 0.63)	0.55
Block Tapping Test forward (score)	100	0.06 (-0.39 – 0.52)	-0.10 (-0.61 – 0.41)	-0.16 (-0.87 – 0.54)	0.64
Digit Symbol (score)	100	0.86 (-1.78 – 3.49)	0.04 (-3.44 – 3.53)	-0.81 (-5.16 – 3.53)	0.71
Stroop, Interference (sec)	100	-1.60 (-8.49 – 5.30)	1.62 (-6.35 – 9.60)	-3.22 (-13.74 – 7.30)	0.54
Language					
Phonemic Fluency (score)	100	-0.01 (-1.19 – 1.18)	0.28 (-1.07 – 1.64)	0.29 (-1.49 – 2.07)	0.74
Semantic Fluency (score)	100	0.42 (-1.45 – 2.29)	0.83 (-1.40 – 3.07)	0.42 (-2.48 – 3.31)	0.77
Boston Naming Test (score)	100	-0.07 (-0.40 – 0.25)	-0.26 (-0.64 – 0.11)	-0.19 (-0.70 – 0.31)	0.45
Attention					
TAP Alertness, median RT uncued trials (sec)	100	3.37 (-18.53 – 25.28)	13.59 (-10.39 – 37.58)	-10.22 (-42.89 – 22.45)	0.53
TAP Alertness, median RT cued trials (sec)	100	5.11 (-15.33 – 25.56)	9.20 (-13.28 – 31.69)	-4.09 (-33.69 – 25.51)	0.78
TAP Alertness, phasic alertness	100	-0.01 (-0.05 – 0.04)	0.00 (-0.05 – 0.05)	0.01 (-0.06 – 0.07)	0.82
TAP Divided attention, auditory median RT (sec)	100	9.48 (-56.04 – 74.99)	7.25 (-63.29 – 77.79)	2.23 (-90.38 – 94.84)	0.96
TAP Divided attention, visual median RT (sec)	100	-10.14 (-43.54 – 23.26)	-19.09 (-55.94 – 17.75)	8.95 (-43.85 – 61.76)	0.73
TAP Divided attention, omissions (score)	100	0.47 (-0.51 – 1.45)	0.47 (-0.73 – 1.66)	0.00 (-1.59 – 1.59)	1.00

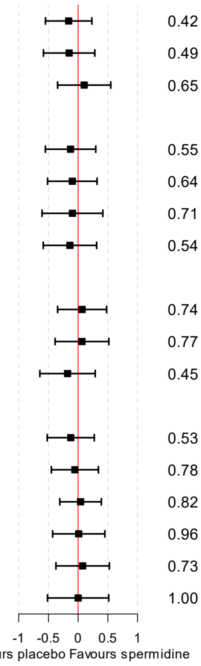

Missing data was imputed using multivariate imputation by chained equations (mice) based on 30 imputed datasets and predictive mean matching. Mean changes of each parameter from baseline to post-intervention assessment with 95% CI are presented for both intervention groups separately. Group differences and p-values result from analysis of covariance models for change in outcome from baseline to post-intervention visit, with intervention group as factor and adjusted for age, sex, and the particular baseline measure. Forest plots indicate mean group differences with 95% CI, standardized by being converted into z-scores. Forest plots and group differences were transformed, if necessary, to yield same direction of effect (positive values: favor spermidine, negative values: favor placebo). Abbreviations: MST, Mnemonic Similarity Task; RT, reaction time; TAP, Test for Attentional Performance battery; VLMT, Verbaler Lern- und Merkfähigkeitstest, German version of the Rey Auditory Verbal Learning Test (AVLT).

eFigure 2. Effect of spermidine supplementation on selected behavioral parameters – intention-to-treat analysis

Parameter	Participants	Mean change placebo (95% CI)	Mean change spermidine (95% CI)	Group differences (95% CI)	P-value
Psycho-affective/worry					
MMQ (score)	100	1.63 (0.14 – 3.13)	1.74 (0.12 – 3.36)	0.10 (-2.14 – 2.35)	0.93
RSQ-D, Self-focused rumination (score)	100	-0.27 (-1.26 – 0.72)	0.21 (-1.00 – 1.42)	-0.48 (-1.99 – 1.04)	0.53
RSQ-D, Symptom-focused rumination (score)	100	0.06 (-1.12 – 1.24)	-0.06 (-1.36 – 1.24)	0.12 (-1.63 – 1.88)	0.89
RSQ-D, Distraction (score)	100	0.00 (-1.41 – 1.42)	-0.60 (-2.17 – 0.97)	-0.60 (-2.76 – 1.56)	0.58
STAI-X1 (score)	100	-0.15 (-2.76 – 2.47)	-0.64 (-3.58 – 2.30)	0.50 (-3.37 – 4.36)	0.80
Lifestyle					
CAI present (score)	100	0.02 (-0.16 – 0.20)	0.11 (-0.11 – 0.33)	0.09 (-0.18 – 0.37)	0.50
FKA, Lifestyle (score)	100	-0.03 (-0.95 – 0.90)	-0.36 (-1.37 – 0.65)	-0.33 (-1.68 – 1.01)	0.62
MEDAS (score)	100	0.02 (-0.68 – 0.73)	0.40 (-0.48 – 1.27)	0.37 (-0.80 – 1.55)	0.53
FFQ, Spermidine	100	0.01 (-0.07 – 0.09)	0.02 (-0.07 – 0.11)	0.01 (-0.12 – 0.13)	0.91
FFQ, Spermine	100	0.04 (-0.03 – 0.11)	-0.00 (-0.08 – 0.07)	-0.04 (-0.14 – 0.06)	0.39
FFQ, Putrescine	100	-0.03 (-0.07 – 0.02)	-0.02 (-0.07 – 0.03)	0.00 (-0.07 – 0.07)	0.96
WHOQOL-BREF, Physical (score)	100	-0.95 (-5.83 – 3.93)	-2.25 (-7.64 – 3.14)	-1.30 (-8.79 – 6.19)	0.73
WHOQOL-BREF, Psychological (score)	100	-0.72 (-4.69 – 3.26)	-1.43 (-5.56 – 2.70)	-0.71 (-6.63 – 5.20)	0.81
WHOQOL-BREF, Social (score)	100	-3.05 (-7.64 – 1.54)	0.20 (-5.24 – 5.64)	3.25 (-3.76 – 10.25)	0.36
WHOQOL-BREF, Environmental (score)	100	0.99 (-1.79 – 3.78)	-0.90 (-4.17 – 2.36)	-1.90 (-6.33 – 2.54)	0.39

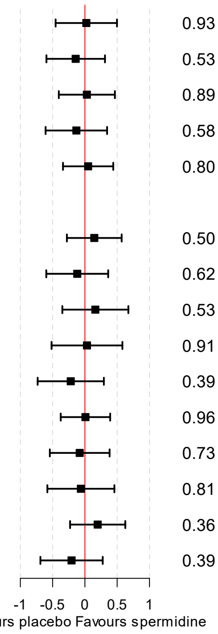

Missing data was imputed using multivariate imputation by chained equations (mice) based on 30 imputed datasets and predictive mean matching. Mean changes of each parameter from baseline to post-intervention assessment with 95% CI are presented for both intervention groups separately. Group differences and P-values result from analysis of covariance models for change in outcome from baseline to post-intervention visit, with intervention group as factor and adjusted for age, sex, and the particular baseline measure. Forest plots indicate mean group differences with 95% CI, standardized by being converted into z-scores. Forest plots and group differences were transformed, if necessary, to yield same direction of effect (positive values: favor spermidine, negative values: favor placebo). Abbreviations: CAI, Cognitive Activity Interview; FFQ, Food Frequency Questionnaire; FKA, Freiburger Fragebogen zur körperlichen Aktivität; MEDAS, Mediterranean Diet Adherence Screener; MMQ, Meta Memory Questionnaire; RSQ-D, Response Styles Questionnaire – German Version; STAI-X1, State-Trait Anxiety Inventory – X1; WHOQOL-BREF, World Health Organization Quality of Life.

© 2022 Schwarz C et al. *JAMA Network Open.*

eFigure 3. Effect of spermidine supplementation on selected physiological parameters – intention-to-treat analysis

Parameter	Participants	Mean change placebo (95% CI)	Mean change spermidine (95% CI)	Group differences (95% CI)	P-value
Quick (%)	100	-3.81 (-9.18 – 1.56)	-7.80 (-13.48 – -2.13)	-3.99 (-11.33 – 3.35)	0.28
Fibrinogen (g/l)	100	-0.02 (-0.27 – 0.23)	0.03 (-0.23 – 0.30)	-0.05 (-0.42 – 0.31)	0.77
Cholesterol (mg/dl)	100	1.68 (-10.78 – 14.13)	-5.80 (-19.99 – 8.40)	7.47 (-12.13 – 27.08)	0.45
Glucose (mg/dl)	100	-0.42 (-5.39 – 4.55)	-0.95 (-6.80 – 4.90)	0.53 (-6.87 – 7.93)	0.89
HbA1c (%)	100	0.16 (0.02 – 0.30)	0.11 (-0.05 – 0.27)	0.05 (-0.16 – 0.26)	0.64
Insulin (mU/l)	100	1.10 (-5.00 – 7.20)	4.52 (-2.28 – 11.32)	3.42 (-5.57 – 12.41)	0.45
Homocysteine (µmol/l)	100	0.23 (-1.47 – 1.94)	0.48 (-1.46 – 2.41)	-0.24 (-2.81 – 2.32)	0.85
Inflammation/vascular injury					
SAA (mg/l)	100	-3.34 (-5.26 – -1.43)	-3.31 (-5.38 – -1.25)	-0.03 (-1.90 – 1.83)	0.97
IL-10 (pg/ml)	100	0.05 (-0.05 – 0.15)	0.08 (-0.03 – 0.19)	0.03 (-0.12 – 0.18)	0.66
IL-6 (pg/ml)	100	0.09 (-0.03 – 0.21)	0.16 (0.03 – 0.28)	-0.06 (-0.24 – 0.11)	0.46
IL-8 (pg/ml)	100	1.24 (-1.33 – 3.82)	1.11 (-1.48 – 3.69)	0.14 (-3.67 – 3.94)	0.94
Cardiovascular risk					
Weight (kg)	100	-0.93 (-4.09 – 2.22)	-0.14 (-4.45 – 4.18)	-0.80 (-6.22 – 4.63)	0.77

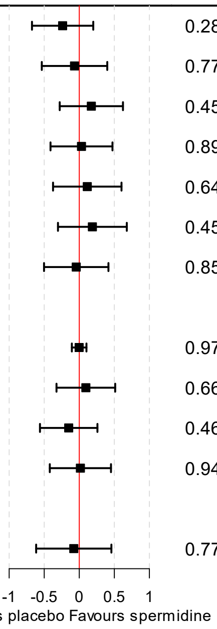

Missing data was imputed using multivariate imputation by chained equations (mice) based on 30 imputed datasets and predictive mean matching. Mean changes of each parameter from baseline to post-intervention assessment with 95% CI are presented for both intervention groups separately. Group differences and P-values result from analysis of covariance models for change in outcome from baseline to post-intervention visit, with intervention group as factor and adjusted for age, sex, and the particular baseline measure. Forest plots indicate mean group differences with 95% CI, standardized by being converted into z-scores. Forest plots and group differences were transformed, if necessary, to yield same direction of effect (positive values: favor spermidine, negative values: favor placebo). Abbreviations: HbA1c, hemoglobin A1c; IL, interleukin; SAA, Serum amyloid A.

© 2022 Schwarz C et al. *JAMA Network Open.*

eFigure 4. Effect of spermidine supplementation on neuropsychological parameters – per protocol analysis

Parameter	Participants	Mean change placebo (95% CI)	Mean change spermidine (95% CI)	Group differences (95% CI)	P-value
Memory					
MST, Mnemonic discrimination (score)	89	0.01 (-0.03 – 0.05)	-0.03 (-0.08 – 0.02)	-0.04 (-0.10 – 0.03)	0.25
MST, Recognition memory (score)	89	0.02 (-0.02 – 0.07)	0.00 (-0.05 – 0.05)	-0.02 (-0.09 – 0.04)	0.52
VLMT, Learning ability (score)	89	2.82 (1.16 – 4.47)	4.13 (2.38 – 5.88)	1.31 (-1.10 – 3.73)	0.28
VLMT, Delayed recall, forgotten words (score)	89	-0.24 (-0.87 – 0.39)	0.05 (-0.62 – 0.71)	-0.29 (-1.21 – 0.63)	0.53
VLMT, Recognition (score)	89	-0.04 (-0.92 – 0.84)	1.06 (0.13 – 1.98)	1.09 (-0.19 – 2.37)	0.09
Doors and People Test, Visual recognition (score)	89	0.79 (0.12 – 1.46)	1.20 (0.49 – 1.90)	0.41 (-0.57 – 1.38)	0.41
Executive function					
TMT B (sec)	89	-4.15 (-12.27 – 3.97)	6.11 (-2.48 – 14.70)	-10.26 (-22.18 – 1.66)	0.09
Digit Span forward (score)	89	0.30 (-0.10 – 0.71)	0.02 (-0.41 – 0.44)	-0.28 (-0.87 – 0.30)	0.34
Digit Span backward (score)	89	-0.33 (-0.77 – 0.11)	-0.46 (-0.93 – 0.01)	-0.13 (-0.77 – 0.52)	0.69
Block Tapping Test forward (score)	89	0.11 (-0.26 – 0.47)	-0.07 (-0.46 – 0.32)	-0.18 (-0.72 – 0.36)	0.51
Block Tapping Test backward (score)	89	0.29 (-0.08 – 0.65)	-0.13 (-0.52 – 0.26)	-0.42 (-0.95 – 0.12)	0.13
Digit Symbol (score)	89	0.84 (-0.62 – 2.31)	-0.62 (-2.16 – 0.93)	-1.46 (-3.59 – 0.67)	0.18
Stroop, Interference (sec)	89	-2.33 (-7.52 – 2.85)	1.06 (-4.01 – 6.14)	-3.40 (-10.60 – 3.81)	0.35
Language					
Phonemic Fluency (score)	89	-0.01 (-0.92 – 0.90)	0.23 (-0.80 – 1.25)	0.24 (-1.14 – 1.61)	0.73
Semantic Fluency (score)	89	0.23 (-1.23 – 1.69)	0.40 (-1.27 – 2.07)	0.17 (-2.06 – 2.40)	0.88
Boston Naming Test (score)	89	-0.04 (-0.29 – 0.20)	-0.14 (-0.39 – 0.11)	-0.10 (-0.45 – 0.26)	0.59
Attention					
TAP Alertness, median RT uncued trials (sec)	89	-3.29 (-20.36 – 13.79)	3.75 (-13.49 – 20.99)	-7.04 (-31.35 – 17.28)	0.57
TAP Alertness, median RT cued trials (sec)	89	1.50 (-14.14 – 17.15)	2.66 (-13.18 – 18.49)	-1.15 (-23.40 – 21.09)	0.92
TAP Alertness, Phasic Alertness	89	-0.01 (-0.04 – 0.03)	-0.00 (-0.04 – 0.04)	0.01 (-0.04 – 0.06)	0.77
TAP Divided Attention, auditory median RT (sec)	89	4.99 (-49.52 – 59.50)	-8.98 (-73.68 – 55.71)	13.97 (-73.74 – 101.68)	0.75
TAP Divided Attention, visual median RT (sec)	89	-9.61 (-36.35 – 17.13)	-14.33 (-47.55 – 18.88)	4.73 (-38.96 – 48.41)	0.83
TAP Divided Attention, Omissions (score)	89	0.35 (-0.39 – 1.09)	0.09 (-0.80 – 0.97)	-0.26 (-1.42 – 0.89)	0.65

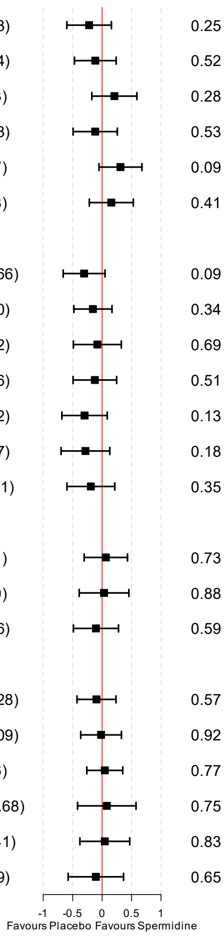

Missing data was imputed using multivariate imputation by chained equations (mice) based on 30 imputed datasets and predictive mean matching. Mean changes of each parameter from baseline to post-intervention assessment with 95% CI are presented for both intervention groups separately. Group differences and P-values result from analysis of covariance models for change in outcome from baseline to post-intervention visit, with intervention group as factor and adjusted for age, sex, and the particular baseline measure. Forest plots indicate mean group differences with 95% CI, standardized by being converted into z-scores. Forest plots and group differences were transformed, if necessary, to yield same direction of effect (positive values: favor spermidine, negative values: favor placebo). Abbreviations: MST, Mnemonic Similarity Task; RT, reaction time; TAP, Test for Attentional Performance battery; TMT, Trail Making Test; VLMT, Verbaler Lern- und Merkfähigkeitstest, German version of the Rey Auditory Verbal Learning Test (AVLT).

© 2022 Schwarz C et al. *JAMA Network Open.*

eFigure 5. Effect of spermidine supplementation on behavioral parameters – per protocol analysis

Parameter	Participants	Mean change placebo (95% CI)	Mean change spermidine (95% CI)	Group differences (95% CI)	P-value
Psycho-affective/worry					
ECog-39 (score)	89	-0.34 (-0.42 – -0.25)	-0.41 (-0.51 – -0.32)	0.08 (-0.05 – 0.21)	0.24
MMQ (score)	89	1.89 (0.62 – 3.16)	1.62 (0.37 – 2.87)	-0.27 (-2.11 – 1.57)	0.77
RSQ-D, Self-focused rumination (score)	89	-0.58 (-1.28 – 0.12)	-0.07 (-0.81 – 0.67)	-0.51 (-1.54 – 0.52)	0.33
RSQ-D, Symptom-focused rumination (score)	89	0.07 (-0.86 – 1.01)	-0.21 (-1.20 – 0.78)	0.29 (-1.08 – 1.65)	0.68
RSQ-D, Distraction (score)	89	-0.03 (-1.03 – 0.96)	-0.68 (-1.73 – 0.38)	-0.65 (-2.13 – 0.84)	0.39
PSWQ (score)	89	-1.88 (-4.44 – 0.69)	-1.39 (-3.94 – 1.17)	-0.49 (-4.27 – 3.29)	0.80
STAI-X1 (score)	89	-0.41 (-2.43 – 1.62)	-0.69 (-2.83 – 1.45)	0.28 (-2.68 – 3.23)	0.85
Lifestyle					
CAI present (score)	89	0.02 (-0.12 – 0.15)	0.12 (-0.03 – 0.26)	0.10 (-0.10 – 0.30)	0.32
PSQI (score)	89	-0.11 (-0.93 – 0.72)	-0.34 (-1.14 – 0.46)	0.23 (-0.94 – 1.40)	0.70
FKA, Lifestyle (score)	89	0.12 (-0.58 – 0.83)	-0.13 (-0.85 – 0.58)	-0.25 (-1.27 – 0.77)	0.62
MEDAS (score)	89	0.12 (-0.37 – 0.60)	0.56 (0.05 – 1.07)	0.44 (-0.26 – 1.15)	0.21
FFQ, Spermidine	89	0.01 (-0.05 – 0.08)	0.03 (-0.03 – 0.09)	0.02 (-0.07 – 0.10)	0.72
FFQ, Spermine	89	0.04 (-0.01 – 0.09)	0.01 (-0.04 – 0.06)	-0.03 (-0.11 – 0.04)	0.37
FFQ, Putrescine	89	-0.02 (-0.06 – 0.02)	-0.03 (-0.07 – 0.01)	-0.00 (-0.06 – 0.06)	0.88
SF-12, Physical health (score)	89	-1.36 (-3.83 – 1.11)	-0.95 (-3.56 – 1.67)	0.41 (-3.21 – 4.03)	0.82
SF-12, Mental health (score)	89	0.65 (-1.80 – 3.10)	0.38 (-2.21 – 2.98)	-0.27 (-3.84 – 3.31)	0.88
WHOQOL-BREF, Physical (score)	89	-0.35 (-4.23 – 3.54)	-0.56 (-4.49 – 3.37)	-0.21 (-5.93 – 5.50)	0.94
WHOQOL-BREF, Psychological (score)	89	0.13 (-2.88 – 3.14)	-0.52 (-3.35 – 2.32)	-0.64 (-5.06 – 3.77)	0.77
WHOQOL-BREF, Social (score)	89	-2.34 (-6.35 – 1.67)	1.79 (-2.07 – 5.65)	4.13 (-1.53 – 9.79)	0.15
WHOQOL-BREF, Environmental (score)	89	1.20 (-1.01 – 3.41)	-0.48 (-2.65 – 1.70)	-1.68 (-4.77 – 1.41)	0.28
WHOQOL-BREF, Overall (score)	89	-2.92 (-7.09 – 1.26)	2.14 (-2.03 – 6.30)	5.05 (-0.87 – 10.97)	0.09

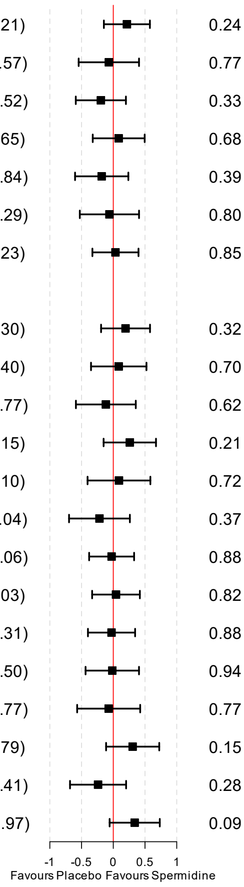

Missing data was imputed using multivariate imputation by chained equations (mice) based on 30 imputed datasets and predictive mean matching. Mean changes of each parameter from baseline to post-intervention assessment with 95% CI are presented for both intervention groups separately. Group differences and P-values result from analysis of covariance models for change in outcome from baseline to post-intervention visit, with intervention group as factor and adjusted for age, sex, and the particular baseline measure. Forest plots indicate mean group differences with 95% CI, standardized by being converted into z-scores. Forest plots and group differences were transformed, if necessary, to yield same direction of effect (positive values: favor spermidine, negative values: favor placebo). Abbreviations: CAI, Cognitive Activity Interview; ECog-39, Everyday Cognition Scales 39; FFQ, Food Frequency Questionnaire; FKA, Freiburger Fragebogen zur körperlichen Aktivität; MEDAS, Mediterranean Diet Adherence Screener; MMQ, Meta Memory Questionnaire; PSQI, Pittsburgh Sleep Quality Index; PSWQ, Penn State Worry Questionnaire; RSQ-D, Response Styles Questionnaire – German Version; SF-12, Short Form Health Survey; STAI-X1, State-Trait Anxiety Inventory – X1; WHOQOL-BREF, World Health Organization Quality of Life.

eFigure 6. Effect of spermidine supplementation on physiological parameters – per protocol analysis

Parameter	Participants	Mean change placebo (95% CI)	Mean change spermidine (95% CI)	Group differences (95% CI)	P-value
Leukocytes (/nl)	89	0.21 (-0.26 – 0.69)	0.09 (-0.39 – 0.58)	0.12 (-0.56 – 0.80)	0.73
Erythrocytes (/pg)	89	-0.03 (-0.14 – 0.09)	-0.01 (-0.12 – 0.11)	0.02 (-0.13 – 0.18)	0.79
Quick (%)	89	-3.49 (-8.36 – 1.39)	-7.50 (-12.75 – -2.24)	-4.01 (-11.05 – 3.03)	0.26
INR	89	0.03 (-0.05 – 0.10)	0.08 (-0.01 – 0.17)	0.05 (-0.06 – 0.17)	0.35
Fibrinogen (g/l)	89	-0.04 (-0.24 – 0.17)	0.04 (-0.19 – 0.27)	-0.07 (-0.37 – 0.23)	0.62
Cholesterol (mg/dl)	89	-0.78 (-11.16 – 9.60)	-11.53 (-22.20 – -0.86)	10.75 (-3.85 – 25.35)	0.15
HDL cholesterol (mg/dl)	89	-1.89 (-6.31 – 2.52)	-0.72 (-5.19 – 3.75)	1.17 (-5.07 – 7.42)	0.71
LDL cholesterol (mg/dl)	89	-0.27 (-10.46 – 9.92)	-11.41 (-21.59 – -1.23)	11.14 (-2.77 – 25.06)	0.11
Triglycerides (mg/dl)	89	1.56 (-13.92 – 17.05)	3.78 (-13.61 – 21.17)	-2.22 (-25.80 – 21.37)	0.85
Glucose (mg/dl)	89	-0.99 (-4.63 – 2.65)	-2.79 (-6.86 – 1.28)	1.80 (-3.50 – 7.09)	0.50
HbA1c (%)	89	0.14 (0.03 – 0.25)	0.06 (-0.05 – 0.17)	0.08 (-0.07 – 0.23)	0.27
Insulin (mU/l)	89	0.73 (-4.24 – 5.69)	3.20 (-2.34 – 8.73)	2.47 (-5.26 – 10.20)	0.52
Homocysteine (µmol/l)	89	0.00 (-1.62 – 1.62)	-0.15 (-1.74 – 1.44)	0.15 (-2.07 – 2.38)	0.89
Liver					
AST (U/l)	89	-1.59 (-4.14 – 0.96)	-1.82 (-4.06 – 0.42)	0.24 (-2.99 – 3.47)	0.88
ALT (U/l)	89	-0.75 (-3.36 – 1.86)	-0.18 (-3.07 – 2.72)	-0.57 (-4.49 – 3.35)	0.77
Kidney					
Creatinine (mg/dl)	89	0.05 (0.00 – 0.10)	0.05 (-0.01 – 0.11)	0.00 (-0.07 – 0.08)	0.90
eGFR	89	-3.26 (-6.30 – -0.21)	-3.35 (-6.59 – -0.11)	-0.09 (-4.77 – 4.58)	0.97
Inflammation/vascular injury					
CRP (mg/l)	89	-1.26 (-2.19 – -0.34)	-1.00 (-2.02 – 0.02)	-0.26 (-1.40 – 0.87)	0.65
SAA (mg/l)	89	-3.66 (-5.71 – -1.60)	-3.83 (-5.90 – -1.77)	0.18 (-1.47 – 1.82)	0.83
sICAM-1 (ng/ml)	89	13.69 (-23.37 – 50.75)	-32.62 (-67.73 – 2.49)	46.30 (-3.60 – 96.21)	0.07
sVCAM-1 (ng/ml)	89	20.49 (-24.48 – 65.45)	-2.65 (-47.28 – 41.98)	23.13 (-40.08 – 86.34)	0.47
IFN-gamma (pg/ml)	89	-0.07 (-1.63 – 1.50)	1.42 (-0.25 – 3.09)	-1.49 (-3.80 – 0.81)	0.20
IL-10 (pg/ml)	89	0.03 (-0.06 – 0.12)	0.03 (-0.05 – 0.12)	0.00 (-0.12 – 0.13)	0.95
IL-6 (pg/ml)	89	0.08 (-0.03 – 0.18)	0.12 (0.01 – 0.24)	-0.05 (-0.20 – 0.11)	0.56
IL-8 (pg/ml)	89	0.76 (-0.99 – 2.51)	0.07 (-1.79 – 1.93)	0.69 (-1.77 – 3.14)	0.58
TNFalpha (pg/ml)	89	0.02 (-0.16 – 0.19)	0.02 (-0.16 – 0.19)	0.00 (-0.25 – 0.25)	1.00
Polyamines					
Spermidine (µM)	89	0.15 (-0.53 – 0.84)	0.08 (-0.62 – 0.77)	-0.07 (-1.05 – 0.90)	0.88
Spermine (µM)	89	0.09 (-0.73 – 0.91)	-0.02 (-0.88 – 0.84)	-0.11 (-1.25 – 1.03)	0.85
L-Ornithine (µM)	89	1.62 (-3.60 – 6.85)	2.22 (-3.71 – 8.15)	0.60 (-7.32 – 8.52)	0.88
Cardiovascular risk					
SBP (mmHg)	89	2.19 (-1.22 – 5.60)	-1.71 (-5.31 – 1.90)	3.90 (-1.09 – 8.89)	0.12
DBP (mmHg)	89	-1.89 (-4.10 – 0.31)	-3.16 (-5.49 – -0.83)	1.26 (-1.95 – 4.48)	0.44
Heart rate (bpm)	89	-0.63 (-2.84 – 1.57)	-0.19 (-2.52 – 2.14)	-0.45 (-3.66 – 2.77)	0.78
Weight (kg)	89	-0.52 (-1.42 – 0.38)	-0.24 (-1.19 – 0.72)	-0.28 (-1.60 – 1.04)	0.67

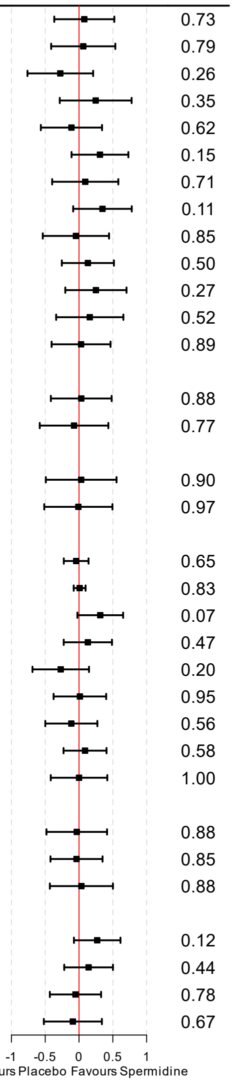

Missing data was imputed using multivariate imputation by chained equations (mice) based on 30 imputed datasets and predictive mean matching. Mean changes of each parameter from baseline to post-intervention assessment with 95% CI are presented for both intervention groups separately. Group differences and P-values result from analysis of covariance models for change in outcome from baseline to post-intervention visit, with intervention group as factor and adjusted for age, sex, and the particular baseline measure. Forest plots indicate mean group differences with 95% CI, standardized by being converted into z-scores. Forest plots and group differences were transformed, if necessary, to yield same direction of effect (positive values: favor spermidine, negative values: favor placebo). Abbreviations: ALT, alanine aminotransferase; AST, aspartate aminotransferase; CRP, c-reactive protein; DBP, diastolic blood pressure; eGFR, estimated glomerular filtration rate; HbA1c, hemoglobin A1c; HDL, high-density lipoprotein; IL, interleukin; INR, international normalized ratio of blood clotting; LDL, low-density lipoprotein; IFN-gamma, interferon gamma; SAA, Serum amyloid A; SBP, systolic blood pressure; sICAM-1, soluble intercellular adhesion molecule-1; sVCAM-1, soluble vascular cell adhesion molecule-1; TNF-alpha, tumor necrosis factor alpha.

© 2022 Schwarz C et al. *JAMA Network Open.*

eFigure 7. Effect of spermidine supplementation on neuropsychological parameters – per protocol *plus* analysis

Parameter	Participants	Mean change placebo (95% CI)	Mean change spermidine (95% CI)	Group differences (95% CI)	P-value
Memory					
MST, Mnemonic discrimination (score)	74	0.01 (-0.04 – 0.05)	-0.03 (-0.09 – 0.02)	-0.04 (-0.11 – 0.03)	0.28
MST, Recognition memory (score)	74	0.03 (-0.01 – 0.07)	0.02 (-0.03 – 0.07)	-0.01 (-0.08 – 0.06)	0.76
VLMT, Learning ability (score)	74	3.12 (1.19 – 5.05)	5.01 (3.03 – 6.99)	1.89 (-0.89 – 4.66)	0.18
VLMT, Delayed recall, forgotten words (score)	74	-0.35 (-1.06 – 0.36)	0.06 (-0.67 – 0.79)	-0.41 (-1.43 – 0.61)	0.42
VLMT, Recognition (score)	74	-0.10 (-1.07 – 0.87)	0.91 (-0.08 – 1.91)	1.01 (-0.38 – 2.41)	0.15
Doors and People Test, Visual recognition (score)	74	0.63 (-0.14 – 1.40)	1.08 (0.29 – 1.87)	0.45 (-0.65 – 1.56)	0.42
Executive function					
TMT B (sec)	74	-7.27 (-15.88 – 1.33)	6.61 (-2.20 – 15.42)	-13.89 (-26.25 – -1.53)	0.03
Digit Span forward (score)	74	0.38 (-0.04 – 0.81)	0.04 (-0.40 – 0.47)	-0.35 (-0.95 – 0.26)	0.26
Digit Span backward (score)	74	-0.15 (-0.64 – 0.34)	-0.59 (-1.09 – -0.09)	-0.44 (-1.15 – 0.26)	0.21
Block Tapping Test forward (score)	74	0.21 (-0.19 – 0.62)	-0.10 (-0.51 – 0.32)	-0.31 (-0.89 – 0.27)	0.29
Block Tapping Test backward (score)	74	0.28 (-0.15 – 0.72)	-0.08 (-0.53 – 0.36)	-0.37 (-0.99 – 0.25)	0.24
Digit Symbol (score)	74	1.55 (-0.04 – 3.13)	-0.69 (-2.32 – 0.93)	-2.24 (-4.51 – 0.03)	0.05
Stroop, Interference (sec)	74	-1.65 (-6.38 – 3.07)	0.13 (-4.71 – 4.97)	-1.79 (-8.56 – 4.98)	0.60
Language					
Phonemic Fluency (score)	74	0.53 (-0.45 – 1.51)	0.31 (-0.77 – 1.40)	-0.21 (-1.69 – 1.26)	0.78
Semantic Fluency (score)	74	0.52 (-1.18 – 2.22)	0.74 (-1.15 – 2.63)	0.22 (-2.34 – 2.78)	0.86
Boston Naming Test (score)	74	0.03 (-0.22 – 0.28)	-0.04 (-0.28 – 0.20)	-0.07 (-0.42 – 0.27)	0.67
Attention					
TAP Alertness, median RT uncued trials (sec)	74	-2.01 (-18.63 – 14.61)	10.30 (-6.73 – 27.33)	-12.31 (-36.12 – 11.50)	0.31
TAP Alertness, median RT cued trials (sec)	74	0.46 (-13.35 – 14.28)	5.53 (-8.63 – 19.69)	-5.07 (-24.88 – 14.74)	0.61
TAP Alertness, Phasic Alertness	74	-0.00 (-0.04 – 0.03)	0.00 (-0.04 – 0.04)	0.01 (-0.05 – 0.06)	0.76
TAP Divided Attention, auditory median RT (sec)	74	15.26 (-41.28 – 71.80)	-2.75 (-74.21 – 68.70)	18.01 (-73.22 – 109.25)	0.69
TAP Divided Attention, visual median RT (sec)	74	-10.10 (-39.75 – 19.55)	-12.42 (-49.48 – 24.63)	2.32 (-45.35 – 49.99)	0.92
TAP Divided Attention, Omissions (score)	74	0.52 (-0.25 – 1.29)	-0.17 (-1.13 – 0.79)	-0.69 (-1.94 – 0.56)	0.27

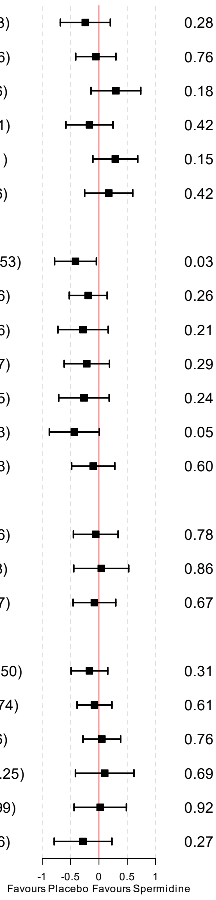

Missing data was imputed using multivariate imputation by chained equations (mice) based on 30 imputed datasets and predictive mean matching. Mean changes of each parameter from baseline to post-intervention assessment with 95% CI are presented for both intervention groups separately. Group differences and P-values result from analysis of covariance models for change in outcome from baseline to post-intervention visit, with intervention group as factor and adjusted for age, sex, and the particular baseline measure. Forest plots indicate mean group differences with 95% CI, standardized by being converted into z-scores. Forest plots and group differences were transformed, if necessary, to yield same direction of effect (positive values: favor spermidine, negative values: favor placebo). Abbreviations: MST, Mnemonic Similarity Task; RT, reaction time; TAP, Test for Attentional Performance battery; TMT, Trail Making Test; VLMT, Verbaler Lern- und Merkfähigkeitstest, German version of the Rey Auditory Verbal Learning Test (AVLT).

© 2022 Schwarz C et al. *JAMA Network Open.*

eFigure 8. Effect of spermidine supplementation on behavioral parameters – per protocol *plus* analysis

Parameter	Participants	Mean change placebo (95% CI)	Mean change spermidine (95% CI)	Group differences (95% CI)	P-value
Psycho-affective/worry					
ECog-39 (score)	74	-0.37 (-0.46 – -0.28)	-0.40 (-0.49 – -0.31)	0.03 (-0.09 – 0.15)	0.64
MMQ (score)	74	1.85 (0.53 – 3.16)	1.92 (0.62 – 3.23)	0.08 (-1.82 – 1.97)	0.93
RSQ-D, Self-focused rumination (score)	74	-0.63 (-1.43 – 0.18)	-0.36 (-1.18 – 0.47)	-0.27 (-1.44 – 0.89)	0.64
RSQ-D, Symptom-focused rumination (score)	74	-0.12 (-1.16 – 0.92)	-0.36 (-1.43 – 0.71)	0.24 (-1.25 – 1.74)	0.75
RSQ-D, Distraction (score)	74	0.16 (-1.00 – 1.31)	-0.46 (-1.65 – 0.73)	-0.62 (-2.31 – 1.07)	0.47
PSWQ (score)	74	-1.75 (-4.59 – 1.08)	-1.24 (-4.02 – 1.54)	-0.51 (-4.64 – 3.62)	0.81
STAI-X1 (score)	74	-0.14 (-2.51 – 2.24)	-0.61 (-3.05 – 1.83)	0.47 (-2.94 – 3.88)	0.78
Lifestyle					
CAI present (score)	74	-0.03 (-0.19 – 0.13)	0.12 (-0.04 – 0.28)	0.15 (-0.07 – 0.38)	0.18
PSQI (score)	74	-0.13 (-0.99 – 0.73)	-0.49 (-1.29 – 0.31)	0.36 (-0.84 – 1.57)	0.55
FKA, Lifestyle (score)	74	0.13 (-0.61 – 0.87)	-0.20 (-0.94 – 0.54)	-0.34 (-1.40 – 0.73)	0.53
MEDAS (score)	74	0.12 (-0.40 – 0.65)	0.71 (0.18 – 1.25)	0.59 (-0.16 – 1.34)	0.12
FFQ, Spermidine	74	0.00 (-0.06 – 0.07)	0.04 (-0.01 – 0.10)	0.04 (-0.05 – 0.13)	0.35
FFQ, Spermine	74	0.02 (-0.03 – 0.07)	0.02 (-0.03 – 0.07)	0.00 (-0.07 – 0.07)	0.99
FFQ, Putrescine	74	-0.02 (-0.06 – 0.02)	-0.03 (-0.07 – 0.01)	-0.01 (-0.07 – 0.05)	0.75
SF-12, Physical health (score)	74	-0.63 (-3.42 – 2.16)	-0.91 (-3.77 – 1.95)	-0.28 (-4.34 – 3.78)	0.89
SF-12, Mental health (score)	74	0.46 (-2.34 – 3.26)	0.43 (-2.44 – 3.29)	-0.03 (-4.04 – 3.97)	0.99
WHOQOL-BREF, Physical (score)	74	0.52 (-3.70 – 4.75)	-0.47 (-4.63 – 3.69)	-0.99 (-7.15 – 5.17)	0.75
WHOQOL-BREF, Psychological (score)	74	1.38 (-1.95 – 4.71)	0.15 (-2.89 – 3.19)	-1.23 (-6.08 – 3.61)	0.61
WHOQOL-BREF, Social (score)	74	-2.44 (-6.87 – 1.99)	2.99 (-1.15 – 7.13)	5.44 (-0.75 – 11.63)	0.08
WHOQOL-BREF, Environmental (score)	74	2.18 (-0.27 – 4.63)	-0.77 (-3.16 – 1.62)	-2.95 (-6.38 – 0.48)	0.09
WHOQOL-BREF, Overall (score)	74	-3.05 (-7.70 – 1.60)	2.56 (-1.92 – 7.04)	5.61 (-0.92 – 12.14)	0.09

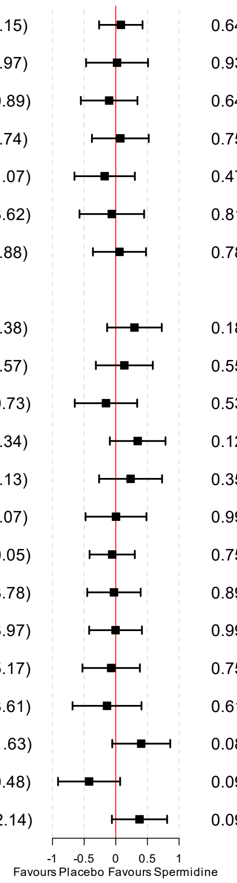

Missing data was imputed using multivariate imputation by chained equations (mice) based on 30 imputed datasets and predictive mean matching. Mean changes of each parameter from baseline to post-intervention assessment with 95% CI are presented for both intervention groups separately. Group differences and P-values result from analysis of covariance models for change in outcome from baseline to post-intervention visit, with intervention group as factor and adjusted for age, sex, and the particular baseline measure. Forest plots indicate mean group differences with 95% CI, standardized by being converted into z-scores. Forest plots and group differences were transformed, if necessary, to yield same direction of effect (positive values: favor spermidine, negative values: favor placebo). Abbreviations: CAI, Cognitive Activity Interview; ECog-39, Everyday Cognition Scales 39; FFQ, Food Frequency Questionnaire; FKA, Freiburger Fragebogen zur körperlichen Aktivität; MEDAS, Mediterranean Diet Adherence Screener; MMQ, Meta Memory Questionnaire; PSQI, Pittsburgh Sleep Quality Index; PSWQ, Penn State Worry Questionnaire; RSQ-D, Response Styles Questionnaire – German Version; SF-12, Short Form Health Survey; STAI-X1, State-Trait Anxiety Inventory – X1; WHOQOL-BREF, World Health Organization Quality of Life.

© 2022 Schwarz C et al. *JAMA Network Open.*

eFigure 9. Effect of spermidine supplementation on physiological parameters – per protocol *plus* analysis

Parameter	Participants	Mean change placebo (95% CI)	Mean change spermidine (95% CI)	Group differences (95% CI)	P-value
Leukocytes (/nl)	74	0.09 (-0.40 – 0.57)	0.11 (-0.42 – 0.63)	-0.02 (-0.73 – 0.69)	0.95
Erythrocytes (/pg)	74	-0.05 (-0.15 – 0.06)	-0.00 (-0.12 – 0.12)	0.04 (-0.11 – 0.20)	0.57
Quick (%)	74	-3.82 (-9.01 – 1.37)	-7.08 (-12.05 – -2.10)	-3.25 (-10.57 – 4.06)	0.37
INR	74	0.03 (-0.06 – 0.11)	0.08 (-0.02 – 0.17)	0.05 (-0.07 – 0.17)	0.39
Fibrinogen (g/l)	74	-0.06 (-0.24 – 0.12)	-0.03 (-0.24 – 0.19)	-0.03 (-0.30 – 0.24)	0.83
Cholesterol (mg/dl)	74	0.95 (-9.73 – 11.62)	-10.59 (-22.10 – 0.93)	11.53 (-3.81 – 26.88)	0.14
HDL cholesterol (mg/dl)	74	-2.19 (-6.33 – 1.95)	-0.31 (-4.76 – 4.14)	1.88 (-4.19 – 7.96)	0.54
LDL cholesterol (mg/dl)	74	1.30 (-9.08 – 11.69)	-10.89 (-21.96 – 0.18)	12.20 (-2.70 – 27.10)	0.11
Triglycerides (mg/dl)	74	0.38 (-15.55 – 16.31)	5.37 (-13.21 – 23.95)	-4.99 (-30.26 – 20.28)	0.69
Glucose (mg/dl)	74	-2.46 (-5.73 – 0.80)	-1.83 (-5.72 – 2.06)	-0.63 (-5.73 – 4.47)	0.80
HbA1c (%)	74	0.10 (0.01 – 0.20)	0.07 (-0.03 – 0.17)	0.03 (-0.10 – 0.17)	0.64
Insulin (mU/l)	74	0.47 (-4.78 – 5.72)	3.88 (-2.29 – 10.05)	3.41 (-4.78 – 11.60)	0.41
Homocysteine (µmol/l)	74	0.04 (-1.68 – 1.76)	-0.18 (-1.93 – 1.56)	0.22 (-2.19 – 2.64)	0.85
Liver					
AST (U/l)	74	-1.60 (-3.71 – 0.51)	-1.69 (-4.07 – 0.70)	0.09 (-3.01 – 3.19)	0.96
ALT (U/l)	74	-1.04 (-3.66 – 1.59)	0.15 (-2.99 – 3.28)	-1.18 (-5.29 – 2.93)	0.57
Kidney					
Creatinine (mg/dl)	74	0.05 (0.00 – 0.10)	0.06 (-0.01 – 0.12)	-0.01 (-0.09 – 0.08)	0.89
eGFR	74	-3.39 (-6.38 – -0.40)	-4.20 (-7.68 – -0.73)	-0.82 (-5.47 – 3.84)	0.73
Inflammation/vascular injury					
CRP (mg/l)	74	-1.09 (-2.10 – -0.07)	-0.90 (-1.99 – 0.19)	-0.19 (-1.44 – 1.06)	0.77
SAA (mg/l)	74	-4.22 (-6.22 – -2.22)	-4.32 (-6.39 – -2.26)	0.10 (-1.35 – 1.56)	0.89
sICAM-1 (ng/ml)	74	25.72 (-11.22 – 62.66)	-30.46 (-67.85 – 6.94)	56.18 (5.59 – 106.77)	0.03
sVCAM-1 (ng/ml)	74	34.20 (-12.13 – 80.54)	-4.83 (-53.30 – 43.65)	39.03 (-28.00 – 106.06)	0.25
IFN-gamma (pg/ml)	74	-0.21 (-1.61 – 1.19)	1.02 (-0.50 – 2.53)	-1.22 (-3.35 – 0.90)	0.25
IL-10 (pg/ml)	74	-0.01 (-0.08 – 0.06)	0.02 (-0.05 – 0.10)	0.03 (-0.08 – 0.14)	0.57
IL-6 (pg/ml)	74	0.06 (-0.06 – 0.17)	0.11 (-0.01 – 0.23)	-0.05 (-0.22 – 0.11)	0.51
IL-8 (pg/ml)	74	-0.30 (-1.46 – 0.86)	-0.09 (-1.50 – 1.32)	-0.21 (-1.97 – 1.54)	0.81
TNFalpha (pg/ml)	74	-0.01 (-0.15 – 0.13)	-0.02 (-0.17 – 0.13)	0.02 (-0.19 – 0.22)	0.87
Polyamines					
Spermidine (µM)	74	0.03 (-0.70 – 0.76)	0.04 (-0.72 – 0.80)	0.01 (-1.04 – 1.07)	0.98
Spermine (µM)	74	-0.00 (-0.86 – 0.86)	-0.18 (-1.14 – 0.77)	-0.18 (-1.45 – 1.09)	0.78
L-Ornithine (µM)	74	2.61 (-2.27 – 7.49)	4.92 (-1.09 – 10.93)	2.31 (-5.32 – 9.94)	0.54
Cardiovascular risk					
SBP (mmHg)	74	0.24 (-3.46 – 3.94)	-1.93 (-5.73 – 1.86)	2.17 (-3.16 – 7.50)	0.42
DBP (mmHg)	74	-2.28 (-4.84 – 0.29)	-2.85 (-5.48 – -0.22)	0.57 (-3.10 – 4.25)	0.76
Heart rate (bpm)	74	-0.56 (-3.14 – 2.02)	-0.08 (-2.72 – 2.56)	-0.48 (-4.17 – 3.21)	0.80
Weight (kg)	74	-0.31 (-1.35 – 0.73)	-0.12 (-1.19 – 0.95)	-0.19 (-1.69 – 1.31)	0.80

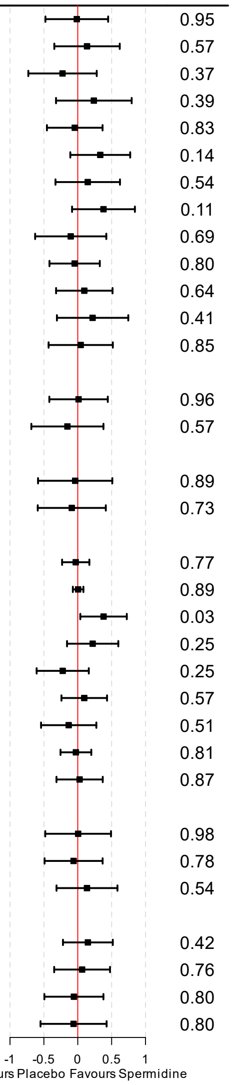

Missing data was imputed using multivariate imputation by chained equations (mice) based on 30 imputed datasets and predictive mean matching. Mean changes of each parameter from baseline to post-intervention assessment with 95% CI are presented for both intervention groups separately. Group differences and P-values result from analysis of covariance models for change in outcome from baseline to post-intervention visit, with intervention group as factor and adjusted for age, sex, and the particular baseline measure. Forest plots indicate mean group differences with 95% CI. Forest plots and group differences were transformed, if necessary, to yield same direction of effect (positive values: favor spermidine, negative values: favor placebo). Abbreviations: ALT, alanine aminotransferase; AST, aspartate aminotransferase; CRP, c-reactive protein; DBP, diastolic blood pressure; eGFR, estimated glomerular filtration rate; HbA1c, hemoglobin A1c; HDL, high-density lipoprotein; IL, interleukin; INR, international normalized ratio of blood clotting; LDL, low-density lipoprotein; IFN-gamma, interferon gamma; SAA, Serum amyloid A; SBP, systolic blood pressure; sICAM-1, soluble intercellular adhesion molecule-1; sVCAM-1, soluble vascular cell adhesion molecule-1; TNF-alpha, tumor necrosis factor alpha.

© 2022 Schwarz C et al. *JAMA Network Open.*

eReferences.

1. Folstein MF, Folstein SE, McHugh PR. “Mini-mental state”: a practical method for grading the cognitive state of patients for the clinician. *J Psychiatr Res.* 1975;12(3):189-198.
2. Lawton MP, Brody EM. Assessment of older people: self-maintaining and instrumental activities of daily living. *Gerontologist.* 1969;9(3):179-186.
3. Yesavage JA, Brink TL, Rose TL, et al. Development and validation of a geriatric depression screening scale: a preliminary report. *J Psychiatr Res.* 1982;17(1):37-49.
4. Wirth M, Schwarz C, Benson G, et al. Effects of spermidine supplementation on cognition and biomarkers in older adults with subjective cognitive decline (SmartAge)-study protocol for a randomized controlled trial. *Alzheimers Res Ther.* 2019;11(1):36.
5. Jessen F, Amariglio RE, van Boxtel M, et al. A conceptual framework for research on subjective cognitive decline in preclinical Alzheimer's disease. *Alzheimers Dement.* 2014;10(6):844-852.
6. Yassa MA, Lacy JW, Stark SM, Albert MS, Gallagher M, Stark CE. Pattern separation deficits associated with increased hippocampal CA3 and dentate gyrus activity in nondemented older adults. *Hippocampus.* 2011;21(9):968-979.
7. Stark SM, Yassa MA, Lacy JW, Stark CE. A task to assess behavioral pattern separation (BPS) in humans: Data from healthy aging and mild cognitive impairment. *Neuropsychologia.* 2013;51(12):2442-2449.
8. Marks SM, Lockhart SN, Baker SL, Jagust WJ. Tau and β-Amyloid Are Associated with Medial Temporal Lobe Structure, Function, and Memory Encoding in Normal Aging. *J Neurosci.* 2017;37(12):3192-3201.
9. Wirth M, Benson G, Schwarz C, et al. The effect of spermidine on memory performance in older adults at risk for dementia: A randomized controlled trial. *Cortex.* 2018;109:181-188.
10. Scarpina F, Tagini S. The Stroop Color and Word Test. *Front Psychol.* 2017;8:557-557.
11. Eisenberg T, Abdellatif M, Schroeder S, et al. Cardioprotection and lifespan extension by the natural polyamine spermidine. *Nat Med.* 2016;22(12):1428-1438.
12. Kiechl S, Pechlaner R, Willeit P, et al. Higher spermidine intake is linked to lower mortality: a prospective population-based study. *Am J Clin Nutr.* 2018;108(2):371-380.
13. Schwarz C, Horn N, Benson G, et al. Spermidine intake is associated with cortical thickness and hippocampal volume in older adults. *Neuroimage.* 2020;221:117132.
14. Schwarz C, Stekovic S, Wirth M, et al. Safety and tolerability of spermidine supplementation in mice and older adults with subjective cognitive decline. *Aging (Albany NY).* 2018;10(1):19.
15. Magnes C, Fauland A, Gander E, et al. Polyamines in biological samples: rapid and robust quantification by solid-phase extraction online-coupled to liquid chromatography-tandem mass spectrometry. *J Chromatogr A.* 2014;1331(100):44-51.

© 2022 Schwarz C et al. *JAMA Network Open.*
