# Supplementary material for: Effects of Spermidine Supplementation on Cognition and Biomarkers in Older Adults With Subjective Cognitive Decline: A Randomized Clinical Trial
Source: JAMA Netw Open. 2022 May 26;5(5):e2213875. doi: 10.1001/jamanetworkopen.2022.13875 (PMC9136623; doi:10.1001/jamanetworkopen.2022.13875)
Supplement: Supplement 3. — Data Sharing Statement [file jamanetwopen-e2213875-s003.pdf]

Data Sharing Statement

Schwarz. Effects of Spermidine Supplementation on Cognition and Biomarkers in Older Adults With Subjective Cognitive Decline. *JAMA Netw Open*. Published May 26, 2022. doi:10.1001/jamanetworkopen.2022.13875

Data
Data available: No
